# Bacterial cell membrane models: choosing the lipid composition

**DOI:** 10.1039/d5sm00378d

**Published:** 2025-08-26

**Authors:** Alexandra L. Martin, Philip N. Jemmett, Thomas Howitt, Mary H. Wood, Liam R. Cox, Timothy R. Dafforn, Mario Campana, Rebecca J. L. Welbourn, Maximilian W. A. Skoda, Luke A. Clifton, Hadeel Hussain, Jonathan L. Rawle, Francesco Carlà, Christopher L. Nicklin, Thomas Arnold, Sarah L. Horswell

**Affiliations:** a School of Chemistry, University of Birmingham Edgbaston Birmingham B15 2TT UK s.l.horswell@bham.ac.uk; b School of Biosciences, University of Birmingham Edgbaston Birmingham B15 2TT UK; c ISIS Pulsed Neutron and Muon Source, Science and Technology Facilities Council, Rutherford Appleton Laboratory Harwell Oxfordshire OX11 0QX UK tom.arnold@ess.eu; d Neutron Scattering Division, Oak Ridge National Laboratory Oak Ridge Tennessee 37831 USA; e Diamond Light Source, Harwell Science and Innovation Campus, Chilton Didcot Oxfordshire OX11 0DE UK; f European Spallation Source ERIC PO Box 176 SE-221 00 Lund Sweden; g Department of Chemistry, University of Bath Claverton Down Bath BA2 7AY UK

## Abstract

The reasons for the wide diversity of lipids found in natural cell membranes are still not fully understood but could potentially be exploited in treating disease and infection. This study aims to establish whether charge alone or specific chemical structure of an anionic lipid headgroup determines the structure and properties of model bacterial cell membranes. We compare different compositions of a zwitterionic lipid di-myristoyl phosphatidylethanolamine (DMPE) and two anionic lipids, di-myristoyl phosphatidylglycerol (DMPG) and tetra-myristoyl cardiolipin (TMCL). TMCL has a distinct condensing effect, increasing packing and decreasing the pressures of the phase transitions. Although relatively well solvated itself, TMCL does not substantially alter the solvation of mixed monolayers or bilayers. DMPE:TMCL mixtures have very similar electrochemical behaviour to mixtures of DMPE with di-myristoyl phosphatidylserine (DMPS) but DMPE:DMPG bilayers have greater surface charges. A ternary mixture representing an *Escherichia coli* membrane has similar electrochemical response to but is more tightly packed than DMPE:DMPG. These results establish the importance of the anionic lipid in modelling different types of cell membranes: DMPG will be required in model bacterial membranes and should not be replaced with DMPS. Even very small amounts of CL will have a measurable effect on structure, so its inclusion is important. Our results also highlight the importance of diverse techniques in understanding membrane behaviour: reflectivity measurements of monolayers over a range of surface pressure provide excellent insight into the electrochemical responses of lipid bilayers, while surface diffraction and infrared spectroscopy are much more sensitive to differences in packing between lipids.

## Abstract 2

Each lipid type in a model membrane influences membrane properties.

## Graphical abstract

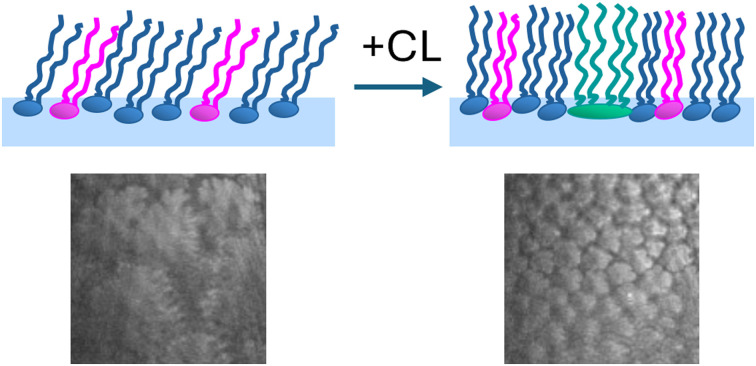

## 1. Introduction

Lipids are one of the building blocks of life: they play rôles in the structure of biological cell membranes, signalling processes, transporting cargo and energy storage.^1^ In the cell membrane, the amphiphilic nature of glycerophospholipids and sphingolipids causes them to assemble into a bilayer structure, which acts as a barrier that enables the cell to control flow of ions and molecules across the membrane through a variety of protein channels.^1^ The composition of the membrane varies with the organism, the cell type and the membrane type^1–3^ but the reasons for the wide diversity of lipid compounds in nature are not yet fully understood. The differences in composition between mammalian plasma cell membranes and bacterial membranes are being explored as a potential target for antimicrobial agents.^4–6^ Bacterial membranes are often negatively charged, while most mammalian membranes segregate their anionic lipids in the inner leaflet.^1–3^ Many organisms have evolved to produce small, positively charged peptides to protect themselves from infection; these are attracted to and disrupt the negatively charged membranes of bacteria, leading to cell death.^4–8^ Peptides have also been shown to target (different) negatively charged lipids expressed in tumour cells.^9,10^ If we can understand these processes, we will be able to design more potent and more selective membrane disrupters for therapeutic applications. In order to do this, we need to understand the interactions between the different lipid components of different membrane types and how they influence the overall membrane structure and properties. This fundamental understanding can also inform the development of other membrane disrupters^11^ and other applications of lipids in healthcare, which include delivery of drugs, nanoparticles or RNA into cells.^12–17^

Most bacterial membranes are mixtures of phosphatidylethanolamine (PE), phosphatidylglycerol (PG) and cardiolipin (CL),^1,18–21^ with the composition depending on the organism.^18–20^ For example, Gram-positive bacteria tend to contain proportionately less PE than Gram-negative bacteria and some species do not contain PE at all.^21–23^ The composition can also vary with the stage of the cell cycle^24^ and respond to changes in the temperature or environment.^1,21,25,26^ PE has a zwitterionic headgroup, while PG and CL are anionic. Each of these lipids has hydrogen bond donors and acceptors and so hydrogen bonds can form directly between neighbouring headgroups. PE has been shown to be essential for protein translocation in *Escherichia coli* (*E. coli*).^27^ PG is thought to be important in membrane permeability^28^ and controlling protein–membrane interactions.^21^ CL is thought to play a structural rôle in membranes^29^ and it has also been shown to be concentrated around the poles and septa of rod-shaped bacteria.^30^ This localisation may be a result of its shape—the headgroup area is relatively small compared with that projected by its four tailgroups—and may be important in controlling protein function.^31^ A rôle for CL as a proton trap for oxidative phosphorylation has also been proposed.^32^ Recent methodology designed to extract membrane proteins shows specific enrichment or depletion of these lipid types around different bacterial proteins.^33^ Mammalian cells, on the other hand, contain little PG, which appears to be mainly a synthetic precursor to other lipids^34^ or a component of lung surfactant.^35^ Instead, they produce phosphatidylserine (PS), which is most commonly found in the cytosolic leaflet of the plasma membrane^1–3,34^ or in the nuclear membrane,^36^ while CL in mammalian cells is predominantly found in mitochondrial membranes.^34^ Understanding the different expression of these lipids could provide exploitable targets for treating mitochondrial disorders or for antimicrobial research.

Model membranes provide a useful tool to disentangle the complexity of the interactions between different lipids and to investigate the interactions of potential therapeutic molecules with the membrane.^37–40^ Calorimetric and spectroscopic studies of polymorphic phase behaviour shed light on the thermodynamic properties of lipids and their mixtures, as well as their organisation, solvation and interactions with external agents.^41–45^ Electrochemical experiments provide information on permeability and barrier properties^46–50^ and a wide range of structural probes, such as fluorescence microscopy,^51,52^ atomic force microscopy (AFM),^50,52–54^ vibrational spectroscopy,^55–60^ grazing incidence X-ray diffraction (GIXD)^60–67^ and X-ray and neutron reflectivity (XRR and NR) measurements,^39,50,61,62,68–74^ enable the structures of monolayers and bilayers of lipids and their interactions with peptides or other agents to be characterised in exquisite detail.^50,75,76^ In the present study, we sought to examine the effects on phase behaviour of lipid compositions with different negatively charged headgroups. Surface charge, barrier properties and lipid packing may all affect susceptibility of a membrane to a membrane disrupter so it is important to understand how these factors are affected by composition, in particular whether they are influenced by the charge alone or the specific nature of the anionic headgroup. Keeping the tail length the same, we here compare di-myristoyl phosphatidylethanolamine (DMPE) mixtures containing di-myristoyl phosphatidylglycerol (DMPG) and tetra-myristoyl cardiolipin (TMCL) with our earlier studies of DMPE mixed with di-myristoyl phosphatidylserine (DMPS).^77^ The compositions chosen include mimics of Gram-positive (50 : 50 and 75 : 25 PG : CL) and Gram-negative (PE : PG : CL 80 : 15 : 5) bacterial membranes, which we compare with binary PE mixtures of varying anionic lipid content.

Our results reveal strong similarity between CL and PS lipid films, whereas PG films are less ordered with weaker intermolecular interactions. CL has an ordering effect on both PE and PG, as does PS on PE. Both PG and CL show non-ideal mixing with PE, with more favourable interactions between unlike molecules that may be related to diluting the anionic charge, as PG and CL mix ideally with each other in the solid phase. The results hint at the possibility of similar rôles for CL and PS in different cell types. Further, they show that including both PG and CL in models is important for mimicking bacterial membranes in future studies of bacterial membrane proteins or design and evaluation of potential membrane-targeting antibacterial agents.

## 2. Experimental

### 2.1. Materials

The lipids used in this study were: di-myristoyl phosphatidylethanolamine (DMPE), di-myristoyl phosphatidylglycerol sodium salt (DMPG), tetra-myristoyl cardiolipin sodium salt (TMCL) and the tail-deuterated analogues of DMPE and DMPG (D54-DMPE and D54-DMPG). These were all purchased from Avanti Polar Lipids (Birmingham, AL) and used as received. Solutions of the lipids were prepared in a 9 : 1 v/v ratio of chloroform and methanol (both HPLC grade, Sigma-Aldrich).

Ultrapure water was used throughout (resistivity ≥ 18 MΩ cm, TOC ≤ 5 ppb). This was obtained from a tandem Elix-MilliQ A10 system (Millipore, France) at the University of Birmingham, an Elga Purelab Classic UV system (Elga, U.K.) at Diamond Light Source and a Millipore system at ISIS. Deuterium oxide (99.9% D) from Sigma-Aldrich was used at ISIS for neutron measurements. The subphase used for neutron measurements was either D_2_O or air-contrast-matched water (ACMW, 8% v/v D_2_O in H_2_O). Sodium fluoride (Puratronic, 99.995% metals basis, Alfa Aesar, U.K.) was used to prepare 0.1 M electrolyte solutions in water for electrochemical measurements.

Volumetric glassware was cleaned with piranha solution (**Caution!** This is a highly exothermic process that may cause an explosion!), rinsed with copious quantities of ultrapure water and soaked in ultrapure water overnight. It was further rinsed with ultrapure water immediately before use. All other glassware was cleaned by heating in a 1 : 1 mixture of sulphuric and nitric acids for ∼1 h and rinsing thoroughly with ultrapure water. It was soaked in ultrapure water overnight and rinsed again before use or before drying in a designated clean oven.

### 2.2. Isotherm measurements and bilayer fabrication

Isotherms were recorded with a Nima Langmuir–Blodgett trough of area 600 cm^2^, fitted with Delrin barriers. The subphase temperature was controlled at 19.5 °C with a thermostatted water bath. (At this temperature, DMPG is expected to be in the ripple phase and the other lipid compositions in the gel (L_β_) phase.^41–43^) The subphase used for isotherms and monolayer structural studies (Sections 2.5 and 2.6) was ultrapure water to enable comparison with our previous study of DMPE/DMPS monolayers and bilayers.^77^ This choice was made so that the results could be correlated with electrochemistry measurements, for which the bilayers were deposited from water to match standard practice in previous electrochemistry studies.^57,59,77^ Using water for deposition avoids competitive adsorption of subphase components. The trough was cleaned with chloroform/methanol solution, filled with ultrapure water and allowed to reach thermal equilibrium. The cleanliness of the surface was verified by ensuring the surface pressure did not increase as the area was reduced. Next, a fixed volume (typically 50 μL) of the lipid solution was added dropwise across the surface using a microlitre syringe and the solvent was allowed to evaporate (10 min). Isotherms were then recorded at a compression rate of 25 cm^2^ min^−1^.

Bilayers were deposited onto Au(111) single crystal electrodes. These were cleaned as described in Section 2.3 and immersed in the water subphase, lipid solution was deposited onto the water surface and the monolayer was compressed to a target surface pressure of 40 mN m^−1^. All monolayers are in the solid phase at this pressure, which was chosen for comparison with our previous study.^77^ The Au substrate was drawn vertically through the interface at a speed of 2 mm min^−1^ (Langmuir–Blodgett deposition) and dried in argon for 30 min. Next, it was positioned with the surface horizontal and lowered onto the water surface to deposit the second monolayer (Langmuir–Schaefer or horizontal touch deposition). A Y-type bilayer was formed; these bilayers are stable on the Au(111) surface on the timescale of the electrochemical experiments.

### 2.3. Electrochemical measurements

An all-glass three-electrode cell was used for electrochemical measurements. The working electrode was an Au(111) single crystal (oriented to better than 0.5° (MaTecK GmbH, Jülich, Germany)). It was prepared by flame-annealing as described in the literature.^78^ This entailed flame-annealing to a dull red colour, cooling to ambient temperature, placing a drop of ultrapure water on the surface, heating to remove the drop and immediately replacing the drop with a fresh one. The electrode was transferred to the cell with the drop, to protect the surface from contamination. The counter electrode was a gold coil (99.999%, Alfa Aesar, U.K.). It was cleaned by heating in a Bunsen flame and quenching with ultrapure water. The reference electrode was a saturated calomel electrode (SCE, Hach Lange) and was housed in a separate compartment containing saturated potassium chloride solution. For consistency with the PM-IRRAS measurements, potentials in this work are reported with respect to Ag|AgCl|3 M KCl. The electrolyte was 0.1 M sodium fluoride solution, which was chosen because the ions do not specifically adsorb on Au or interfere with the adsorption of the bilayer. It was also required for electrochemical IR measurements (Section 2.4) to suppress dissolution of the IR windows. The pH of this solution is 8. The electrolyte was purged of oxygen by bubbling argon through for at least 45 min prior to measurements. A blanket of argon was maintained over the electrolyte surface for the duration of the experiment.

A Heka PGStat 590 potentiostat was used for the electrochemical experiments. For differential capacitance measurements, it was coupled with a DSP-7265 lock-in amplifier (Ametek) to provide a superimposed AC signal (frequency 20 Hz, amplitude 5 mV) on top of the applied potential and to separate the in-phase and quadrature components of the current response. The data were acquired using a data acquisition board (M Series, National Instruments) and software kindly provided by Dr A. L. N. Pinheiro (Universidade Tecnologica Federal do Parana, Londrina, Brazil). Chronocoulometry measurements were performed using this software to control the potentiostat and to record the current transients for a series of potential steps, as described previously.^59,79^ The potential was held at a base potential of (−0.01 V) for 1 min and stepped to the potential of interest, where it was held for 3 min to allow equilibrium to be reached. It was then stepped to a desorption potential (−1.05 V) for 0.15 s and returned to the base potential. The process was repeated for a sequence of steps in 0.05 V increments, in the cathodic direction. The current transients were each integrated to give the relative charge density between the potential of interest and the desorption potential. These were then converted to absolute charge densities, using the potential of zero charge (pzc) of the bare Au(111) in 0.1 M NaF. Typical errors in capacitance values are 1–3 μF cm^−2^ and typical standard deviations in charge density values are 1–4 μC cm^−2^ (usually higher in the positive potential range because the relative charge densities are larger in this range).

### 2.4. Electrochemical infrared measurements

*In situ* electrochemical infrared measurements were carried out in a custom-built cell with a Au(111) crystal (oriented to better than 0.5°, MaTecK GmbH, Jülich, Germany) as working electrode, prepared as described in Section 2.3. The counter electrode was a gold ring, concentric to the working electrode, and the reference electrode was a Ag|AgCl|3 M KCl electrode housed in a separate compartment and connected *via* a Luggin. The window was a 1′′ BaF_2_ prism (Crystran Ltd., UK). Prior to use, it was rinsed with methanol and then ultrapure water before cleaning in an ozone chamber. The electrolyte was 0.1 M NaF in D_2_O and spectra were recorded at 19 °C (±1 °C). Spectra were measured with a Bruker Vertex 80v spectrometer combined with a PMA50 module for PM-IRRAS measurements. The module was equipped with a ZnSe 50 kHz optical head (PEM-100, Hinds Instruments, US) and a synchronous sampling demodulator (GWC Technologies) was used to obtain the difference signal from s- and p-polarised light. The half-wave retardation was 2900 cm^−1^, the angle of incidence was 51° and the spectral resolution was 2 cm^−1^. The spectra were analysed as described by Lipkowski and Zamlynny.^80,81^ Fresnel1 software,^82^ kindly provided by Dr V. Zamlynny (Acadia University, Canada) was used to calculate optical constants, to determine electrolyte thickness and to simulate spectra of randomly oriented molecules to enable calculation of chain tilt angles.

### 2.5. Brewster angle microscopy

Brewster angle microscopy (BAM) was performed with a nanofilm EP3SE imaging ellipsometer (Accurion) at Diamond Light Source. This instrument is configured with a 532 nm, 50 mW laser, a ×50 objective, a polariser, an analyser and a CCD camera. p-polarised light was incident upon the water surface in a Nima Langmuir trough, at the Brewster angle for the air|water interface (53.15°). The maximum trough area was 700 cm^2^ and images were acquired at a compression rate of 45 cm^2^ min^−1^. The images presented herein were acquired on the second compression, for consistency with the X-ray measurements.

### 2.6. Reflectivity and diffraction neasurements

Neutron reflectivity (NR) was carried out at the ISIS Pulsed Neutron and Muon Source (Oxfordshire, U.K.), using the INTER^83^ and SURF^84^ beam lines. Both beamlines employed a ^3^He detector. A temperature-controlled 700 cm^2^ Langmuir trough (Nima) was used on each beamline and encased in a box to reduce exchange of D_2_O with atmospheric water. The data were acquired in time of flight mode over two angles on INTER (0.8 and 2.3°) and three angles on SURF (0.36, 0.66, and 1.5°) with a 7% d*q*/*q* resolution. The illuminated footprint was kept constant for all angles and inside the trough barriers. Data reduction was carried out with the in-house software Mantid.^85,86^

X-ray reflectivity (XRR) and grazing incidence X-ray diffraction (GIXD) were carried out at Diamond Light Source (Oxfordshire, U.K.), using the I07 beamline.^87^ A double-crystal deflector system^88^ was used to direct the X-rays (12.5 keV, *λ* = 0.9919 Å) onto the water surface and data were acquired with a Pilatus 100 K detector. A temperature-controlled 700 cm^2^ Langmuir trough (Nima) was used and encased in a helium-filled box, to reduce beam damage and background scattering. XRR was performed over a range of *q*_*z*_ of 0.018–0.6 Å^−1^ (*q*_*z*_ is momentum transfer normal to the surface). XRR data were reduced with an in-house Python script, which applies a footprint correction for over-illumination at low *q*_*z*_, stitches together several measurement regimes of gradually decreasing attenuation and normalises the data to *R* = 1. GIXD was carried out with an X-ray incident angle corresponding to *q*_*z*_ = 0.018 Å^−1^ and a pinhole set-up to allow image acquisition.^64^ Images were acquired at two detector angles to span a *q*_*z*_ range of 0–0.72 Å^−1^ and then scaled and merged using an in-house MATLAB script. To reduce the effects of beam damage, the monolayer was expanded and recompressed between measurements and the sample changed after three or four measurements. XRR was measured in duplicate and GIXD in duplicate or triplicate for each composition and pressure, to ensure reliability of the structural parameters derived. The data reported below are the mean values resulting from fitting the replicate measurements. RasCAL was used to fit neutron and X-ray reflectivity data.^89^ MATLAB and OriginPro were used to analyse GIXD data.

## 3. Results and discussion

### 3.1. Isotherms

**Fig. 1:**
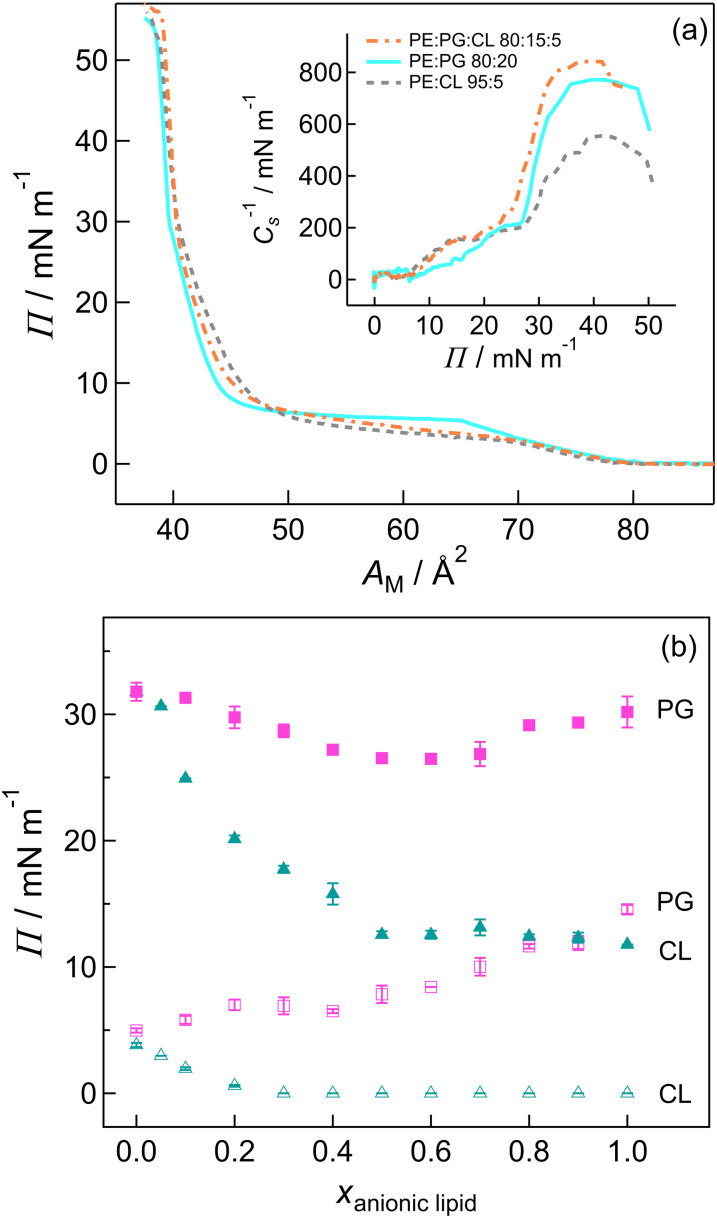
(a) Surface pressure-area isotherms of PE : PG 80 : 20 (solid cyan line), PE : CL 95 : 5 (dashed grey line) and PE : PG : CL 80 : 15 : 5 (dash-dotted orange line). Barrier speed 25 cm^2^ min^−1^, temperature 19.5 °C. The inset shows the corresponding compressibility moduli. (b) L_c_–S (filled shapes) and L_e_–L_c_ (open shapes) phase transition pressures *vs*. mol fraction anionic lipid. Pink squares PG, teal triangles CL.

Surface pressure-area isotherms were recorded for binary mixtures of PE and CL, PE and PG, and PG and CL, with mole fractions in increments of 0.1. Additional measurements were made for PG : CL 75 : 25, PE : CL 95 : 5 and PE : PG : CL 80 : 15 : 5. Fig. 1(a) compares isotherms of the ternary mixture with PE : CL 95 : 5 and PE : PG 80 : 20 and the remaining isotherms are provided in the SI (Fig. S1). The three mixtures have broadly similar phase behaviour, with an L_e_ phase at larger molecular area, an L_c_ phase and a solid phase around 40 Å^2^. The PE:PG mixture has a plateau corresponding to the condensation of the L_e_ phase into the L_c_ phase, while the monolayers containing CL show a more sloped co-existence region, with the onset of condensation at lower surface pressure than for PE:PG and the turn into the L_c_ phase at similar surface pressure. Of the three isotherms, that of the ternary mixture has intermediate gradient in the L_c_ phase and steepest gradient in the solid phase. The gradient is related to the compressibility of the monolayer. This quantity is more commonly represented as the compressibility modulus, *C*_s_^−1^, which is given by eqn (1):^90^1
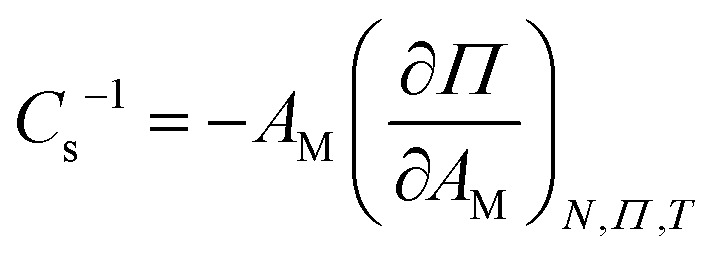
where *A*_M_ is the molecular area, *Π* is the surface pressure, *N* is the number of molecules and *T* is the temperature. A higher compressibility modulus represents a stiffer monolayer. The compressibility moduli of the three isotherms are plotted in the inset to Fig. 1(a). The PE:PG mixture has similar *C*_s_^−1^ to the ternary mixture in the solid phase, while that of the PE:CL mixture is lower. There is no strong trend in *C*_s_^−1^ with composition in the binary mixtures, although the pressure at which the maximum value occurs is lower for CL-rich mixtures than for other compositions, which reflects the fact that CL-rich monolayers form the solid phase at lower surface pressures.

The binary mixtures exhibit different trends in molecular area and phase transition pressure with composition. As expected, increasing CL content increases average molecular area because CL molecules have four tails. It also results in a decrease in pressure for both phase transitions. The dependence of phase transition pressure on composition is presented in Fig. 1(b) for PE:PG and PE:CL mixtures and is plotted as a function of mole fraction of anionic lipid. The onset of the L_e_–L_c_ phase transition pressure for PE:PG and PG:CL (see Fig. S2) mixtures increases with PG content and the onset of this phase transition for PE:CL and PG:CL mixtures decreases with CL content, as was observed by Perczyk *et al*. for tris-saline (tris + NaCl + CaCl_2_) subphases.^91^ The PE:CL behaviour closely resembles that of PE:PS mixtures: PS also has a lower phase transition pressure than PE with intermediate values for the mixtures.^77^ Evidently, more work is required to initiate condensation of monolayers containing a high proportion of PG molecules but not CL or PS molecules. There is a change in slope at roughly 40 mol% PG and between 20–30 mol% CL, which is roughly 33–40% CL chains. The dependence for PE:PS mixtures also changed at around 30–40 mol% PS, although more subtly.^77^

The trends in the L_c_–S phase transition are different. For PE:PG monolayers, the pressure at which this transition occurs passes through a minimum value between 40–70% PG, while for PE:CL monolayers, the pressure decreases as CL content increases until a minimum value is reached for 50% CL and above. PG:CL mixtures have similar behaviour to PE:PG mixtures (shown in the SI, Fig. S2). PE:PS mixtures have intermediate behaviour: a decrease in pressure is observed with increasing PS content but the slope changes at 40% PS.^77^ The results show that CL (and PS) increase the stability of the solid phase in mixtures. PG mixtures have more stable solid phases at roughly 50%, which may indicate a particular ordering of headgroups where anionic headgroups are evenly distributed, as suggested by Wydro *et al*. for DPPE and DPPG mixtures.^92^ Although PS and CL are also anionic, it is possible that the shape of the CL molecules leads to stronger inter-chain interactions that offset repulsions or that force headgroups into an arrangement where their charges are more effectively screened by water molecules. In the case of PS, it has been suggested previously that surface potential of PS in monolayers on water or on mercury is relatively low, which in the latter case was explained by a headgroup conformation that buries the charge.^93^

### 3.2. Electrochemistry

**Fig. 2:**
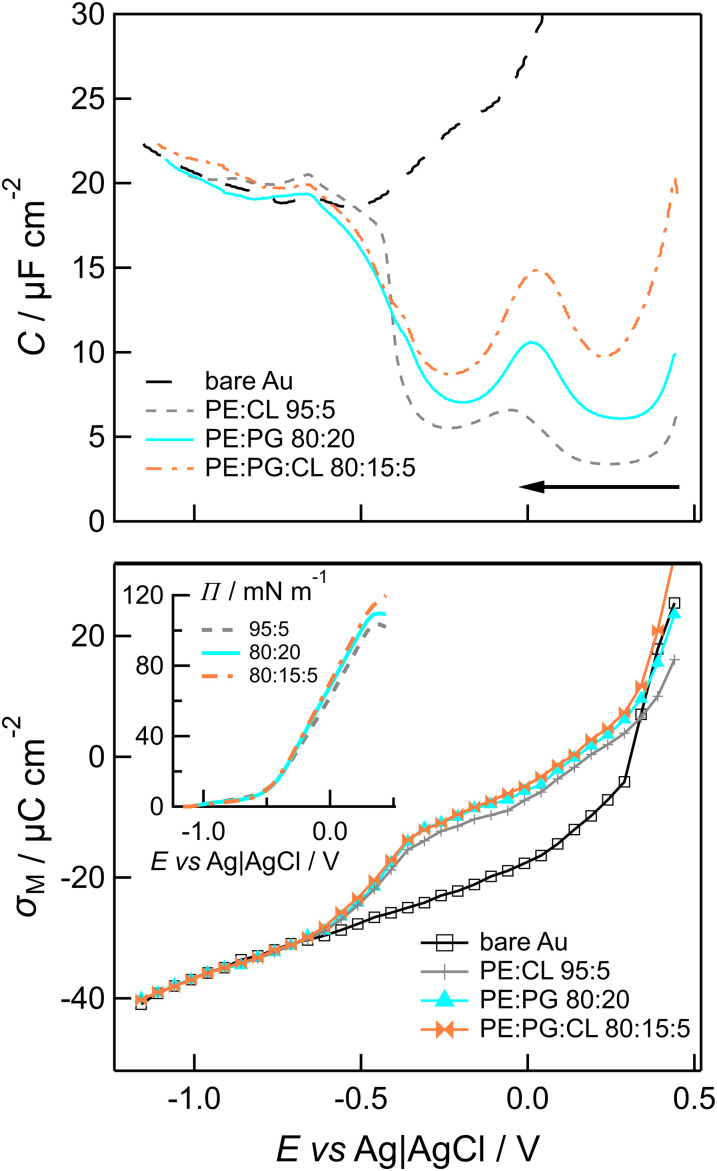
Top: Differential capacitance curves for Au(111) coated with lipid bilayers in 0.1 M NaF. Long dashed line Au, grey short dashed line PE : CL 95 : 5, cyan solid line PE : PG 80 : 20, orange dash-dotted line PE : PG : CL 80 : 15 : 5. Potential sweep rate 5 mV s^−1^ in the negative-going direction (indicated by the arrow), AC frequency 20 Hz, amplitude 5 mV. Bottom: Charge density *vs*. potential plots for Au(111) coated with lipid bilayers in 0.1 M NaF. Open shapes Au, grey crosses PE : CL 95 : 5, cyan triangles PE : PG 80 : 20, orange bowties PE : PG : CL 80 : 15 : 5. The inset shows the surface pressure *vs*. potential plots calculated from the charge density-potential plots.

Fig. 2 compares differential capacitance curves in the negative scan direction for PE : PG 80 : 20, PE : CL95 : 5 and PE : PG : CL 80 : 15 : 5 bilayers on Au(111) in 0.1 M NaF solution. The features are similar to those of previously reported lipid bilayers: the relatively low capacitance at potentials close to the potential of zero change (pzc) indicates the molecules are adsorbed on the surface and the rise in capacitance at *ca* −0.4 V indicates detachment of the bilayer from the surface. The lipids are fully desorbed from the surface at the negative potential limit. In the positive-going scan (Fig. S4), a peak corresponding to the initial adsorption is observed at around −0.9 V, followed by a peak or step at around −0.25 V to the lower capacitance values characteristic of the adsorbed bilayer. By analogy with previously reported systems,^50,68^ the step probably corresponds to a change in the state of the film where water moves in and out of the bilayer. The hysteresis between the negative-going and positive-going scans may be related to kinetics of the processes.

The minimum values of capacitance provide information on the state of the bilayers. A low capacitance, such as that exhibited by a DMPE bilayer or by the PE : CL 95 : 5 bilayer, indicates that a bilayer is thick or has low average dielectric constant or both. The latter indicates that a bilayer has a relatively low solvent content and that the surface has a high coverage with the bilayer. Typically, the bilayers with an appreciable anionic lipid content have higher specific capacitance than those with low anionic lipid content. Since the lipids all have the same tail length, the increase in capacitance is more likely to be related to greater solvation of anionic headgroups (or poorer coverage of the surface) than to differences in thickness between bilayers. For PS, this was demonstrated *via in situ* infrared spectroscopy;^94^ below we show that CL bilayers also contain a relatively high solvent content. In the case of PG and PG-rich mixtures, the high values are more likely related to poor coverage of the surface with PG. Juhaniewicz and Sek used AFM and impedance measurements to compare bilayers of DMPS and DMPG.^95^ The DMPS-coated surface was smooth with a few uncoated patches, while the DMPG bilayer was comparatively irregular and formed of small domains. The DMPG bilayers were penetrated more quickly by the small antimicrobial peptide melittin, an observation which was attributed to differences in packing density and resulting mechanical properties.^95^ Abbasi *et al*. observed corrugation and defects in floating bilayers formed from DMPC : DMPG 1 : 1 vesicles. This was explained as bilayer stress resulting from tightly packed lipids, which was alleviated on penetration by alamethicin, a small, pore-forming peptide.^96^ The irregular topography and greater number of defects observed in these studies may explain the greater solvation and lower surface pressure in our PG-rich mixtures. Abbasi *et al*. subsequently opted to use unsaturated PG lipids in electrochemical studies to increase fluidity,^97^ while other groups have opted to use DMPE as the first monolayer on which different PG bilayer mixtures can be compared.^98^ As the properties of the bilayer can be influenced by the asymmetry and order in which layers are deposited,^99^ we restricted the present study to symmetric bilayers.

Chronocoulometry data for Au(111) with bilayers of the different lipid compositions are compared in Fig. 2. The figure shows that Au coated in PE : PG 80 : 20 has similar charge density to Au coated in the ternary mixture, while the PE : CL 95 : 5 bilayer has slightly higher charge density but lower slope. Curves for the remaining mixtures are presented in Fig. S6 and S7 in the SI. As the PG content is increased beyond 20 mol% in PE or CL, the curves indicate a higher magnitude of the charge density, moving closer to the curve measured for the bare Au surface. The mixtures of PE with PG and of PE with CL have similar electrochemical properties at 10 mol% and 20 mol%. The surface pressure, *Π*, of the bilayer is given by eqn (2):^78,100^2
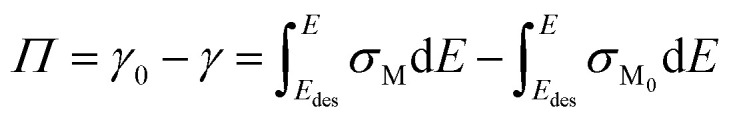
where *γ* is the surface tension, *γ*_0_ is the surface tension in the absence of the bilayer, *σ*_M_ is the charge density of the metal, *σ*_M_0__ is the charge density of the metal in the absence of the bilayer and *E* is the potential. *Π* thus equates to the area between the curves in the presence and absence of the bilayer. *Π* is plotted *vs*. potential in the inset to each panel in Fig. 2 and Fig. S6 and S7. The surface pressure falls with increasing PG content, which would suggest a relatively poor coverage of the Au surface, while the 10% and 20% PG mixtures have similar surface pressure to PE, which suggests good coverage of the surface. The maximum values occur close to the pzc and are reasonably close to twice the pressure of a monolayer. A similar trend is seen for PG:CL mixtures: increasing PG content decreases the quality of the bilayer. The PE:CL mixtures studied give good coverage and have similar electrochemical response, as was previously seen for PE:PS mixtures.^77^ The ternary mixture has similar response to the PE : CL 95 : 5 and PE : PG 80 : 20 bilayers, despite the apparently higher capacitance observed in differential capacitance experiments. Chronocoulometry measures thermodynamic properties and so provides a better representation of the equilibrium state of the bilayer for a given potential; the derivatives of the charge density-potential plots, which represent capacitance in the limit of zero frequency, are very similar.

All of the bilayers with good coverage are adsorbed within a charge density range of approx. ±10 μC cm^−2^, which is typical for phospholipid bilayers on Au(111) and corresponds to a similar range of electric field strength within which membranes in nature are stable.^50,68^ The pzc is shifted in the presence of a bilayer to more negative potentials. For zwitterionic molecules, this has been attributed to a small dipole moment caused by a charge asymmetry across the bilayer.^59^ A larger shift would normally be expected for bilayers containing anionic molecules^101^ but the results for PE:PG and PE:CL are consistent with previous studies of PE:PS bilayers, which also showed a shift in pzc for PS-rich bilayers only a little larger than for PE-rich bilayers.^77^ This result suggests the anionic lipids may be co-adsorbed with sodium counter-ions. For PE:PS, a change in pzc was observed around 30–50 mol% PS. This effect was not investigated over the whole composition range in the present work because of the poor coverage of PG-rich bilayers; however, the 5%, 10% and 20% CL samples have similar potentials of zero charge to PE:PS samples of up to 30 mol% and the 10% and 20% PG samples have more negative values (SI Table S1). The pzc for the ternary mixture (0.089 V) was closest to that of the 20% PG mixture (0.104 V). The other value of note is for pure CL bilayers, with a shift in pzc closer to equimolar PE:PS mixtures than to PS bilayers; this result may indicate that the CL molecules carry on average a charge closer to −1 than −2 in these conditions. The charge borne by CL in dispersions and monolayers has been the subject of some discussion in the literature.^66,102,103^ A recent study suggests a continuous range of protonation state over a pH range from 3 to 8, with an average charge of −1.5 at around pH 7 in the presence of monovalent cations.^66^

### 3.3. Electrochemical infrared measurements

**Fig. 3:**
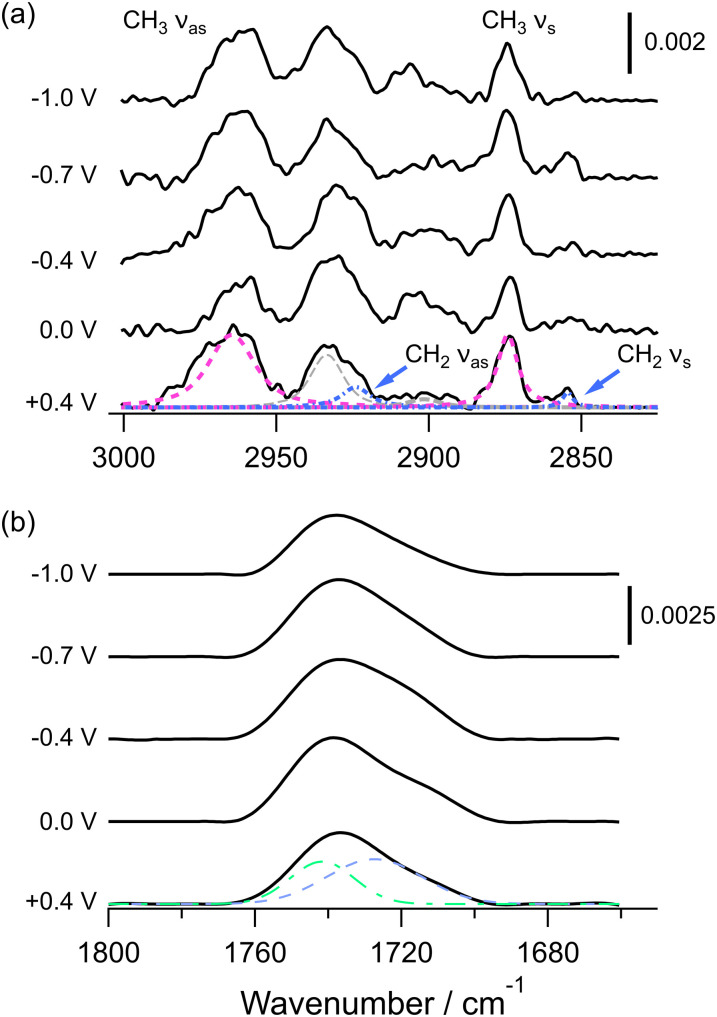
PM-IRRA spectra of TMCL bilayers on Au(111) at the indicated potentials. (a) C–H stretching region. The dashed curves are the fitted contributions of the methylene stretching vibrations (blue dash-dot), methyl stretching vibrations (pink short dash) and Fermi resonances (grey long dash). (b) C=O stretching region. The dashed curves are the fitted contributions of the hydrogen bonded (blue dash) and unsolvated (green dash-dot) carbonyl groups.

Fig. 3 presents selected PM-IRRA spectra in the C–H and C=O stretching regions of the spectrum. The C–H stretching region spectra are dominated by the symmetric and asymmetric methyl stretching modes at ∼2874 cm^−1^ and ∼2962 cm^−1^, respectively.^104,105^ The peaks corresponding to the methylene stretching modes are comparatively small and are located at ∼2853 cm^−1^ and 2923 cm^−1^.^104,105^ The other peaks arise from Fermi resonances between the overtones of the CH_2_ scissoring mode and the methylene and methyl symmetric stretching modes.^106,107^ The wavenumber of the CH_2_ modes indicates some disorder in the hydrocarbon chains^43,104,105^ but the peaks are narrow (full width at half maximum [FWHM] ∼8.5 cm^−1^ for the symmetric and ∼10 cm^−1^ for the asymmetric stretch), which indicates low mobility of the chains. The small size of the peaks suggests the transition dipole moments are oriented close to perpendicular to the surface normal, indicating the tilt of the chains is small. The tilt angles of the two modes can be estimated by comparing the integrated absorbance with the theoretical value for a randomly oriented sample.^80,81^ (These calculations are explained in the SI) The hydrocarbon chain tilt angles are estimated as ∼8° from the surface normal and do not vary strongly with the applied potential. Similar behaviour was observed for DMPE bilayers on Au(111) and results from close packing and reduced mobility of the molecules.^79^ Stacked TMCL bilayers exist in three different phases, depending on the temperature.^43^ An 
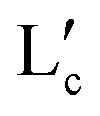
 phase with slightly tilted (∼12°) chains packed in an orthorhombic subcell is formed at low temperatures, with a phase change to an L_β_ phase consisting of untilted chains in a hexagonal arrangement and a second phase transition to an L_α_ phase, which is relatively disordered and formed of thinner bilayers. The phase transitions depend on the medium (
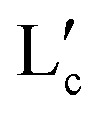
/L_β_ at 24.9 °C in tris buffer or 29.2 °C in phosphate buffer and L_β_/L_α_ at 38.9 °C in tris buffer or 41.2 °C in phosphate buffer) and show hysteresis between heating and cooling.^43^ Our experiments were performed at 19 °C but the wavenumbers do not indicate an 
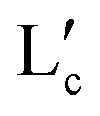
 phase. Our X-ray reflectivity measurements of the monolayers used to form bilayers indicate a hexagonal arrangement of chains (*vide infra*) so it is likely our bilayers are in an L_β_ phase but with some *gauche* conformers. Given the low mobility of the chains and the tight packing observed with diffraction (*vide infra*), it is likely that these occur around the polar/apolar region to enable the chains to align at the smaller intermolecular spacings in our monolayers. The small tilt may arise from the deposition onto the substrate, as DMPE and DMPS monolayers also contain untilted chains but form bilayers with a small tilt angle.^77,79,94^ The carbonyl stretching modes resemble those observed for the L_β_ phase, with a broad envelope comprising two peaks of similar area.^43^ The higher wavenumber peak corresponds to free ester groups and the lower wavenumber peak corresponds to ester groups participating in hydrogen bonding interactions.^43^ The proportion of the peak area corresponding to the latter is around 60%, indicating a relatively high level of solvation. For comparison, carbonyl spectra of DMPE monolayers and DMPS indicated proportions of around 30% and 40–55%, respectively,^79,94^ but the shape of the peak suggests TMCL carbonyl groups are less solvated than DMPC.^57,59^ This level of solvation explains the relatively high capacitance of the TMCL bilayers. There is a small increase in solvation around the electrochemical phase transition but the change is not significant. DMPE and DMPE-rich bilayers also show little change in solvation with applied potential.^77,79^ It is likely that the close packing of the lipids reduces flexibility to allow further solvent ingress.

### 3.4. Brewster angle microscopy

**Fig. 4:**
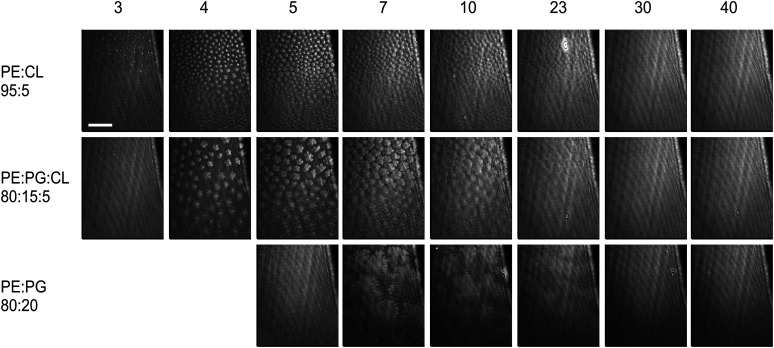
BAM images of lipid monolayers on water. The compositions are indicated at the left of the figure. The numbers indicate the pressures in mN m^−1^. The scale bar is 20 μm and the images have the same scale.

BAM was used to obtain an insight into the structure of monolayers at lower surface pressures, where diffraction is weak. Fig. 4 shows BAM images for three of the compositions at different pressures. Images for the other samples are provided in the SI (Fig. S8–S10). In PE:PG mixtures and in the ternary mixture, large, flower-shaped domains are formed during the L_e_–L_c_ phase transition. DMPG domains are smaller than the domains of DMPE and the mixtures; 50 : 50 domain sizes are smaller than 80 : 20 and 90 : 10. PE:PS equimolar mixtures also had smaller domains than the mixtures with high or low PS content.^77^ As the pressure increases, the domains begin to merge and are fully coalesced during the L_c_ phase. TMCL forms small, rounded domains at low pressures, which are coalesced by 10 mN m^−1^ (close to the kink in the isotherm). The PE:CL mixtures have tiny, round domains. Even the addition of only 5% CL into PE has a drastic effect on the condensation of the monolayer (Fig. 4). The 5% CL in the ternary mixture has a similar effect; the domains are much smaller than those of the PE : PG 80 : 20. The morphology is similar to that of the PG : CL 50 : 50 mixture, while the PG : CL 75 : 25 mixture has smaller domains. Similar small domains for these PG:CL compositions on tris-saline were observed by Perczyk *et al.*^91^ CL clearly has a very strong effect on the condensation of monolayers, as very little is required to induce significant changes in condensation. Small domains generally form where nucleation is rapid and growth relatively slow or where the line tension between the phases is high.^108,109^ A high nucleation : growth ratio is a feasible explanation: CL has strong intermolecular interactions (see diffraction data below) and can be expected to move more slowly than PE or PG at a given pressure, because of its significantly larger molecular weight. However, some of the CL mixtures have smaller domains than the pure CL monolayer so it is likely that line tension also plays a rôle. It has also been reported that CL induces curvature in membranes; for example, increasing CL content in POPE:POPG:CL vesicles decreases the radius of the vesicles whilst also increasing bilayer thickness^110^ and TOCL has been shown to thicken DOPC vesicles and increase bending modulus.^111^ TMCL has a similar effect on DMPC bilayers^112^ and CL is enriched in the more curved regions of bacterial membranes.^52^ This tendency to increase curvature may explain the apparently higher line tension in CL-containing domains.

### 3.5. Grazing incidence X-ray diffraction

**Fig. 5:**
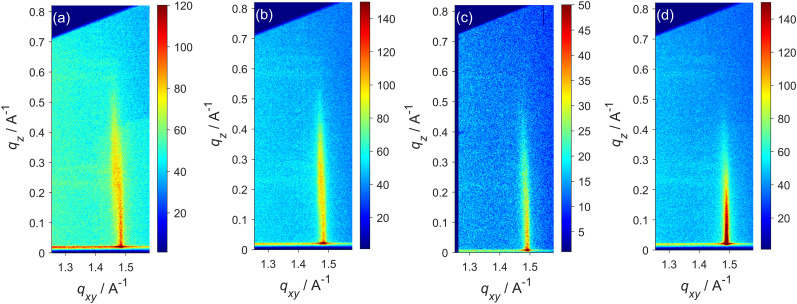
GIXD images measured at 23 mN m^−1^ for (a) DMPE, (b) DMPE : DMPG 80 : 20, (c) DMPE : TMCL 95 : 5, (d) DMPE : DMPG : TMCL 80 : 15 : 5.

GIXD was used to compare the packing arrangements of molecules within monolayers of selected mixtures and their average molecular areas. For monolayers in the solid phase, one peak is observed, typical of hexagonal close-packing of hydrocarbon tails, while in the L_c_ phase, the tilt of the tails distorts the hexagonal structure and results in splitting of the peak into two or three peaks, depending on the direction of the tilt. Example images for four of the monolayers at 23 mN m^−1^ are shown in Fig. 5. The image for the DMPE monolayer (Fig. 5(a)) clearly shows two peaks, as previously reported.^77^ The {1̄1} peak at higher *q*_*xy*_ is located at *q*_*z*_ = 0 Å^−1^; the {1 0, 0 1} peak at lower *q*_*xy*_ is located at *q*_*z*_ > 0 Å^−1^. This pattern is consistent with a distorted hexagonal structure where the tails tilt in the nearest neighbour direction. Three peaks are observed for DMPG monolayers, consistent with an oblique unit cell, where tails tilt at an angle to the nearest neighbour direction and all three inter-plane spacings differ. The mixtures of DMPE and DMPG give two peaks, closer together and at lower *q*_*z*,{10, 01}_ value than for DMPE, indicating smaller molecular footprint and tilt angle in these mixtures. Dipalmitoyl PE:PG mixtures on water (at 32.5 mN m^−1^) also show closer peaks in mixtures, close enough together to appear as one but containing components at different *q*_*z*_ positions.^92^ Comparing Fig. 5(c) and (d) with Fig. 5(a) and (b) shows a strong ordering effect of TMCL on monolayer structure: the peaks are closer together and difficult to separate. When TMCL is mixed with DMPE or DMPG at 20% or more, only one peak is observed at 23 mN m^−1^, although the peak for DMPG:TMCL 75:25 is slightly distorted. Since these measurements were made, Perczyk *et al*. reported GIXD for various DMPE:DMPG:TMCL mixtures at 25 mN m^−1^ and observed one peak in most cases.^91^ Their measurements were recorded at 20 °C on a tris-saline subphase (containing tris, NaCl and CaCl_2_), whereas ours were recorded at 19.5 °C on water and at slightly lower pressure. Any differences between Perczyk's results and ours are likely to result from the slightly lower pressure in our study and the different interactions between lipids and subphase (most likely between lipids and calcium ions in Perczyk's study). Our results at 23 mN m^−1^ are consistent with our isotherms: where the L_c_–S phase transition pressure is below 23 mN m^−1^, one peak is observed, and where it is above 23 mN m^−1^, two peaks are observed. Our measurements have focused on smaller concentrations of CL and it is noteworthy that even amounts as low as 5 mol% CL can have measurable effects on the packing arrangement of PE and PE:PG mixtures.

At 10 mN m^−1^, the DMPE mixtures all exhibit three peaks, corresponding to the oblique unit cell. The tilt angles are larger than those at 23 mN m^−1^ and the corresponding molecular footprints are larger. DMPG gives no diffraction because it is in the L_e_ phase at this pressure. TMCL and its mixtures with DMPG are in the L_c_ phase and two peaks are observed in each case, indicating tilt of the tails along the nearest neighbour direction. At the lowest pressure investigated, 6.6 mN m^−1^, not all mixtures were ordered enough to give diffraction: DMPE, DMPE : DMPG 80 : 20 and DMPG : TMCL 75 : 25 produced only a faint {1̄1} reflection. Three reflections were observed for the other mixtures (DMPG was not measured). This further highlights the ordering effects of CL and disordering effects of PG.

**Fig. 6:**
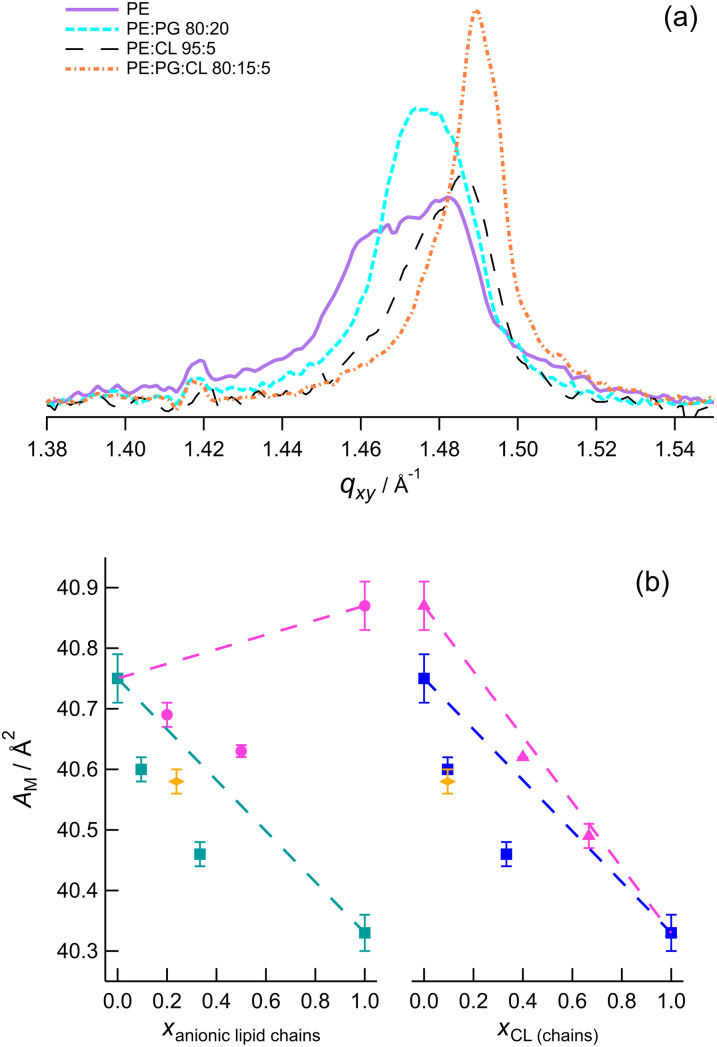
(a) Comparison of the Bragg peaks derived from the images in Fig. 5. *Π* = 23 mN m^−1^. (b) Left: Area per molecule as a function of anionic lipid content in DMPE (pink circles PE:PG, teal triangles PE:CL). Right: Area per molecule as a function of CL content (blue circles PE:CL, pink triangles PG:CL). The orange open shapes are the values for the ternary mixture. *Π* = 40 mN m^−1^. The dashed lines indicate areas expected for ideal mixing.

The intensities at each value of *q*_*xy*_ were integrated over the range of *q*_*z*_ to give plots of the Bragg peaks, which were then fitted with a Voigt function to obtain peak positions (*e.g.*Fig. 6a). The peak positions are related to the inter-plane spacings through *q*_*hk*_ = 2π/*d*_*hk*_ and can be used to obtain the average area occupied per chain (see SI). The *q*_*z*_ positions were obtained from slices of the images and used to calculate tail tilt angles as described previously.^77^ Plots of the peaks for different monolayers are given in the SI (Fig. S12–S15), along with tabulated values of the structural parameters at each pressure. A summary of the molecular areas and tilt angles is given in Table 1.

**Table 1:** Areas and tilt angles for the different lipid compositions. The areas are given per pair of chains. *t* is the tilt from the surface normal. The in-plane angles are given in the SI. The values in brackets are the errors calculated from the standard deviations in the replicate measurements

	*A*_M_/Å^2^ 40 mN m^−1^	*A*_M_/Å^2^ 23 mN m^−1^	*t*/° 23 mN m^−1^	*A*_M_/Å^2^ 10 mN m^−1^	*t*/° 10 mN m^−1^	*A*_M_/Å^2^ 6.6 mN m^−1^	*t*/° 6.6 mN m^−1^
DMPE	40.75 (0.04)	42.17 (0.09)	11.9 (0.5)	43.86 (0.20)	18.5 (1.5)	One faint peak	—
DMPG	40.87 (0.04)	42.47 (0.15)	12.7 (0.6)	—	—	—	—
TMCL	40.33 (0.03)	40.69 (0.03)	0	41.45 (0.0003)	9.3 (1.1)	41.89 (0.07)	10.7 (0.2)
PE : PG 80 : 20	40.69 (0.02)	41.82 (0.01)	10.8 (0.1)	43.64 (0.11)	15.6 (0.1)	One faint peak	—
PE : PG 50 : 50	40.63 (0.01)	41.45 (0.11)	7.6 (0.2)	43.11 (0.06)	14.7 (1.0)	One faint peak	—
PE : CL 95 : 5	40.60 (0.02)	41.60 (0.08)	8.8 (0.4)	42.94 (0.09)	15.3 (0.7)	43.42 (0.13)	19.7 (0.1)
PE : CL 80 : 20	40.46 (0.02)	40.84 (0.02)	0	42.15 (0.12)	11.5 (0.2)	42.35 (0.20)	12.3 (0.2)
PG : CL 75 : 25	40.62 (0)	40.95 (0.02)	0	42.22 (0.06)	11.6 (0.3)	One faint peak	—
PG : CL 50 : 50	40.49 (0.0182)	40.86 (0.02)	0	41.90 (0.03)	11.0 (0)	42.21 (0.18)	11.5 (0.4)
PE : PG : CL 80 : 15 : 5	40.58 (0.02)	41.23 (0.04)	5.1 (0.5)	42.67 (0.12)	13.8 (0.2)	43.26 (0.15)	14.4 (1.7)

It is clear that DMPE:DMPG mixtures are more ordered and closer-packed than the component molecules at a given pressure. DMPG occupies the largest molecular area of the compositions investigated at each pressure and a larger area than DMPS-containing samples,^77^ which is likely to be the reason for the lower Young's modulus measured for DMPG bilayers by Juhaniewicz and Sek.^95^ The reported data for a DMPE : DMPG 7 : 3 mixture on tris-saline at 25 mN m^−1^ indicates slightly greater spacing than in the pure lipids.^91^ However, Wydro *et al*. reported closer packing for DPPE : DPPG 50 : 50 mixtures on water at 32.5 mN m^−1^ compared with the pure lipids and commented that the PE ammonium groups may be more strongly attracted by the negative charges of the PG headgroup, in addition to the reduction of PG–PG repulsions in mixtures.^92^ They observed solid phases for mixtures with constant molecular area over the composition range and suggested the formation of PE : PG 1 : 1 regions. Our DMPE : DMPG 50 : 50 and 80 : 20 mixtures have different molecular areas but both are smaller than for the pure lipids, more in line with the structures of DPPE:DPPG mixtures (most likely because the experiments were carried out on similar subphases). Molecular dynamics simulations of POPE : POPG 3 : 1 mixtures show that there is a preference for PE to form hydrogen bonds with PG over other PE molecules, although both interactions occur.^113^ This finding was explained by a larger number of hydrogen bond acceptors in PG. PG–PG interactions were rare, partly as a result of the lower PG content and partly because PE has a stronger hydrogen bond donor in the ammonium group.^113^ These results suggest mixing is favoured, at least in fluid bilayers, and explain the negative excess areas observed in diffraction of PE:PG mixtures on water (*e.g*. Fig. 6b).

Increasing TMCL content in DMPE or DMPG results in closer packing and lower tilt angles. Perczyk *et al*. observed similar effects for samples containing 30 mol% or more on tris-saline buffer.^91^ Our results show that addition of even 5 mol% TMCL has a measurable effect on packing over a range of surface pressures. The ternary mixture has lower area per chain than both PE : CL 95 : 5 and PE : PG 80 : 20. The excess molecular areas are all negative, suggesting that mixing is favourable. However, if the molecular area of PG:CL is plotted against the fraction of chains belonging to TMCL, the plot is close to linear, which indicates ideal mixing for PG and CL (Fig. 6b). Calorimetric and FTIR studies of TMCL:DMPE and TMCL:DMPG dispersions in the L_β_ phase (similar to our solid phase) have also indicated relatively good miscibility (although less so in the 
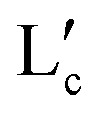
 phase at lower temperatures).^41,42^

Overall, the molecular area data show that mixing is generally favoured in the condensed phases. The A–B intermolecular interactions appear to be stronger than A–A or B–B interactions for PE-containing samples; for PG–CL in the solid phase, the mixing is close to ideal if the area is considered as a function of the mole fraction of chains (as opposed to mole fraction of molecules). Pure PE and PG and the PE : CL 95 : 5 mixture have tilted chains at 23 mN m^−1^. This is clearly related to the pressure of the L_c_–S phase transition but it is interesting to note that there is a positive correlation of GIXD molecular area with phase transition pressure, for all samples. Likewise, there is a positive correlation of the cross-sectional area of the tails with phase transition pressure (see SI). This treatment of the data appears to indicate a maximum cross-sectional area of ∼20.48 Å^2^ per chain for the L_c_ phase, which once reached induces the second-order phase transition into the solid phase. Molecules with larger average inter-chain spacings in the L_c_ phase require higher pressure to condense. These samples also have on average higher inter-chain spacings in the solid phase.

### 3.6. Reflectivity

**Fig. 7:**
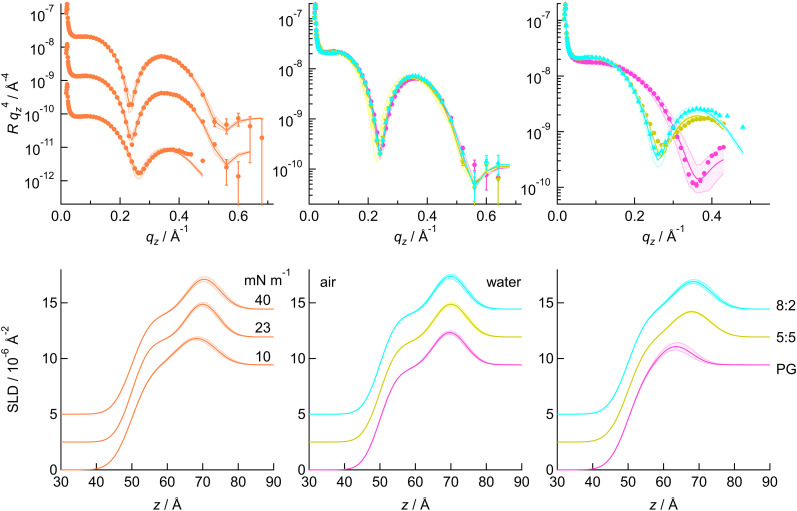
Left: XRR data for the PE : PG : CL 80 : 15 : 5 mixture on water with the corresponding SLD profiles below. Middle and right: XRR data and corresponding profiles at 23 and 10 mN m^−1^, respectively, for the PE : PG 80 : 20 mixture (cyan triangles), PE : PG 50 : 50 mixture (yellow squares) and PG (pink circles). The lines are fits to the data and the shaded regions are 95% confidence ranges. The SLD plots and the ternary mixture XRR data have been offset for clarity.

Fig. 7 presents XRR curves for the ternary mixture at three different surface pressures, with the SLD profiles determined from the fit plotted below. The minimum in the curve shifts to higher *q*_*z*_ as the pressure decreases and the monolayer becomes thinner. Changes in the depth of the minimum and in the decay with *q*_*z*_ can also be observed; the latter is mostly likely related to the roughness. The L_c_–S phase transition takes place at ∼27 mN m^−1^, so 23 mN m^−1^ is in the L_c_ phase. It is likely that the headgroups stagger in the solid phase, increasing roughness, as was previously seen for PE:PS mixtures.^77^ At 10 mN m^−1^, BAM showed some remaining inhomogeneity in the sample (the domain boundaries are still apparent), which may explain the greater roughness at this pressure. Fig. 7 also compares the XRR of the two PE:PG mixtures with PG, at 23 mN m^−1^ and 10 mN m^−1^. There is a slight difference only between PG and the mixtures at 23 mN m^−1^, while the three samples all differ in structure at 10 mN m^−1^. The data for the solid phase and for the remaining mixtures are given in the SI. In general, the greater differences between compositions were observed at 10 mN m^−1^, although CL-rich samples were distinctive, as CL-rich monolayers are more ordered at lower pressures.

The XRR data were fitted using the method previously described for PE:PS samples.^77^ Briefly, a two-slab model was used, with one slab corresponding to headgroups and the other to tailgroups. The roughnesses between the interfaces of the slabs and bulk phases were kept the same and a series of fits was carried out for different fixed roughness and tail molecular volume. The closeness of the fit to the data and the closeness of the resulting molecular area to that determined from GIXD (for 40 and 23 mN m^−1^) or the isotherm (for 10 mN m^−1^ – some BAM images show coexistence of phases at this pressure) were taken into account in selecting the best fit. This approach was adopted to avoid assumptions about molecular volume: different headgroup volumes are reported for both cardiolipins and phosphatidylglycerols. Our approach calculates the unsolvated headgroup volume using the headgroup SLD and the solvated headgroup volume, which is taken from the headgroup slab thickness and the tailgroup slab molecular area. For ease of comparison, the CL sample parameters are reported per pair of chains.

At 40 mN m^−1^, the monolayer roughness is 4.15 or 4.20 Å for all samples, with similar tail–pair volumes between 655–670 Å^3^. For PE:PG mixtures, the headgroup thickness tends to increase with increasing PG content, which results in a small increase in the overall monolayer thickness (Table 2). As previously observed for PE:PS mixtures in the solid phase,^77^ there is no strong trend in solvation at this pressure. It is likely that water is squeezed out of the monolayer at higher pressures. The headgroup volume for PG is estimated as 312 Å^3^ (confidence range 297–323 Å^3^). Kučerka and coworkers reported PG headgroup volumes of 289 Å^3^ based on molecular dynamics simulations of POPG^114^ and 291 Å^3^ based on vibrating tube densitometry and neutral buoyancy measurements in NaCl solution^115^ (the latter based on comparing PC and PG total volumes and assuming a PC headgroup volume of 331 Å^3^). A value of 346 Å^3^ was suggested by Marsh,^116^ based on the total molecular volume of 997 Å^3^ reported by Pascher *et al*.^117^ from single crystal diffraction of DMPG. Our value lies between the two. Pascher's single crystal samples formed a phase with tilted chains and a cross-sectional area of 44 Å^2^, whereas our monolayers are forced into a more compact phase with untilted chains and a cross-sectional area ∼41 Å^2^ (40.6–41.8 Å^3^). Our total molecular volume is 980 Å^3^, which is in fair agreement with that in the crystallographic study, considering the different state in which the molecules are packed. Marsh's calculations are based on a component analysis of the contributions of different portions of the molecule to the overall volume, while our division between tail region and headgroup region is not clear-cut. The fitted headgroup slab thickness is 8.0 Å (7.7–8.3 Å), which is within error of that expected from 346 Å^3^ and 44 Å^2^. Pascher *et al*. commented that the DMPG headgroup is flexible,^117^ enabling the molecules to adopt different packing arrangements. Hence it is likely that DMPG can adopt a smaller volume if forced to do so by a higher lateral pressure. The volumes for the mixtures are slightly larger than expected for ideal mixtures, which suggests non-ideal mixing (consistent with the isotherm data) but, as with the isotherms, the differences are small and on or within the error margin from the fit.

**Table 2:** Fitted XRR parameters for different lipid compositions at 40 mN m^−1^. *σ* is roughness, *V* is molecular volume and *d* is thickness of the indicated slab, *n*_w_ is the number of water molecules per lipid and *A*_M_ is the molecular area. The CL-containing monolayer values are given per pair of chains to facilitate comparison. The numbers in brackets represent 95% confidence ranges from the fits

	*σ*/ Å (fixed)	*V*_tail_/Å^3^ (fixed)	*d*_tail_/Å	*d*_hg_/Å	*n*_w_	*V*_hg_/Å^3^	*A*_M_/Å^2^
DMPG	4.2	668	16.2 (16.1–16.3)	8.0 (7.7–8.3)	0.5 (0.5–0.6)	312 (297–323)	41.2 (40.6–41.8)
PE : PG 50 : 50	4.2	660	16.2 (16.1–16.4)	7.8 (7.5–8.2)	0.9 (0.8–1.1)	291 (273–309)	40.7 (40.2–41.1)
PE : PG 80 : 20	4.18	668	16.4 (16.2–16.5)	7.4 (7.0–7.7)	0.7 (0.6–0.7)	282 (266–297)	40.8 (40.1–41.5)
PE : CL 95 : 5	4.15	655	16.3 (15.9–16.6)	7.5 (6.6–8.4)	1.9 (1.7–2.1)	244 (204–285)	40.2 (39.0–41.3)
PE : CL 80 : 20	4.13	664	16.4 (16.2–16.7)	7.2 (6.5–7.8)	0.9 (0.4–1.3)	264 (238–285)	40.5 (39.7–41.2)
TMCL	4.2	667.5	16.5 (16.3–16.7)	6.0 (6.0–6.6)	1.3 (1.2–1.4)	204 (197–230)	40.4 (39.9–41.1)
PE : PG : CL 80 : 15 : 5	4.15	668	16.3 (16.2–16.5)	7.4 (7.0–7.8)	0.8 (0.7–0.9)	280 (262–297)	40.8 (40.1–41.5)
PG : CL 75 : 25	4.15	672	16.3 (16.1–16.6)	7.4 (6.8–7.9)	0.3 (0.2–0.5)	293 (269–309)	41.1 (40.4–41.7)
PG : CL 50 : 50	4.15	671	16.5 (16.3–16.7)	7.0 (6.5–7.5)	0.2 (0.0–0.5)	279 (262–295)	40.7 (40.1–41.3)

CL-containing samples show the opposite trend in headgroup thickness (Table 2) because the CL headgroup is smaller (per pair of chains) than the headgroups of the other lipids. Consequently, the overall monolayer thickness decreases slightly in PG:CL mixtures but is similar in PE:CL mixtures as the CL content is increased. PG:CL mixtures have lower solvent content than the pure lipids. The apparent CL headgroup volume of 409 Å^3^ (394–460 Å^3^) is smaller than reported in the literature: Boscia *et al*. estimated a value of 506 Å^3^ for TMCL, based on vibrating tube densitometer measurements of DMPC and TMCL (assuming TMCL chains occupy twice the volume of DMPC chains and a PC headgroup volume of 331 Å^3^).^112^ Pan *et al.* calculated a volume of the tetraoleoyl cardiolipin (TOCL) headgroup of 493 Å^3^ from molecular dynamics simulations and 490 Å^3^ using a scattering density profile calculation (similarly to reports for PG).^111^ Our TMCL monolayers at 40 mN m^−1^ are highly compressed compared with the dispersions commonly used for molecular volume determination. The structure is dominated by very strong inter–tail interactions and the CL headgroups likely adopt a conformation that maximises the inter–chain interactions, which results in the low slab thicknesses (and correspondingly low volumes) observed. In addition, the water associated with the phosphate groups may not show much contrast with the subphase, so the water observed is likely that associated with the carbonyl groups. The CL monolayers appear to contain more solvent than PG monolayers, which is initially surprising but the infrared data for CL bilayers supports an appreciable solvent content (*vide supra*).

At 23 mN m^−1^, the PE:PG mixtures exhibit a similar trend of increasing headgroup slab thickness and volume as seen at 40 mN m^−1^. The tailgroup slabs are a little thicker for the mixtures than for the pure lipids because of the smaller areas and lower tilt angles observed with diffraction. The solvation levels vary little with composition. The estimated headgroup volumes are similar to those in the solid phase, within the error margin. The CL-containing mixtures tend to have thicker tailgroup slabs than the other samples because the addition of CL reduces the chain tilt angle and molecular area. The headgroup slab thicknesses decrease with CL content, as was seen at higher pressure (Table S11). The PG:CL mixtures have lower solvent content than the pure lipids, as in the samples at higher pressure. The ternary mixture structural parameters are almost identical to those of the PE : PG 80 : 20 mixture at both 40 mN m^−1^ and 23 mN m^−1^. The CL headgroup in the CL monolayer is apparently slightly larger at 23 mN m^−1^. This change may be a result of greater flexibility at the lower pressure and different conformation of the headgroup/chain interface. The volume at 23 mN m^−1^ (469 Å^3^) is closer to that expected from Boscia's and Pan's reports.^111,112^ most likely because those measurements and calculations were made for samples at equilibrium packing rather than samples at a high lateral pressure and the monolayer is less tightly compressed at 23 mN m^−1^ than at 40 mN m^−1^.

**Fig. 8:**
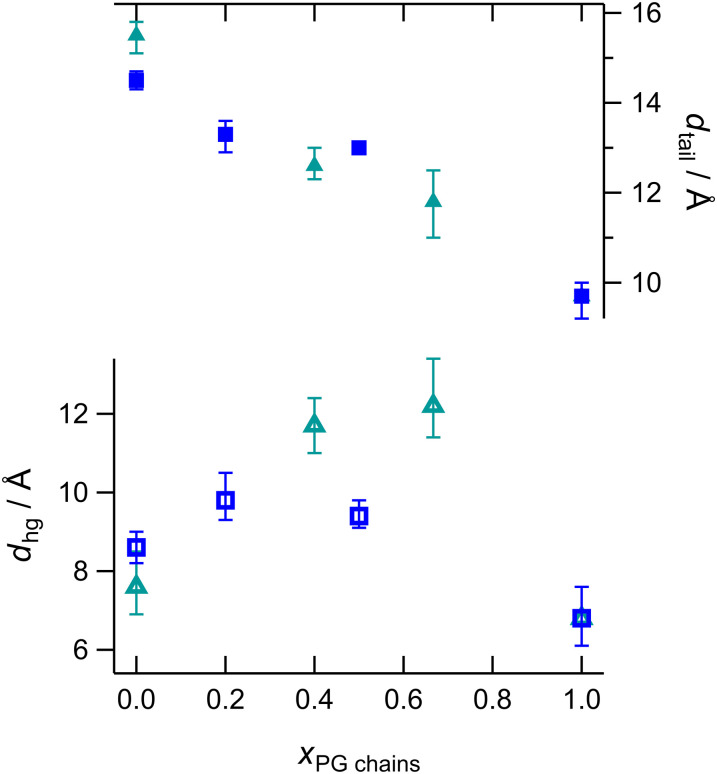
Fitted values of slab thickness at 10 mN m^−1^ (headgroups open shapes, tailgroups filled shapes) of DMPG:DMPE (blue squares) and DMPG:TMCL (teal triangles).

Larger differences between compositions are observed at 10 mN m^−1^ (Fig. 8). DMPG is in the L_e_ phase at this pressure and so the PG data fits give a larger molecular area and thinner tailgroup slab than seen with the other samples. The headgroup slab thickness is also lower, which indicates that the headgroups lie flatter when the molecules are more spaced out. The PE:PG mixtures and the ternary mixture fit to higher molecular area than expected from isotherms but the areas are consistent with the fits to neutron reflectivity data (*vide infra*). In PE:CL mixtures the 20% CL sample has thicker tailgroup slab and thinner headgroup slab, as observed at other pressures, while the 5% sample is similar to PE. In PG:CL samples, the mixtures have high water content and the headgroup volumes are close to the weighted averages. This could indicate either ideal mixing or phase separation. The 25% CL sample is not quite in the L_c_ phase at this pressure, as two regions of low slope are seen in the isotherm, suggesting a two-stage condensation or condensation of two different compositions. The 50% sample has one, sloped condensation region and is in the lower part of the L_c_ phase at 10 mN m^−1^. Both samples show distinct small condensed domains in the BAM images at these pressures. It is possible that CL nucleates condensation and PG joins the domains as the monolayer is compressed. The result is a mixed layer of average SLD in each slab. At higher pressures, the resulting phase is a non-ideal mixture. As the domains are smaller than the coherence length of the X-rays, an average SLD across the monolayer is measured so lateral variation in composition may not be discerned with reflectivity measurements alone.

**Fig. 9:**
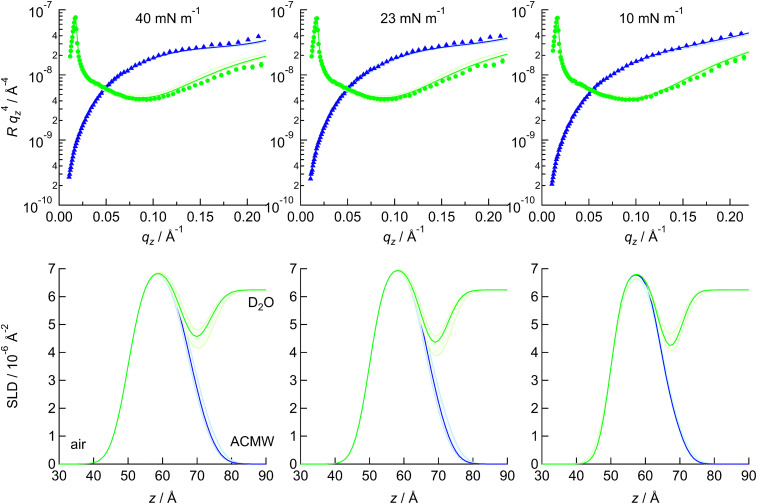
NR and corresponding SLD profiles of d-PE : d-PG : h-CL 80 : 15 : 5 at the indicated pressures. The D_2_O contrast is plotted with green circles and lines and the ACMW contrast is plotted with blue triangles and lines. The solid lines are fits to the data and the shaded regions represent the 95% confidence ranges.

NR curves and corresponding SLD plots for the ternary mixture at different pressures are presented in Fig. 9; data for the other compositions are given in the SI. As seen with XRR, the monolayers become thinner as the pressure is decreased and the solvent content increases. NR data were fitted in a similar way to XRR data but with two contrasts, which were co-fitted with common roughnesses, slab thicknesses and tailgroup SLD. The fitted parameters are given in Table 3 and Tables S13–S16. The agreement between XRR and NR data is generally very good: the molecular areas and tailgroup slab parameters are within or close to within the error of the fit, as are most of the headgroup slab thicknesses. Treatment of the DMPG headgroup slab SLD to yield unsolvated headgroup volume gives closest agreement with XRR results if both DMPG hydroxyl groups are assumed to exchange with deuterium in the solvent. The situation for mixtures with DMPE is more complex. Our previous study of DMPE suggested up to one of the ammonium hydrogen atoms was exchanged;^77^ in binary mixtures with DMPG, better agreement between headgroup volumes calculated from NR and XRR is obtained if all exchangeable atoms are assumed exchanged; however, if the total molecular volume is considered instead, it is more likely that one ammonium and two hydroxyl hydrogen atoms exchange. The data were also analysed by re-parameterising to fit area per molecule and solvation, with a series of fixed molecular volumes for tails and the headgroup molecular volumes taken from XRR fits. This treatment gave broadly similar outcomes to the original fits. For different headgroup SLD values, the closeness of the fit for PE : PG 80 : 20 was slightly better for one PE exchange than for all, while for the 50:50 sample there was little difference. Hence, we conclude it is likely that one PE and both PG hydrogen atoms are exchanged but from these two contrasts alone it is not possible to determine with a high degree of certainty. The number of water molecules per lipid is similar in each case to XRR at the higher pressures, with on average 0.5–1 water molecule at 40 mN m^−1^ and 0.5–2 water molecules at 23 mN m^−1^ but low at 10 mN m^−1^ (where the contrast may be weaker). Although these values are low compared with those reported for DPPE:DPPG mixtures (as are our molecular areas),^118^ our results are consistent with our GIXD measurements and the reported DPPE:DPPG results are consistent with the reported isotherms. The difference is most likely because our monolayers were prepared on water, while the DPPE:DPPG monolayers were prepared on a buffered subphase; monolayers containing anionic mixtures tend to occupy larger area per molecule on average when spread on electrolytes that do not contain divalent cations,^119,120^ while interactions between these lipids on water tend to be dominated by hydrogen bonding. Measurements were also made at 6.6 mN m^−1^, which is in the L_e_ phase for samples containing deuterated PG, and showed a tendency for the molecular area to increase with increasing PG content. This tendency is related to the fact the L_e_ phase begins to form at higher molecular area in PG-rich samples.

**Table 3:** Fitted NR parameters for different lipid compositions at 40 mN m^−1^. *σ* is roughness, *V* is molecular volume and *d* is thickness of the indicated slab, *n*_w_ is the number of water molecules per lipid and *A*_M_ is the molecular area. The CL-containing monolayer values are given per pair of chains to facilitate comparison. The numbers in brackets represent 95% confidence ranges from the fits

	*σ*/Å (fixed)	*V*_tail_/Å^3^ (fixed)	*d*_tail_/Å	*d*_hg_/Å	*n*_w_	*V*_hg_/Å^3^	*A*_M_/Å^2^
DMPG	3.6	670	16.1 (15.6–16.6)	7.6 (6.8–8.4)	0.0 (2 ex)	315	41.7 (40.3–43.0)
					0.5 (1 ex)	300	
					1.0 (0 ex)	284	
PE : PG 50 : 50	3.1	680	16.6 (16.1–17.2)	7.8 (6.6–8.8)	1.3 (all)	278	41.0 (39.4–42.4)
					1.9 (1PE2PG)	261	
PE : PG 80 : 20	3.2	675	16.4 (16.0–16.9)	6.2 (6.0–7.9)	0.4 (1PE2PG)	241	41.1 (39.8–42.3)
PE : CL 80 : 20	3	670	15.3 (15.0–15.9)	6.5 (6.0–7.6)	1.7 (all)	231	43.7 (42.1–44.7)
PE : PG : CL 80 : 15 : 5	4	680	16.3 (16.0–16.9)	6.0 (6.0–6.8)	0 (1PE)	256	41.7 (40.3–42.5)
					0.3 (0 PE)	243	
					PG/CL ex		

The agreement between XRR and NR for PE : CL 80 : 20 was not as good as for PE:PG mixtures but a similar trend with pressure was observed. The most plausible fit is obtained if the PE and CL exchangeable hydrogen atoms are allowed to exchange with D_2_O. The resulting number of water molecules per lipid (by pair of chains) is 2, matching that obtained from XRR, and the headgroup volume is closer to the expected value for ideal mixing, most likely because the slab thickness is lower in NR fits. This is a result of the lower contrast between headgroups and subphase in NR than XRR. Similarly, in the ternary mixture, the headgroup slab appears thinner in NR data than XRR data but comparable tailgroup slab thickness and SLD are obtained in the solid phase measurements. The ternary mixture has a thicker tail region and a thinner headgroup region than the PE:PG 80:20 sample but similar solvation levels. In the L_e_ phase, it has a smaller average molecular area. The neutron and X-ray reflectivity data thus show similar tendencies for favourable mixing between PE and PG and between PE and CL, at each pressure. Comparing the ternary mixture with the binary mixtures suggests that in the more condensed phases, adding CL at 5 mol% has less effect on PE:PG than introducing PG at 15 mol% to PE:CL, but rather the amount of the major anionic lipid is more important, which is reflected in the electrochemical data. The differences between the ternary mixture and the binary mixtures are more apparent in the GIXD results and it is possible that the subtle differences in packing or tilt do not alter the average density or thickness of a slab sufficiently to cause large differences in reflectivity data. The trends observed with GIXD do, however, provide insight into the thermodynamic phase behaviour, as the closer-packed samples tend to have lower phase transition pressures.

### 3.7. General discussion

The trends in PE:CL isotherms are similar to those we previously reported for PE:PS isotherms.^77^ Increasing PS or CL content decreases the L_e_–L_c_ phase transition pressure, with a change in slope at *ca* 30–40% (by mole of chains) of anionic lipid. Increasing PG content increases the pressure of the L_e_–L_c_ phase transition in mixtures with PE or CL. The PE L_c_–S phase transition pressure decreases with increasing PS or CL content, with a discontinuity in the slope at 30–40% or 67% (by mole of chains), respectively, which may be related to surface charge density if the CL headgroup has an average charge of –1. The values of pzc in the electrochemistry experiments support a charge of –1 on CL. PG mixtures with PE or CL show a minimum in this phase transition at 50% (by mole of chains), indicating mixing is most favourable in equimolar mixtures. The BAM and GIXD data correlate well with these trends. Addition of small amounts of CL to PE, PG and PE:PG samples decreases condensed phase domain size during the L_e_–L_c_ phase transition, even at levels as low as 5 mol%, and causes a reduction in the area occupied per chain and the chain tilt angle over a range of pressures. The closer packing in condensed phases is consistent with the stronger thermodynamic driving force for condensation observed in isotherms, which is in turn consistent with the higher nucleation rate indicated in BAM images. Average chain areas show non-ideal favourable mixing in PE:PG and PE:CL, but ideal mixing for PG:CL when considered as mole fraction of chains. There appears to be a common threshold in cross-sectional area below which the solid phase is formed. The X-ray and neutron reflectivity results show good agreement with one another. The trends in these data are partly determined by the different phases of each composition: at 10 mN m^−1^ PG is in the L_e_ phase and at 23 mN m^−1^ the samples with >20 mol% CL are in the solid phase. With the exception of samples in the L_e_ phase, the different unsolvated volumes of the headgroups lead to an increase in headgroup slab thickness with PG or PS content and a decrease with increasing CL content. The PG headgroups reorient and flatten in the L_e_ phase. Increasing CL content increases tailgroup slab thickness in both PE:CL and PG:CL, consistent with the reduction in area per chain and chain tilt angle observed with GIXD. CL appears more solvated than PE or PG at 40 mN m^−1^ and both the low tilt angle and higher solvation are also seen in the electrochemical infrared spectra. The resistance to perturbation observed in the IR spectra is unusual in lipid bilayers but is consistent with the close-packing and condensation at low surface pressure observed in the CL monolayers. Reflectivity also indicates low solvation in most of the mixtures and the trends in solvation are weak at 40 mN m^−1^ and 23 mN m^−1^. These results explain why bilayers of PE:CL and PE:PG mixtures of up to 20 mol% anionic lipid have similar electrical barrier properties and electrochemical phase behaviour. At 10 mN m^−1^, PE:PG monolayers contain more water than PE:CL because the PE:CL monolayer is more ordered (with smaller molecular area and tilt angle) than PE:PG monolayers. At the equivalent pressure in bilayers, the molecules are beginning to detach from the electrode surface, so the ingress of water into the film increases capacitance and charge density. This type of behaviour thus appears to be common to all the bilayer compositions studied.

The results have also demonstrated the value of combining information from several techniques. GIXD has provided a detailed insight into the trends in phase transitions and was invaluable in the analysis of the reflectivity data. For samples containing PG and CL, the variation of measures of the closeness of the fit (*e.g*. χ^2^) with a given structural parameter (*e.g*. tail molecular volume) was shallower than for PE, PS or PE:PS, so the independent determination of tail area with GIXD is vital for fitting the reflectivity results with confidence. The approach has allowed a determination of headgroup molecular volume that can be employed in re-parameterisation fitting strategies and in future studies including unsaturated lipids and studies of membrane penetration by candidate antibacterial agents, removing the need to assume the values of headgroup parameters. GIXD also discerned greater differences between samples than XRR, NR and electrochemistry. The electrochemical measurements give information on macroscopic properties and the reflectivity measurements provide an average lateral composition (they are sensitive to the variation in composition in the direction normal to the surface), so reflectivity is very well suited to explaining and interpreting electrochemical phase behaviour and deserves wider application in this way. Reflectivity measurements can help to determine whether capacitance variations arise from thickness or solvation differences. By studying structure of monolayers over a range of surface pressure, it is possible to explain changes in bilayers as *trans*-membrane potential is varied. IR and GIXD are more sensitive to lateral packing of molecules and ordering within the monolayer or bilayer and these techniques can explain the susceptibility of the layers to perturbation, for example by an electric field^77^ or by an external agent such as an enzyme,^91^ peptide or therapeutic molecule. Hence, it is beneficial to utilise a combination of techniques within these groups of methods to gain a full picture of composition-property relationships.

## 4. Conclusions

Bacterial, mammalian and mitochondrial membranes all contain anionic lipids but the types and amounts of these lipids vary. In this study, we compare model bacterial membranes composed of DMPE, DMPG and TMCL with our previous models of the inner plasma membrane of mammalian lipids, containing DMPE and DMPS.^77^ TMCL bilayers are tightly packed with very low tilt angle and are well solvated but are unusually resistant to perturbation by an applied electric field, which is likely to be a result of the strong chain–chain interactions and flat-lying headgroups. In mixtures, TMCL has similar effects on DMPE structure and properties to DMPS, which may indicate similar rôles in different membrane types. TMCL has a particularly strong ordering effect on both DMPE, DMPG and their mixtures, reducing molecular area and tilt angle and thickening the tailgroup region, even at very low levels (5%). DMPG on the other hand, has different effects, increasing the work required to condense a film and increasing surface charge density to a greater extent than the other lipids. The ternary PE:PG:CL mixture shows similar reflectivity and electrochemical properties to the closest PE:PG mixture. However, its condensation behaviour is more like that of the closest PE:CL mixture and it has closer packing and lower chain tilt angle than either of the binary mixtures (whilst similar to or higher than PG:CL). Hence, all three lipids influence overall structure.

These findings highlight the importance of utilising a wide range of techniques: the diffraction highlights subtle structural differences that can be important in susceptibility of membranes to perturbation, while the combination of the structural methods enables interpretation of macroscopic properties. The results also establish that the headgroup type in model membranes is important rather than the charge alone and that the ternary mixture may be better suited than binary mixtures for future studies of bacterial membrane processes and potential antibacterial agents. The subtle structural differences between these samples may determine their response to perturbation; hence to predict the susceptibility of a Gram-negative bacterial cell membrane to treatment with new therapies, the ternary mixture is recommended.

## Conflicts of interest

There are no conflicts of interest to declare.

## Supplementary Material

SM-021-D5SM00378D-s001

## Data Availability

Neutron reflectivity data are openly available from ISIS at https://doi.org/10.5286/ISIS.E.RB1900125. Other data supporting this publication are openly available from the UBIRA eData repository at https://doi.org/10.25500/edata.bham.00001252. Additional surface pressure-area isotherms and analysis of phase transition pressures, additional differential capacitance and chronocoulometry data, explanation of the PM-IRRAS analysis, additional GIXD data and analysis, additional neutron and X-ray reflectivity data and analysis. See DOI: https://doi.org/10.1039/d5sm00378d
