# Supplementary material for: Bacterial cell membrane models: choosing the lipid composition
Source: Soft Matter. 2025 Aug 26;21(36):7054–73. doi: 10.1039/d5sm00378d (PMC12379600; doi:10.1039/d5sm00378d)
Supplement: SM-021-D5SM00378D-s001 [file SM-021-D5SM00378D-s001.pdf]

Supplementary Information (SI) for Soft Matter.
This journal is © The Royal Society of Chemistry 2025

Supporting Information for
Bacterial Cell Membrane Models: Choosing the Lipid Composition

Alexandra L. Martin,[1] Philip N. Jemmett,[1] Thomas Howitt,[1] Mary H. Wood,[1†] Liam R. Cox,[1] Timothy R. Dafforn,[2] Mario Campana,[3] Rebecca J. L. Welbourn,[3,4] Maximilian W. A. Skoda,[3] Luke A. Clifton,[3] Hadeel Hussain,[5] Jonathan L. Rawle,[5] Francesco Carlà,[5] Christopher L. Nicklin,[5] Thomas Arnold[3,5,6,7]* and Sarah L. Horswell[1]*

1. School of Chemistry, University of Birmingham, Edgbaston, Birmingham, B15 2TT, UK

2. School of Biosciences, University of Birmingham, Edgbaston, Birmingham, B15 2TT, UK

3. ISIS Pulsed Neutron and Muon Source, Science and Technology Facilities Council, Rutherford Appleton Laboratory, Harwell, Oxfordshire, OX11 0QX, UK

4. Neutron Scattering Division, Oak Ridge National Laboratory, Oak Ridge, Tennessee 37831, USA

5. Diamond Light Source, Harwell Science and Innovation Campus, Chilton, Didcot, Oxfordshire, OX11 0DE, UK

6. European Spallation Source ERIC PO Box 176, SE-221 00 Lund, Sweden

7. Department of Chemistry, University of Bath, Claverton Down, Bath, BA2 7AY, UK

** Corresponding authors: tom.arnold@ess.eu, s.l.horswell@bham.ac.uk*

† Present address: Niels Bohr Institutet, Københavns Universitet, Blegdamsvej 17, DK-2100 København, Denmark

S1. Surface pressure-area isotherms

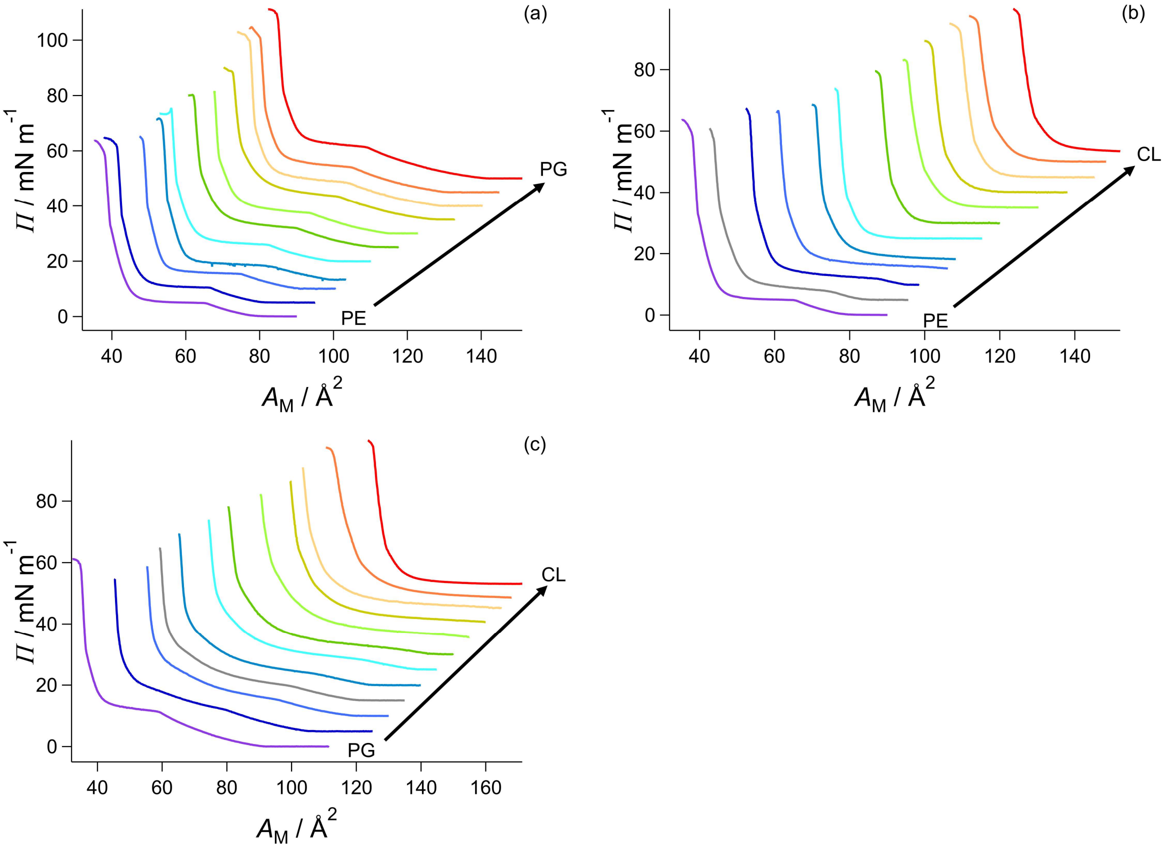

Figure S1. *Surface-pressure isotherms for various lipid mixtures on water: (a) DMPE and DMPG with x_{PG} in steps of 0.1; (b) DMPE and TMCL with x_{CL} 0.05, 0.1−0.9 in steps of 0.1; (c) DMPG and TMCL with x_{CL} 0.1, 0.2, 0.25, 0.3−0.9 in steps of 0.1. The compression rate was 25 cm^2 min^{-1} (max. trough area ~600 cm^2) and the temperature was 19.5 °C.*

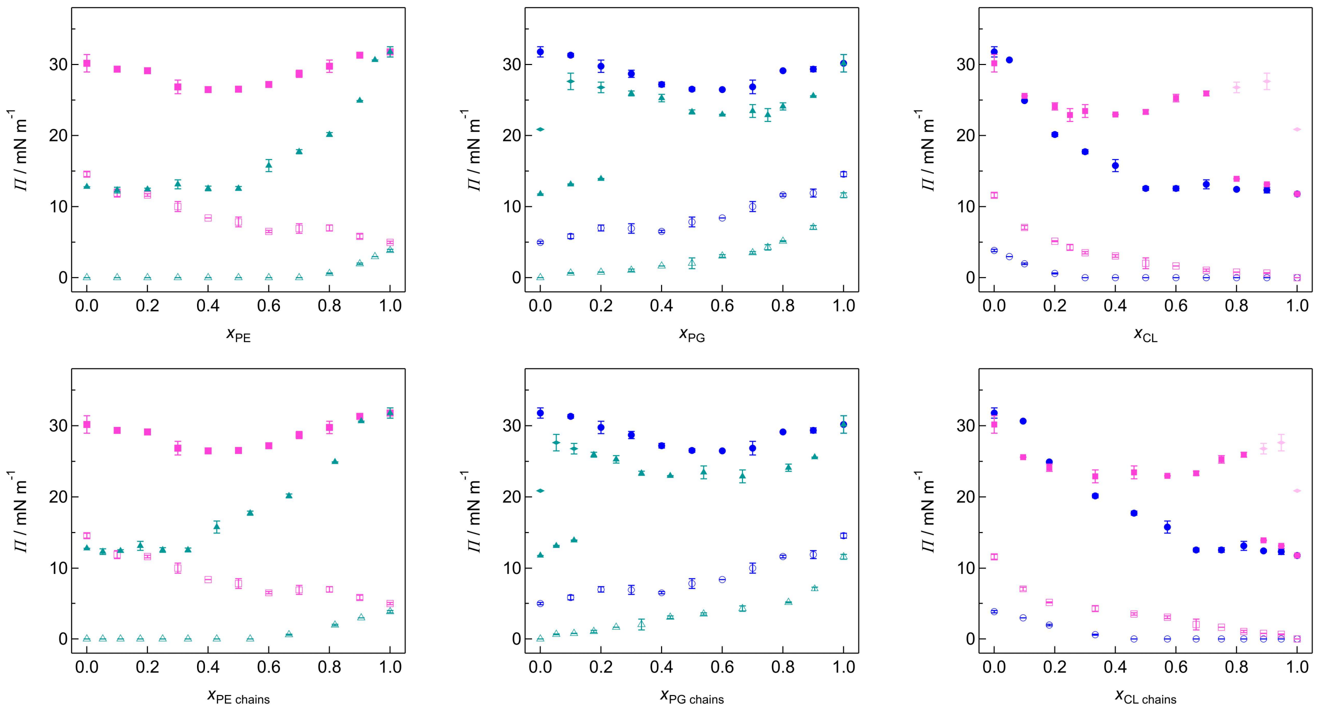

Figure S2. *L_c–S (filled shapes) and L_e–L_c (open shapes) phase transition pressures vs mole fraction of the indicated lipid (top) or vs mole fraction of chains of the indicated lipid (≈ chain vol. fraction) (bottom). Left: PE mixed with PG (pink squares) and CL (teal triangles). Middle: PG mixed with PE (blue circles) and CL (teal triangles). Right: CL mixed with PE (blue circles) and PG (pink squares). The diamond shapes for CL-containing samples correspond to an inflection in the curve where the slope appears to change shape (in addition to the obvious kink at lower pressure). The L_e–L_c transition pressure plotted are the onset; note that PG:CL samples tend to have a sloped L_e-L_c co-existence region. The PG:CL samples of low CL mole fraction (10–30%) in particular have poorly defined regions between the "plateaux" and the solid phases. The data points in these cases represent the approximate point where the phase changes but may be more properly considered as a range roughly 4 mN m^{-1} wide.*

S2. Electrochemistry

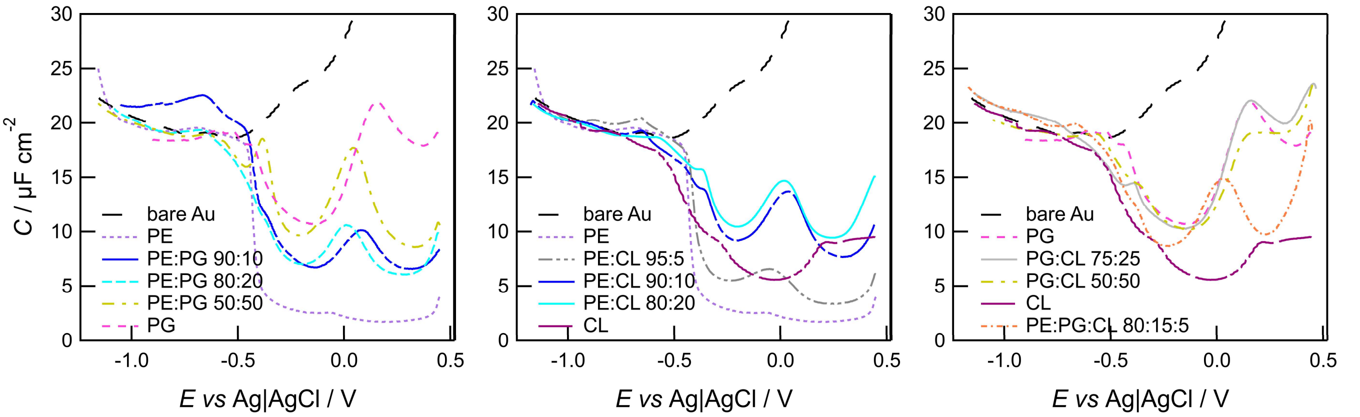

Figure S3. *Comparisons of differential capacitance curves for bilayers on Au(111) in 0.1 M NaF, recorded at a sweep rate of 5 mV s^{-1} in the negative-going sweep direction. AC frequency 20 Hz; amplitude 5 mV. (a) DMPE, PE:PG 90:10, PE:PG 80:20, PE:PG 50:50 and DMPG; (b) DMPE, PE:CL 95:5, PE:CL 90:10, PE:CL 80:20 and TMCL; (c) PG:CL 75:25, PG:CL 50:50, PE:PG:CL 80:15:5.*

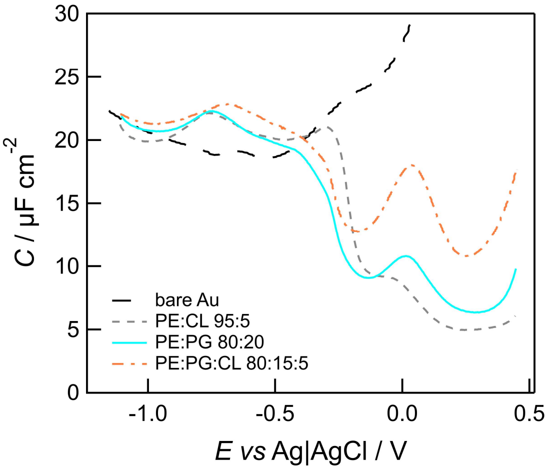

Figure S4. *Comparison of the differential capacitance in the positive-going sweep for bilayers of PE:PG 80:20, PE:CL 95:5 and PE:PG:CL 80:15:5 on Au(111) in 0.1 M NaF solution, recorded at a sweep rate of 5 mV s^{-1}. AC frequency 20 Hz; amplitude 5 mV.*

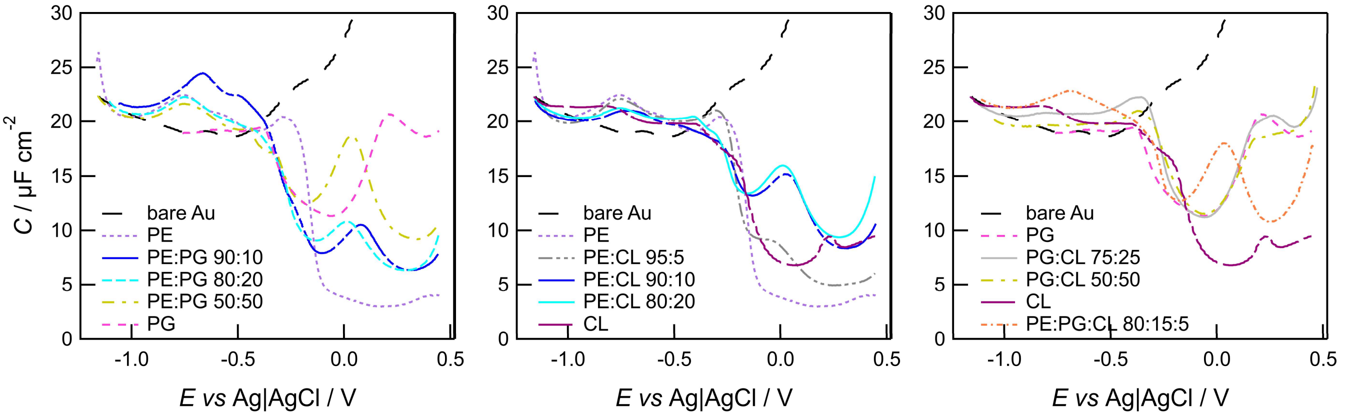

Figure S5. *Comparisons of differential capacitance curves for bilayers on Au(111) in 0.1 M NaF, recorded at a sweep rate of 5 mV s^{-1} in the positive-going sweep direction. AC frequency 20 Hz; amplitude 5 mV. (a) DMPE, PE:PG 90:10, PE:PG 80:20, PE:PG 50:50 and DMPG; (b) DMPE, PE:CL 95:5, PE:CL 90:10, PE:CL 80:20 and TMCL; (c) PG:CL 75:25, PG:CL 50:50, PE:PG:CL 80:15:5.*

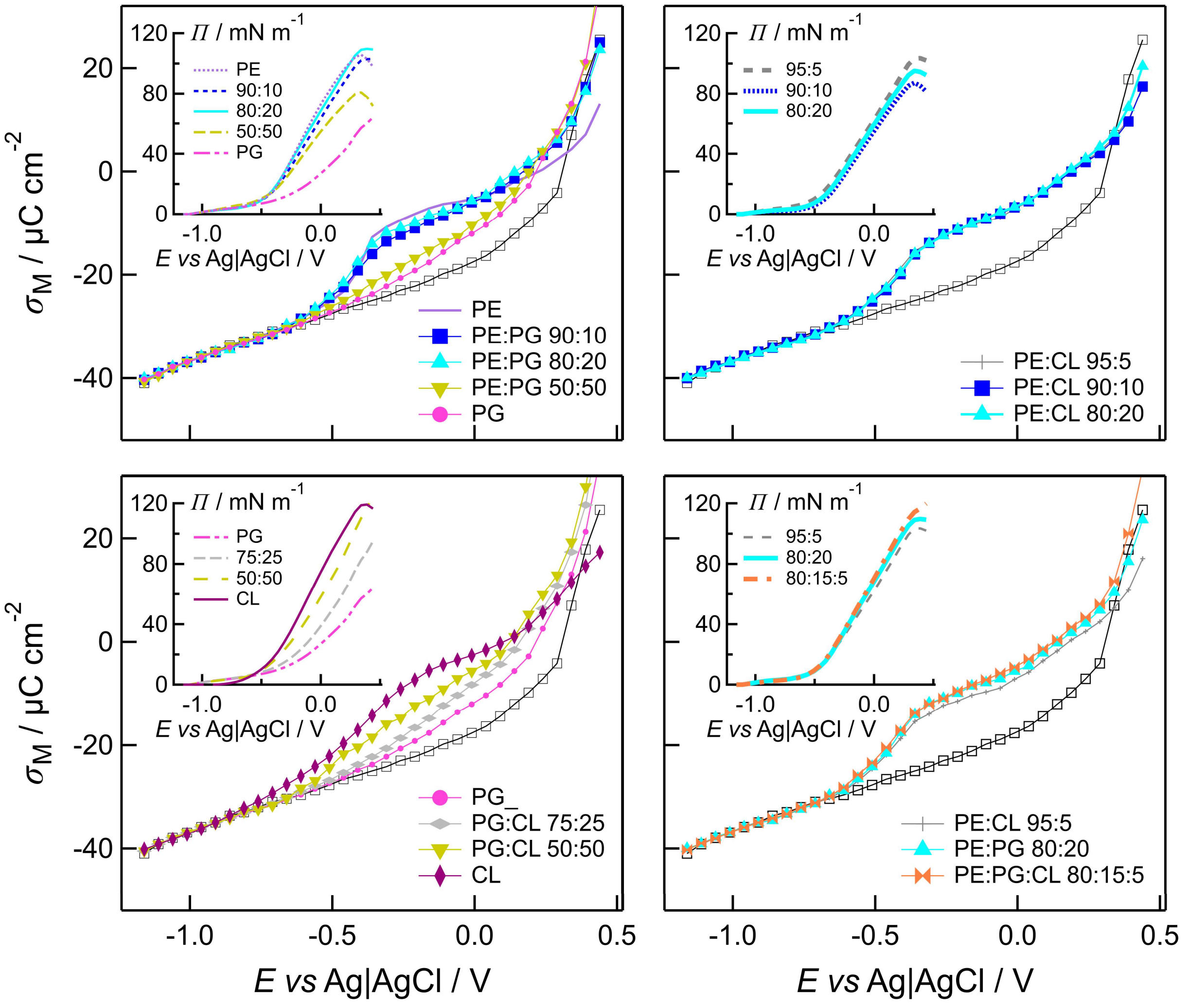

Figure S6. *Charge density vs potential plots for Au(111) coated with lipid bilayers in 0.1 M NaF. The insets show surface pressure vs potential plots calculated from the charge density-potential plots.*

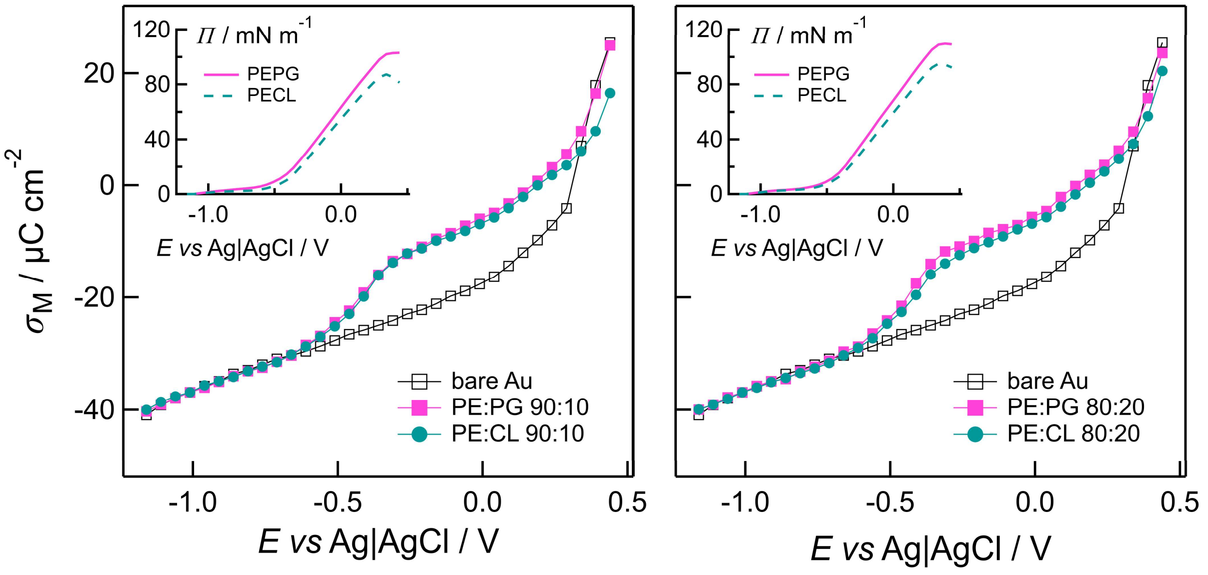

Figure S7. *Comparisons of charge density vs potential for PEPG and PECL bilayers on Au(111) in 0.1 M NaF. Left: PE:PG 90:10 pink squares, PE:CL 90:10 teal circles. Right: PE:PG 80:20 pink squares, PE:CL 80:20 teal circles. The insets show surface pressure-potential plots derived from the charge-density-potential plots. PE:PG solid pink lines, PE:CL teal dashed lines.*

Table S1. *Potentials of zero charge for Au(111) coated in the indicated bilayers.*

bilayer	PE	PE:PG 90:10	PE:PG 80:20	PE:CL 95:5	PE:CL 90:90	PE:CL 80:20	PE:PG:CL 80:15:5	CL
pzc *vs* Ag|AgCl / V	0.180	0.132	0.104	0.145	0.150	0.140	0.089	0.062

The results show a progressively negative shift for PE:PG bilayers, while the values for PE:CL bilayers are similar and within the step size of the measurements (50 mV). PE:PS mixtures also displayed a similar shift within this composition range.[S1] The ternary mixture value is most similar to the value for 20% PG. (Note that only values for samples of good coverage are reported as values for the other samples are mainly dependent on the coverage.)

S3. Polarisation Modulation Infrared Reflection Absorption Spectroscopy

The orientation of the transition dipole moment corresponding to a vibration can be calculated from the integrated peak area of the spectrum using Eq. S1.[S2,S3]

$$\int A\mathrm{d}\tilde{\nu} \propto |\boldsymbol{\mu}\cdot\boldsymbol{E}|^2 = |\mu|^2|E|^2\cos^2\theta \qquad \text{Eq. S1}$$

where A is the absorbance, $\tilde{\nu}$ is the wavenumber, $\boldsymbol{\mu}$ is the dipole moment, $\boldsymbol{E}$ is the electric field vector and θ is the angle made between the dipole moment and the electric field vector. At the Au/water interface, the electric field component parallel to the surface is close to zero, as a result of a phase shift between the incident and reflected beams. In contrast, the component of the field perpendicular to the surface is enhanced. Consequently, the direction of this vector can be considered normal to the surface, which means that θ represents the angle between the transition dipole moment and the surface normal.[S2,S3] The peak intensity is also related to the amount of material in the path of the beam, so it must be compared with that of an equivalent film of randomly oriented molecules. The spectrum of randomly oriented molecules can be computed from the isotropic optical constants of the materials (window, electrolyte, adsorbate and substrate), using the same angle of incidence and electrolyte thickness as in the experimental measurement. The optical constants of cardiolipin were obtained from transmission infrared measurements of a known concentration of vesicles in 0.1 M NaF in D_2O using a previously described method.[S2,S3] The peaks in the experimental spectrum and theoretical spectrum of randomly oriented molecules are fitted and the resulting peak areas are used to calculate the angle of the transition dipole from the surface normal with Eq. S2:[S2–S4]

$$\cos^2\theta = \frac{1}{3}\frac{\int_{\mathrm{exptl}} A\mathrm{d}\tilde{\nu}}{\int_{\mathrm{random}} A\mathrm{d}\tilde{\nu}} \qquad \text{Eq. S2}$$

The angles of the methylene asymmetric and symmetric stretching modes are mutually orthogonal and are both orthogonal to the direction made by the chain backbone. Once the tilt angles of the two vibrational modes have been calculated, Eq. S3 can be used to calculate the tilt angle of the hydrocarbon chain from the surface normal:[S5]

$$\cos^2\theta_{\mathrm{asym}} + \cos^2\theta_{\mathrm{sym}} + \cos^2\theta_{\mathrm{chain}} = 1 \qquad \text{Eq. S3}$$

where θ_{asym} and θ_{sym} are the angles between the asymmetric and symmetric stretching modes and the surface normal, respectively, and θ_{chain} is the tilt angle between the hydrocarbon chain axis and the surface normal.

The resulting chain tilt angles are within the range of 5–10°. The values at positive potentials are in general a little higher than those at negative potentials but the difference is small compared with the error margin, which is expected to be ~4°.[S4,S6]

Tilt angles were not calculated for the peaks in the carbonyl region because the peak intensity can be affected by differences in solvation as well as by the tilt angle.

S4. Brewster Angle Microscopy

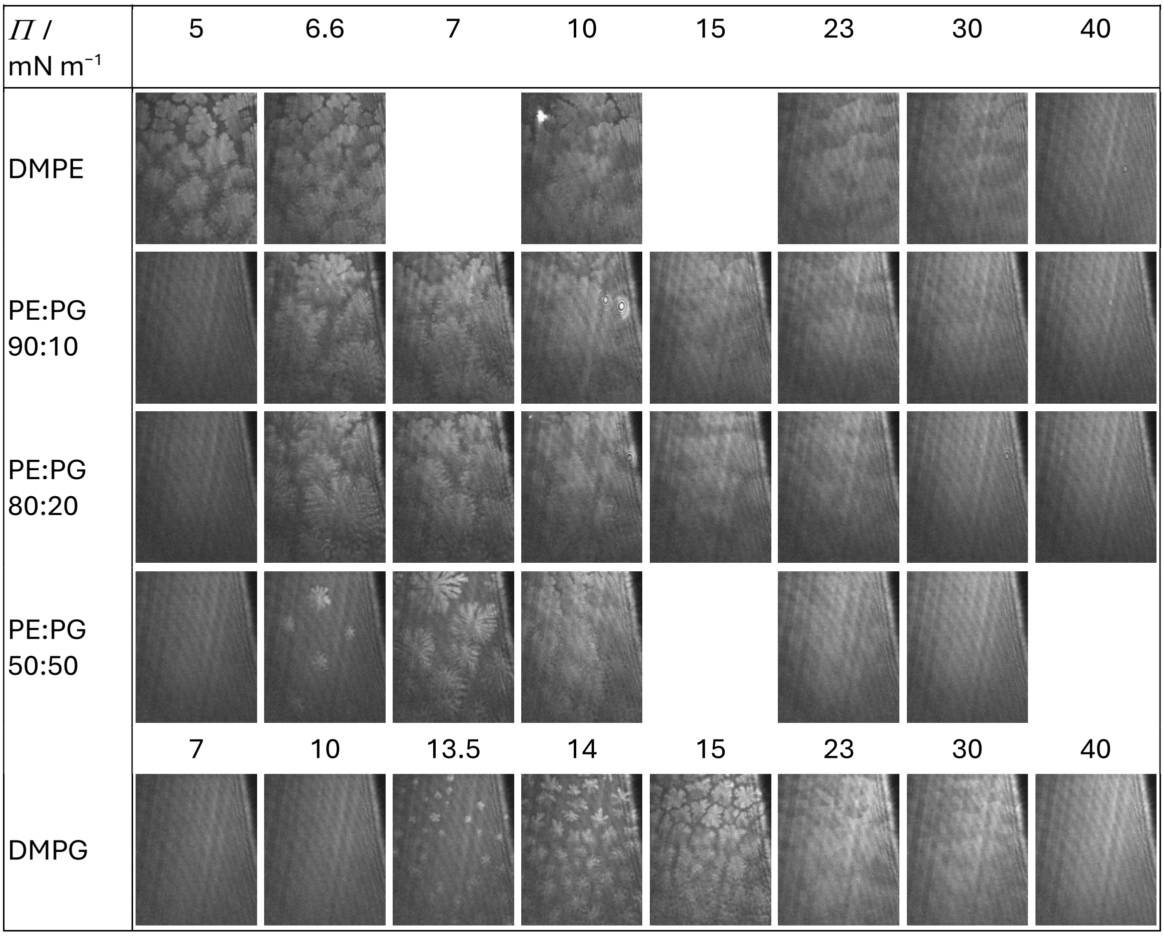

Figure S8. *BAM images of PE:PG lipid monolayers on water, with compositions indicated at the left and surface pressures in mN m^{-1} at the top. The image size is 68 μm by 85 μm.*

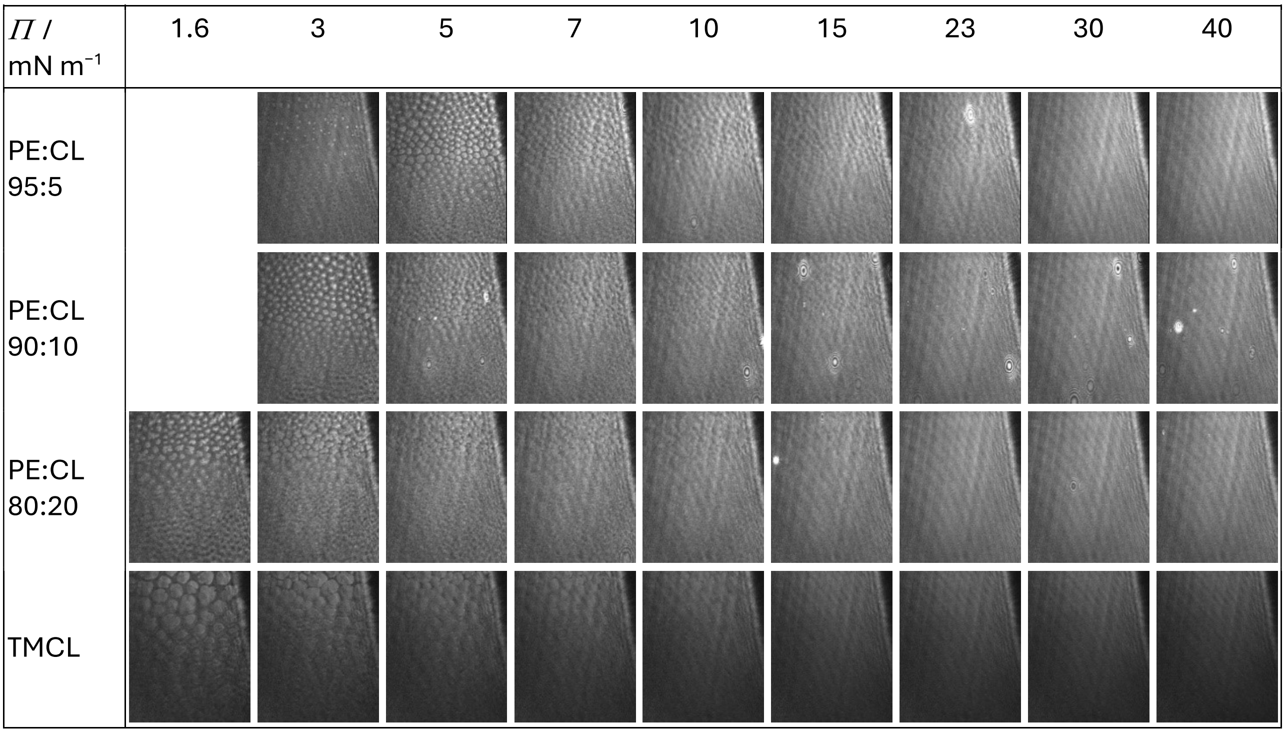

Figure S9. *BAM images of PE:CL lipid monolayers on water, with compositions indicated at the left and surface pressures in mN m^{-1} at the top. The image size is 68 μm by 85 μm.*

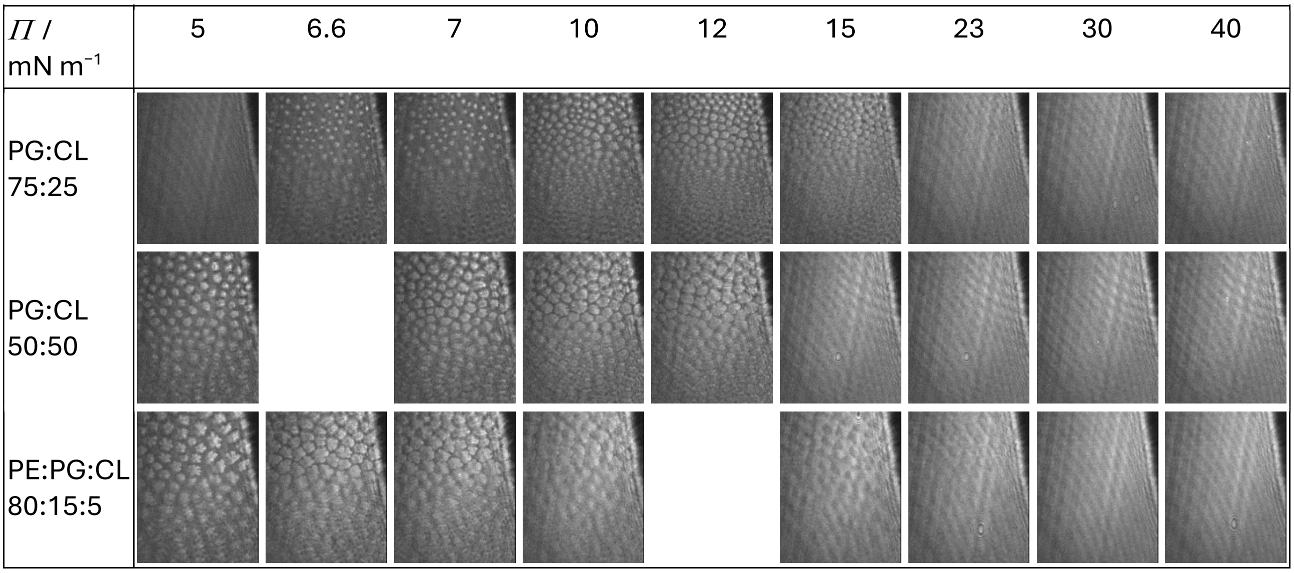

Figure S10. *BAM images of PG:CL lipid monolayers on water, with compositions indicated at the left and surface pressures in mN m^{-1} at the top. The image size is 68 µm by 85 µm.*

S5. Grazing Incidence X-ray Diffraction

Grazing incidence X-ray diffraction (GIXD) data were acquired as images. An in-house written MATLAB script was used to scale the images to q_{xy} and q_z and to interpolate the scaled images to an even step-size in q_{xy} and q_z, to facilitate comparison between images. Plots of the peaks were generated by summing the intensities for each q_{xy} over a fixed range of q_z in the MATLAB script and subtracting a background using OriginPro. The peaks were fitted in OriginPro using a Voigt function. The peak widths were comparable with the estimated resolution of 0.01 Å^{-1}. Additional plots of smaller slices of the images were sometimes employed to aid fitting.

Images with one peak at $q_z \sim 0$ Å^{-1} were analysed as a hexagonal unit cell. The presence of two peaks or three peaks indicates a tilt of the hydrocarbon chains from the surface normal.[S7] The q_{xy} and q_z positions were found to be consistent with a rectangular unit cell (two peaks) or an oblique cell (three peaks) and were analysed accordingly. Figure S11 shows a schematic diagram of an oblique unit cell with the structural parameters defined.

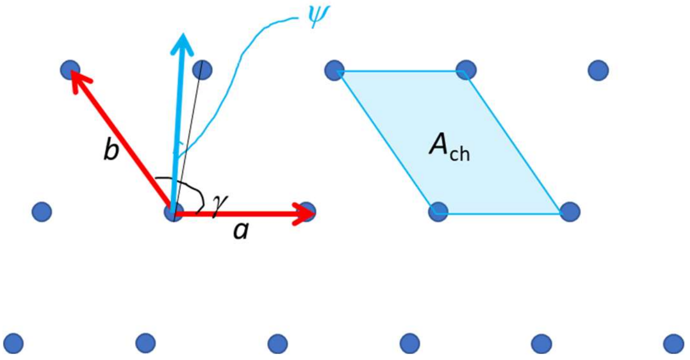

Figure S11. *Schematic diagram of the oblique unit cell with the structural parameters defined. The thick blue arrow represents the direction of the chain tilt. The shared area is the area occupied per hydrocarbon chain.*

Eq. S4 was used to determine the inter-plane spacings:

$$d_{xy,hk} = 2\pi/q_{xy,hk} \qquad \text{Eq. S4}$$

The unit cell parameters a, b and γ were calculated from the values of d.

Eq. S5 was used to estimate the tilt angle from the surface normal, t:

$$q_{z,hk} = (\boldsymbol{q_{xy,hk}} \cdot \hat{\boldsymbol{e}}) \tan(t) \qquad \text{Eq. S5}$$

where $\hat{\boldsymbol{e}}$ is the unit vector pointing along the chain tilt direction. (Note that the tilt angle is commonly denoted t in GIXD literature and is equivalent to the θ normally used in PM-IRRAS literature.) The in-plane angle ψ (for oblique unit cells) was also determined from this calculation.

Representative Bragg peak plots for each pressure are given in Figures S12–S15 and the parameters obtained from fitting are listed in Tables S2–S8. (For presentation purposes, the peak obtained for DMPE at 40 mN m^{-1} in each experiment has been used to scale peaks from two different experiments. The DMPE peak positions were comparable.) Figure S16 compares Bragg peaks for selected deuterated samples with their undeuterated analogues. These measurements were made to verify the feasibility of comparing X-ray reflectivity data with neutron reflectivity data, since the latter were made with deuterated samples (see Section S7).

Tables S2–S8 present the results of the fits to the data. The values given are the mean values obtained from fitting the replicate measurements for each sample. The standard deviations of the q_{xy} values of the replicate measurements were used to estimate the errors in the structural parameters (assuming errors in q are independent of one another). Systematic errors arising from instrument set-up have not been specifically treated. However, DMPE at 40 mN m^{-1} has been measured in several experiments to give an indication of variation in set-up. The standard deviation for the Bragg peak in these measurements is 0.0008 Å^{-1}, which represents a relative error of 5 × 10^{-4}. The DMPE data presented below are taken from reference S1 and include results measured from the same experiments as the TMCL- and DMPG-containing samples. The reported errors in tilt angles are the standard deviations in the tilt angles obtained from fitting the data of each sample. To aid comparison between samples, molecular areas are given as area per pair of chains and area per chain.

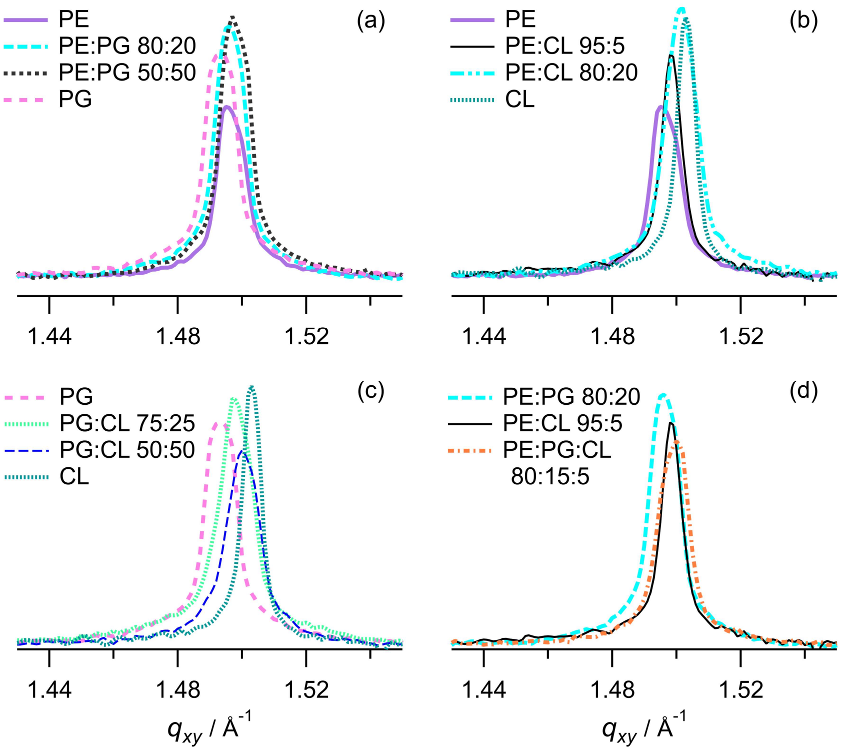

Figure S12. *Comparisons of Bragg peak plots at 40 mN m^{-1} for (a) DMPE, PE:PG 80:20, PE:PG 50:50, DMPG; (b) DMPE, PE:CL 95:5, PE:CL 80:20, TMCL; (c) DMPG, PG:CL 75:25, PG:CL 50:50, TMCL; (d) PE:PG 80:20, PE:CL 95:5, PE:PG:CL 80:15:5.*

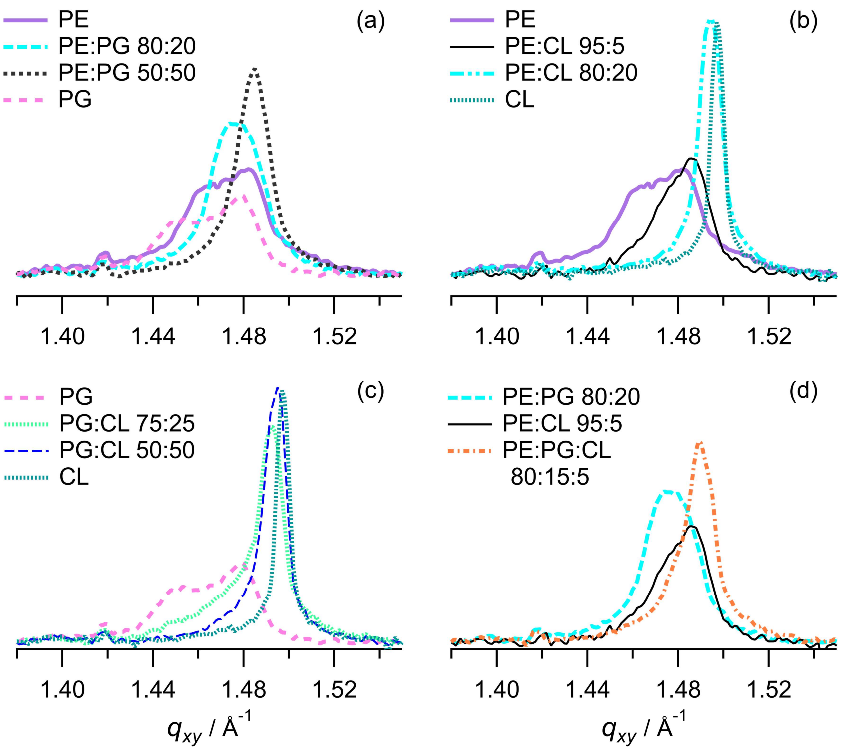

Figure S13. *Comparisons of Bragg peak plots at 23 mN m^{-1} for (a) DMPE, PE:PG 80:20, PE:PG 50:50, DMPG; (b) DMPE, PE:CL 95:5, PE:CL 80:20, TMCL; (c) DMPG, PG:CL 75:25, PG:CL 50:50, TMCL; (d) PE:PG 80:20, PE:CL 95:5, PE:PG:CL 80:15:5.*

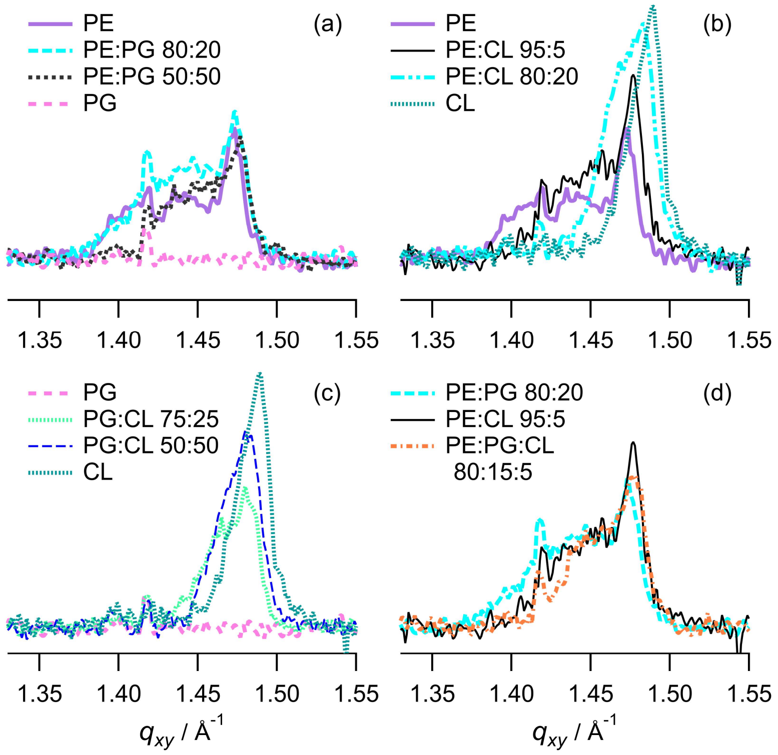

Figure S14. *Comparisons of Bragg peak plots at 10 mN m^{-1} for (a) DMPE, PE:PG 80:20, PE:PG 50:50, DMPG; (b) DMPE, PE:CL 95:5, PE:CL 80:20, TMCL; (c) DMPG, PG:CL 75:25, PG:CL 50:50, TMCL; (d) PE:PG 80:20, PE:CL 95:5, PE:PG:CL 80:15:5.*

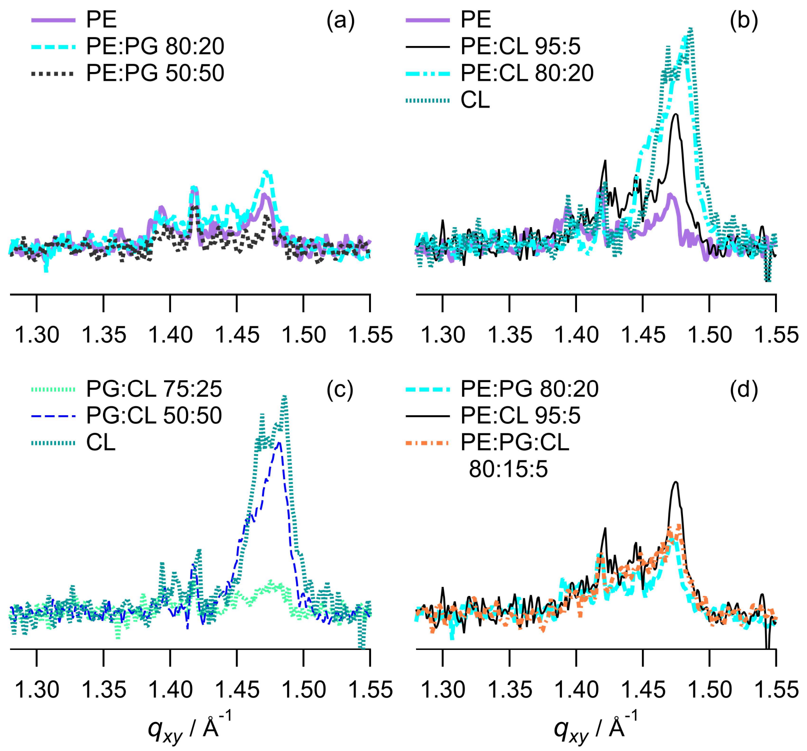

Figure S15. *Comparisons of Bragg peak plots at 6.6 mN m^{-1} for (a) DMPE, PE:PG 80:20, PE:PG 50:50, DMPG; (b) DMPE, PE:CL 95:5, PE:CL 80:20, TMCL; (c) DMPG, PG:CL 75:25, PG:CL 50:50, TMCL; (d) PE:PG 80:20, PE:CL 95:5, PE:PG:CL 80:15:5.*

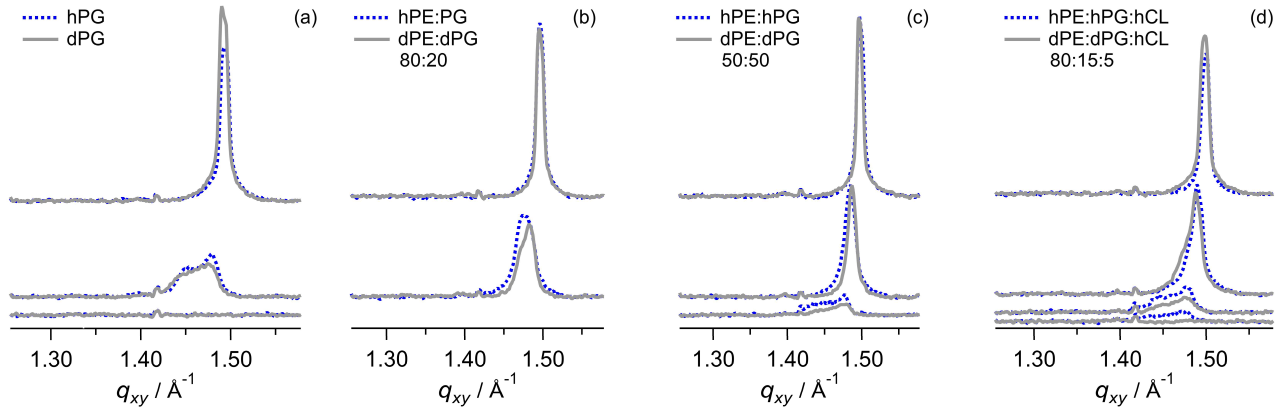

Figure S16. *Comparisons of Bragg peak plots for deuterated (grey solid lines) and undeuterated (blue dotted lines) PE/PG samples. The data at different pressures are offset for clarity. (a) DMPG top to bottom: 40, 23 and 10 mN m^{-1}; (b) PE:PG 80:20 top to bottom: 40 and 23 mN m^{-1}; (c) PE:PG 50:50 top to bottom: 40, 23 and 10 mN m^{-1}; (d) PE:PG:CL 80:15:5 top to bottom: 40, 23, 10 and 6.6 mN m^{-1}.*

Table S2. *Structural parameters calculated from the GIXD data measured at 40 mN m⁻¹. The parameters are defined in Figure S11. The data for DMPE are taken from reference S1.*

comp^n	q_{xy} / Å^{-1}	d / Å	$a = b$ / Å	γ / °	A_{pair} / Å^2<br>A_{ch} / Å^2	t / °	ψ / °
DMPE	1.496 (0.0008)	4.201 (0.002)	4.850 (0.003)	120	40.75 (0.04)<br>*20.37 (0.02)*	0	0
TMCL	1.504 (0.0005)	4.179 (0.001)	4.825 (0.002)	120	40.33 (0.03)<br>*20.16 (0.01)*	0	0
5% CL<br>95% PE	1.498 (0.0004)	4.193 (0.001)	4.842 (0.001)	120	40.60 (0.02)<br>*20.30 (0.01)*	0	0
20% CL<br>80% PE	1.501 (0.0004)	4.185 (0.001)	4.833 (0.001)	120	40.46 (0.02)<br>*20.23 (0.01)*	0	0
DMPG	1.494 (0.0008)	4.207 (0.002)	4.857 (0.003)	120	40.87 (0.04)<br>*20.43 (0.02)*	0	0
20% PG<br>80% PE	1.497 (0.0003)	4.198 (0.0009)	4.847 (0.001)	120	40.69 (0.02)<br>*20.35 (0.009)*	0	0
50% PG<br>50% PE	1.480 (0.0002)	4.195 (0.0006)	4.844<br>(0.0007)	120	40.63 (0.01)<br>*20.32 (0.006)*	0	0
5% CL<br>15% PG<br>80% PE	1.499 (0.0003)	4.192 (0.001)	4.840 (0.001)	120	40.58 (0.02)<br>*20.29 (0.01)*	0	0
25% CL<br>75% PG	1.498 (0)	4.194 (0)	4.843 (0)	120	40.62 (0)<br>*20.31 (0)*	0	0
50% CL<br>50% PG	1.500 (0.0003)	4.187 (0.0008)	4.835 (0.001)	120	40.49 (0.02)<br>*20.25 (0.008)*	0	0

Table S3. *Structural parameters calculated from the GIXD data measured at 23 mN m^{-1}. The parameters are defined in Figure S11. The data for DMPE are taken from reference S1.*

comp^n	q_{xy} / Å^{-1}	d / Å	a, b / Å	γ / °	A_{pair} / Å^2<br>A_{ch} / Å^2	t / °	ψ / °
DMPE	1.482 (0.002), 1.465 (0.002)	4.240 (0.004) 4.290 (0.007)	4.915 (0.008)	119.2 (0.01)	42.17 (0.09)<br>*21.09 (0.05)*	11.9 (0.5)	0
TMCL	1.497 (0.0006)	4.197 (0.002)	4.847 (0.002)	120	40.69 (0.03)<br>*20.34 (0.02)*	0	0
5% CL 95% PE	1.488 (0.001) 1.477 (0.002)	4.224 (0.004) 4.254 (0.006)	4.889 (0.007)	119.5 (0.01)	41.60 (0.08)<br>*20.80 (0.04)*	8.8 (0.4)	0
20% CL 80% PE	1.494 (0.0003)	4.205 (0.0008)	4.856 (0.0009)	120	40.84 (0.02)<br>*20.42 (0.01)*	0	0
DMPG	1.479 (0.001) 1.466 (0.004) 1.451 (0.004)	4.247 (0.003) 4.287 (0.011) 4.331 (0.011)	*a*=4.903 (0.012)<br>*b*=4.954 (0.012)	119.0 (0.02)	42.47 (0.15)<br>*21.24 (0.07)*	12.7 (0.6)	9.5 (1.6)
20% PG 80% PE	1.484 (0.0005) 1.473 (0.0002)	4.233 (0.001) 4.266 (0.0006)	4.901 (0.001)	119.5 (0.0009)	41.82 (0.01)<br>*20.91 (0.005)*	10.8 (0.1)	0
50% PG 50% PE	1.487 (0.001) 1.481 (0.002)	4.226 (0.004) 4.242 (0.008)	4.886 (0.009)	119.7 (0.01)	41.45 (0.11)<br>*20.73 (0.06)*	7.6 (0.2)	0
5% CL 15% PG 80% PE	1.491 (8×10^{-5}) 1.485 (0.001)	4.214 (0.0002) 4.231 (0.003)	4.872 (0.004)	119.7 (0.006)	41.23 (0.04)<br>*20.61 (0.02)*	5.1 (0.5)	0
25% CL 75% PG	1.492 (0.0004)	4.211 (0.001)	4.862 (0.001)	120	40.95 (0.02)<br>*20.47 (0.01)*	0	0
50% CL 50% PG	1.494 (0.0003)	4.206 (0.001)	4.857 (0.001)	120	40.86 (0.02)<br>*20.43 (0.01)*	0	0

Table S4. *Structural parameters calculated from the GIXD data measured at 10 mN m^{-1}. The parameters are defined in Figure S11. The data for DMPE are taken from reference S1.*

comp^n	q_{xy} / Å^{-1}	d / Å	a / Å	b / Å	γ / °	A_{pair} / Å^2<br>A_{ch} / Å^2	t / °	ψ / °
DMPE	1.472 (0.001)<br>1.441 (0.004)<br>1.414 (0.005)	4.268 (0.004)<br>4.360 (0.013)<br>4.444 (0.016)	4.935 (0.015)	5.030 (0.018)	117.9 (0.02)	43.86 (0.20)<br>*21.93 (0.10)*	18.5 (1.5)	13.4 (4.3)
TMCL	1.489 (0.001)<br>1.480 (7×10^{-6})	4.219 (0.004)<br>4.245 (2×10^{-5})	4.882 (2×10^{-5})	4.882 (2×10^{-5})	119.6 (0.0001)	41.45 (0.0003)<br>*20.73 (0.0001)*	9.3 (1.1)	0
5% CL<br>95% PE	1.477 (0.0006)<br>1.458 (0.002)<br>1.437 (0.002)	4.253 (0.002)<br>4.308 (0.006)<br>4.374 (0.006)	4.909 (0.007)	4.983 (0.007)	118.6 (0.01)	42.94 (0.09)<br>*21.47 (0.04)*	15.3 (0.7)	15.4 (1.1)
20% CL<br>80% PE	1.483 (0.0009)<br>1.471 (0.002)<br>1.458 (0.004)	4.237 (0.003)<br>4.270 (0.005)<br>4.309 (0.011)	4.891 (0.006)	4.936 (0.013)	119.2 (0.008)	42.15 (0.12)<br>*21.08 (0.06)*	11.5 (0.2)	7.2 (2.1)
DMPG	--	--	--	--	--	--	--	--
20% PG<br>80% PE	1.473 (6×10^{-5})<br>1.446 (0.002)<br>1.419 (0.003)	4.264 (0.0002)<br>4.345 (0.006)<br>4.429 (0.010)	4.926 (0.007)	5.021 (0.011)	118.1 (0.009)	43.64 (0.11)<br>*21.82 (0.06)*	15.6 (0.1)	7.9 (0.8)
50% PG<br>50% PE	1.476 (0.0001)<br>1.457 (0.0005)<br>1.431 (0.002)	4.256 (0.0003)<br>4.313 (0.002)<br>4.390 (0.006)	4.910 (0.002)	4.997 (0.007)	118.5 (0.002)	43.11 (0.06)<br>*21.55 (0.03)*	14.7 (1.0)	11.8 (4.6}
5% CL<br>15% PG<br>80% PE	1.478 (0.0003)<br>1.463 (0.002)<br>1.444 (0.003)	4.251 (0.0007)<br>4.293 (0.007)<br>4.351 (0.009)	4.904 (0.008)	4.970 (0.011)	118.9 (0.01)	42.67 (0.12)<br>*21.34 (0.06)*	13.8 (0.2)	8.7 (2.0)
25% CL<br>75% PG	1.482 (0.001)<br>1.464 (0.001)	4.241 (0.003)<br>4.293 (0.004)	4.917 (0.005)	4.917 (0.005)	119.2 (0.006)	42.22 (0.06)<br>*21.11 (0.03)*	11.6 (0.3)	0
50% CL<br>50% PG	1.484 (0.0004)<br>1.471 (0.0007)	4.234 (0.001)<br>4.272 (0.002)	4.904 (0.002)	4.904 (0.002)	119.4 (0.003)	41.90 (0.03)<br>*20.95 (0.01)*	11.0 (0)	0

Table S5. *Structural parameters calculated from the GIXD data measured at 6.6 mN m^{-1}. The parameters are defined in Figure S11. The data for DMPE are taken from reference S1.*

comp^n	q_{xy} / Å^{-1}	d / Å	a / Å	b / Å	γ / °	A_{pair} / Å^2<br>*A_{ch} / Å^2*	t / °	ψ / °
DMPE	1.469<br>(only)	4.276						
TMCL	1.485<br>(0.0009)<br>1.477<br>(0.002)<br>1.464<br>(0.002)	4.530<br>(0.003)<br>4.254<br>(0.005)<br>4.292<br>(0.005)	4.880<br>(0.006)	4.923<br>(0.006)	119.3<br>(0.009)	41.89 (0.07)<br>*20.94 (0.04)*	10.7 (0.2)	11.5 (1.5)
5% CL<br>95% PE	1.474<br>(0.0007)<br>1.450<br>(0.003)<br>1.424<br>(0.003)	4.262<br>(0.002)<br>4.333<br>(0.010)<br>4.412<br>(0.008)	4.921<br>(0.012)	5.011<br>(0.009)	118.3<br>(0.02)	43.42 (0.13)<br>*21.71 (0.07)*	19.7 (0.1)	19.0 (1.8)
20% CL<br>80% PE	1.482<br>(0.0005)<br>1.468<br>(0.002)<br>1.452<br>(0.003)	4.241<br>(0.001)<br>4.279<br>(0.007)<br>4.327<br>(0.008)	4.894<br>(0.008)	4.949<br>(0.009)	119.0<br>(0.01)	42.35 (0.20)<br>*21.18 (0.10)*	12.3 (0.2)	7.9 (0.8)
DMPG	--	--	--	--	--	--	--	--
20% PG<br>80% PE	1.472<br>(faint, one<br>meas.)							
50% PG<br>50% PE	1.473<br>(faint)	4.265						
5% CL<br>15% PG<br>80% PE	1.476<br>(0.001)<br>1.456<br>(0.004)<br>1.425<br>(0.003)	4.258<br>(0.004)<br>4.315<br>(0.012)<br>4.410<br>(0.008)	4.905<br>(0.014)	5.013<br>(0.009)	118.4<br>(0.02)	43.26 (0.15)<br>*21.63 (0.07)*	14.4 (1.7)	12.5 (3.7)
25% CL<br>75% PG	1.473 v.<br>faint	4.264	--	--	--	--	--	--
50% CL<br>50% PG	1.483<br>(0.0007)<br>1.470<br>(0.003)<br>1.457<br>(0.003)	4.238<br>(0.002)<br>4.273<br>(0.008)<br>4.314<br>(0.009)	4.893<br>(0.015)	4.939<br>(0.015)	119.1<br>(0.02)	42.21 (0.18)<br>*21.11 (0.09)*	11.5 (0.4)	7.7 (1.8)

Table S6. *Structural parameters calculated from the GIXD data of deuterated samples measured at 40 mN m⁻¹. The parameters are defined in Figure S11.*

compⁿ	q_{xy} / Å⁻¹	*d* / Å	*a* = *b* / Å	γ / °	A_{pair} / Å²<br>A_{ch} / Å²	*t* / °	ψ / °
DMPG	1.492<br>(0.0008)	4.212<br>(0.003)	4.863<br>(0.003)	120	40.97 (0.04)<br>*20.48 (0.02)*	0	0
20% PG<br>80% PE	1.496<br>(0.0002)	4.199<br>(0.0006)	4.849<br>(0.0006)	120	40.72 (0.01)<br>*20.36 0.005)*	0	0
50% PG<br>50% PE	1.497<br>(4×10^{-5})	4.197<br>(0.0001)	4.846<br>(0.0001)	120	40.68 0.002)<br>*20.34 0.001)*	0	0
5% CL<br>15% PG<br>80% PE	1.498	4.195	4.843	120	40.63<br>*20.32*	0	0

Table S7. *Structural parameters calculated from the GIXD data of deuterated samples measured at 23 mN m⁻¹. The parameters are defined in Figure S11.*

compⁿ	q_{xy} / Å⁻¹	*d* / Å	*a* / Å	*b* / Å	γ / °	A_{pair} / Å²<br>A_{ch} / Å²	*t* / °	ψ / °
DMPG	1.477<br>(0.0005)<br>1.462<br>(0.001)<br>1.441<br>(0.002)	4.253<br>(0.001)<br>4.298<br>(0.004)<br>4.360<br>(0.006)	4.906<br>(0.004)	4.976<br>(0.006)	118.8<br>(0.006)	42.78 (0.07)<br>*21.39 (0.03)*	13.1 (0.4)	8.1 (1.5)
20% PG<br>80% PE	1.485<br>(0.0003)<br>1.474<br>(0.0009)	4.232<br>(0.0009)<br>4.262<br>(0.003)	4.898<br>(0.003)	4.898<br>(0.003)	119.5<br>(0.004)	41.75 (0.04)<br>*20.88 (0.02)*	9.4 (0.6)	0
50% PG<br>50% PE	1.488<br>(0.0009)<br>1.482<br>(0.002)	4.222<br>(0.003)<br>4.238<br>(0.006)	4.881<br>(0.006)	4.881<br>(0.006)	119.7<br>(0.001)	41.38 (0.08)<br>*20.69 (0.04)*	9.6 (3.1)	0
5% CL<br>15% PG<br>80% PE	1.490<br>(0.0004)<br>1.480<br>(0.003)	4.218<br>(0.001)<br>4.244<br>(0.008)	4.881<br>(0.010)	4.881<br>(0.010)	119.6<br>(0.01)	41.43 (0.11)<br>*20.71 (0.06)*	9.3 (0.1)	0

Table S8. *Structural parameters calculated from the GIXD data of deuterated samples measured at 10 mN m⁻¹. The parameters are defined in Figure S11.*

compⁿ	q_{xy} / Å⁻¹	*d* / Å	*a* / Å	*b* / Å	γ / °	A_{pair} / Å²<br>A_{ch} / Å²	*t* / °	ψ / °
DMPG	--	--	--	--	--	--		
50% PG<br>50% PE	1.476<br>1.453<br>weak	4.256<br>4.323				Not calculated<br>{0 1} not visible		
5% CL<br>15% PG<br>80% PE	1.478<br>(0.0006)<br>1.463<br>(0.002)<br>1.445<br>(0.003)	4.251<br>(0.005)<br>4.230<br>(0.005)<br>4.360<br>(0.008)	4.907<br>(0.006)	4.968<br>(0.009)	118.9<br>(0.009)	42.68 (0.09)<br>*21.34 (0.04)*	12.3 (0.2)	5.9 (2.1)

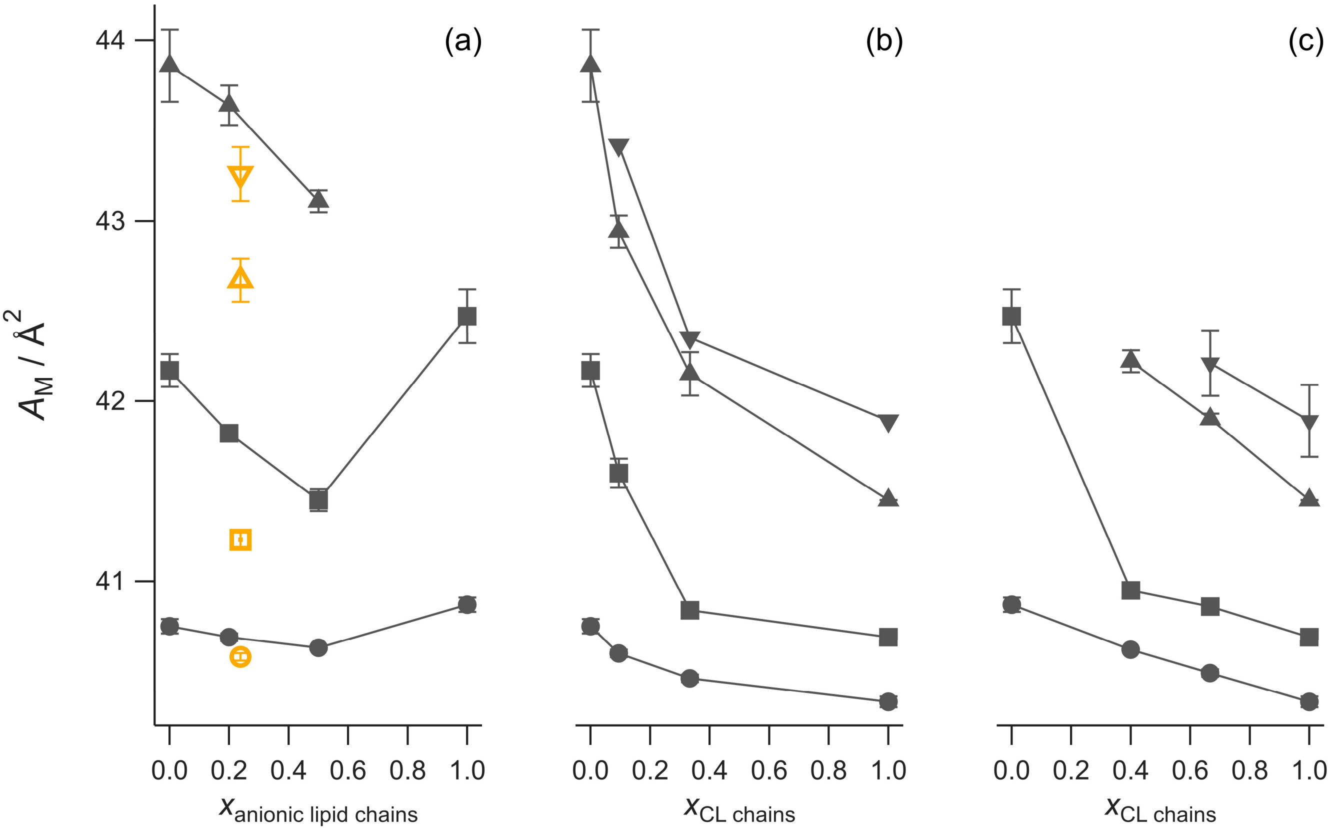

Figure S17. *(a) Area per molecule vs mole fraction of anionic lipid chains in DMPE:DMPG mixtures at 40 mN m^{-1} (filled circles), 23 mN m^{-1} (filled squares) and 10 mN m^{-1} (filled triangles). (b) Area per pair of chains vs mole fraction of CL chains in DMPE:TMCL mixtures at 40 mN m^{-1} (circles), 23 mN m^{-1} (squares), 10 mN m^{-1} (up-pointing triangles) and 6.6 mN m^{-1} (down-pointing triangles). (c) Area per pair of chains vs mole fraction of CL chains DMPG:TMCL mixtures at 40 mN m^{-1} (circles), 23 mN m^{-1} (squares), 10 mN m^{-1} (up-pointing triangles) and 6.6 mN m^{-1} (down-pointing triangles). The orange open shapes in (a) are the values for the ternary mixture at the same pressures.*

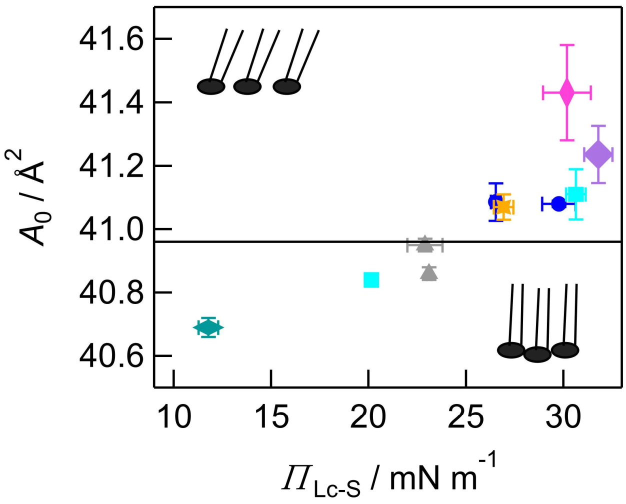

Figure S18. *Dependence of cross-sectional area at 23 mN m^{-1} on L_c–S phase transition pressure.*

S6. X-ray Reflectivity

X-ray reflectivity (XRR) data were fitted with RasCAL.[S8] The differential evolution algorithm was used to perform an initial fit to the data followed with the Bayesian MCMC algorithm. The latter reported the DIC as a measure of the fit quality. A two-slab model was employed for each case: the headgroup slab included the carbonyl groups and the tailgroup slab included the non-polar part of the chains, *i.e.* $(CH_2)_{12}CH_3$. X-ray and neutron data were

fitted separately to avoid dominance resulting from the lower errors in the XRR data but were compared after fitting to aid interpretation and selection of the most appropriate fits. The roughnesses of the interfaces between slabs were kept the same and most fits were carried out at fixed tailgroup slab scattering length density, SLD, for a series of fixed values of roughness and slab SLD. The SLD was calculated from Eq. S6:

$$\rho = \frac{\sum_i^n b_i}{V_\mathrm{m}} \qquad \text{Eq. S6}$$

Where ρ is the scattering length density, b is the scattering length and V_m is the molecular volume of the portion of the molecule within the slab (headgroup or tailgroup). X-ray scattering lengths were calculated from the total number of electrons multiplied by 2.182×10^{-5} Å. These values and the neutron scattering lengths are given in Table S9 for reference. Table S9 also includes commonly used molecular volume parameters from the literature and the corresponding SLDs. The final scattering length density of the headgroup slab depends also on the volume fraction of the solvent in the slab according to Eq. S7:

$$\rho_\mathrm{solvhg} = \phi_\mathrm{w}\,\rho_\mathrm{w} + (1-\phi_\mathrm{w})\;\rho_\mathrm{dryhg} \qquad \text{Eq. S7}$$

where ϕ_w is the volume fraction of water and ρ_w is the SLD of the solvent. ρ_solvhg is the fitted value of the slab SLD and ρ_dryhg is the SLD of a "dry" or unsolvated headgroup. For mixtures, scattering lengths were calculated from the weighted average of the components' scattering lengths. By rearranging Eq. S7 to obtain $V_\mathrm{dry,hg}$ and ϕ_w, assumptions about ideal or non-ideal mixing are avoided.[S1]

Table S9. *Reflectivity parameters.*

Slab	b / 10^{-3} Å (neutrons)	b / 10^{-3} Å (X-rays)	Literature volume / Å^3	ρ / 10^{-6} Å^{-2} (neutrons)	ρ / 10^{-6} Å^{-2} (X-rays)
Undeuterated chains ($C_{26}H_{54}$)	−2.9110	5.9178	645 (S)[S9]<br>663 (L_c)[S10]<br>780 (L_e)[S10]	−4.513 (S)<br>−4.391 (L_c)<br>−3.732 (L_e)	9.175 (S)<br>8.926 (L_c)<br>7.587 (L_e)
Deuterated chains ($C_{26}D_{54}$)	5.3303	5.9178	645 (S)[S9]<br>663 (L_c)[S10]<br>780 (L_e)[S10]	8.264 (S)<br>8.040 (L_c)<br>6.834 (L_e)	9.175 (S)<br>8.926 (L_c)<br>7.587 (L_e)
PE headgroup $C_7H_{12}NO_8P$	0.6257	3.9452	251[S9]	2.493	15.72
PG headgroup $C_6H_{12}O_{10}P$	0.7146	4.3679	289[S11]<br>291[S12]<br>346[S9]	2.473<br>2.456<br>2.066	15.11<br>15.01<br>12.62
CL headgroup $C_{13}H_{16}P_2O_{17}$	1.3549	7.3268	506[S13]<br>490[S14]	2.678<br>2.765	14.48<br>14.95
PE headgroup / D_2O (if ammonium group exchanged) $C_7H_9D_3NO_8P$	0.9378	3.9452	251[S9]	3.737	15.72
PG headgroup / D_2O (if hydroxyl group exchanged) $C_6H_{10}D_2O_{10}P$	0.9228	4.3679	289[S11]<br>291[S12]<br>346[S9]	 3.171<br>2.667	 15.01<br>12.62
CL headgroup (if hydroxyl exchanged) $C_{13}H_{15}DP_2O_{17}$	1.4590	7.3268	506[S13]<br>490[S14]	2.883<br>2.978	14.48<br>14.95

Figures S19–S27 present the XRR data with their best fits and 95% confidence ranges, along with the corresponding SLD profiles. The structural parameters derived from the fits are given in Tables S10–S12. The results from fitting each measurement are given in the tables, along with the mean values. The plots are representative examples along with their fits and 95% confidence ranges. Figure S28 gives an example of the dependence of molecular area and solvation on composition, for PE:PG mixtures at 23 mN m^{-1}. The same dependence on area is seen in the neutron reflectivity (NR) data plotted in the same figure.

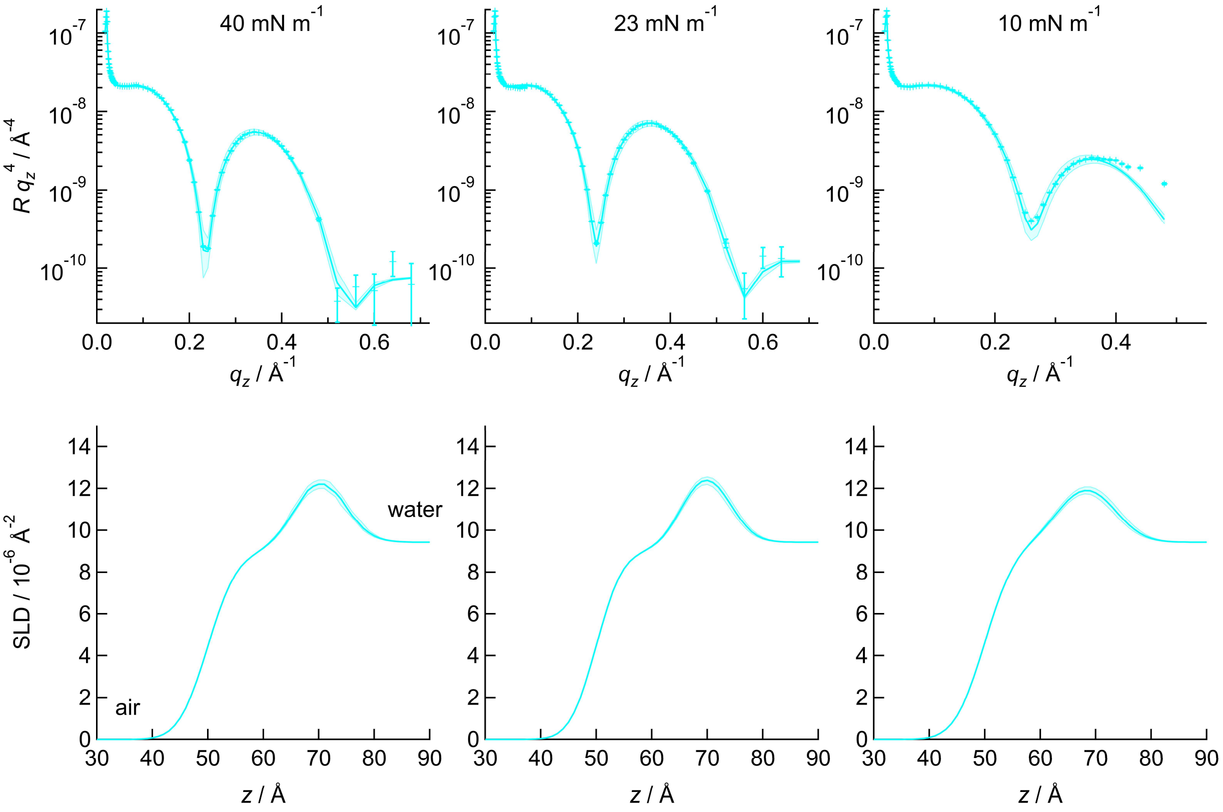

Figure S19. *XRR and corresponding SLD profiles of PE:PG 80:20 at the indicated pressures. The solid lines are fits to the data and the shaded regions represent the 95% confidence ranges.*

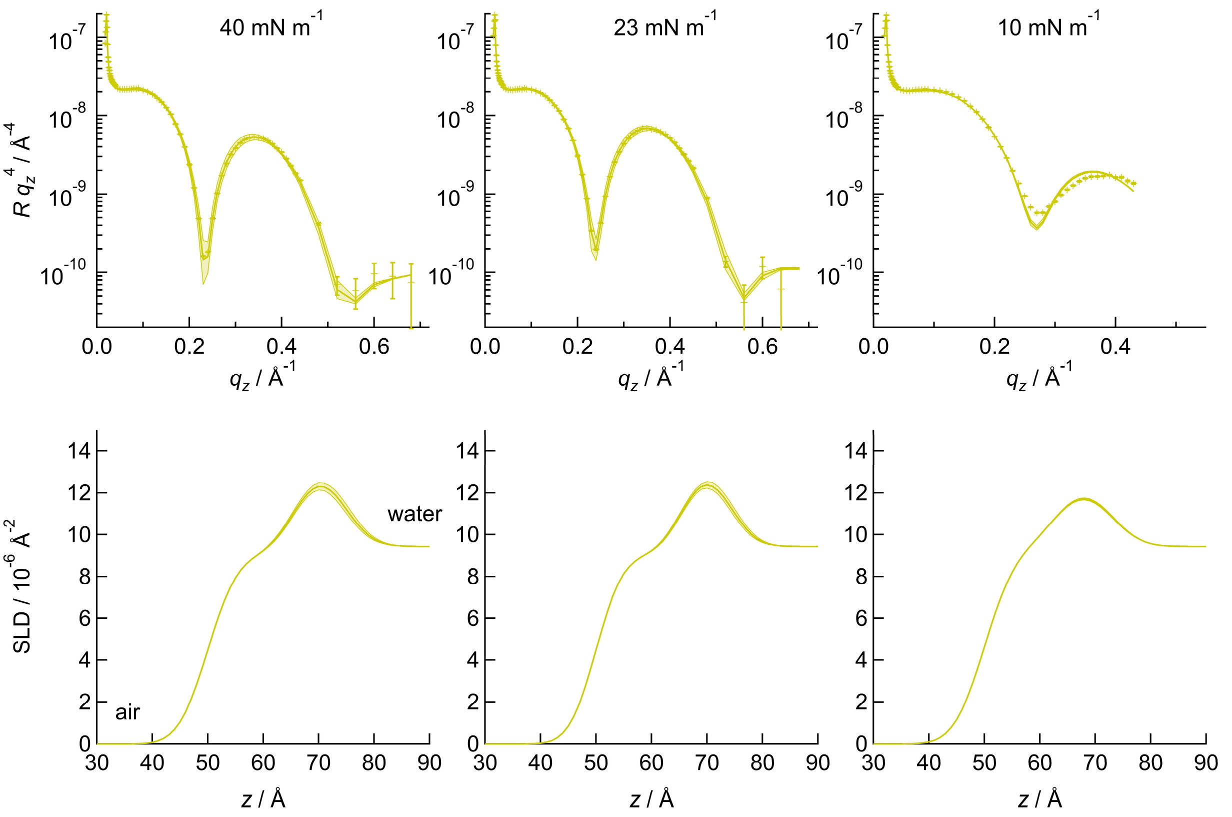

Figure S20. *XRR and corresponding SLD profiles of PE:PG 50:50 at the indicated pressures. The solid lines are fits to the data and the shaded regions represent the 95% confidence ranges.*

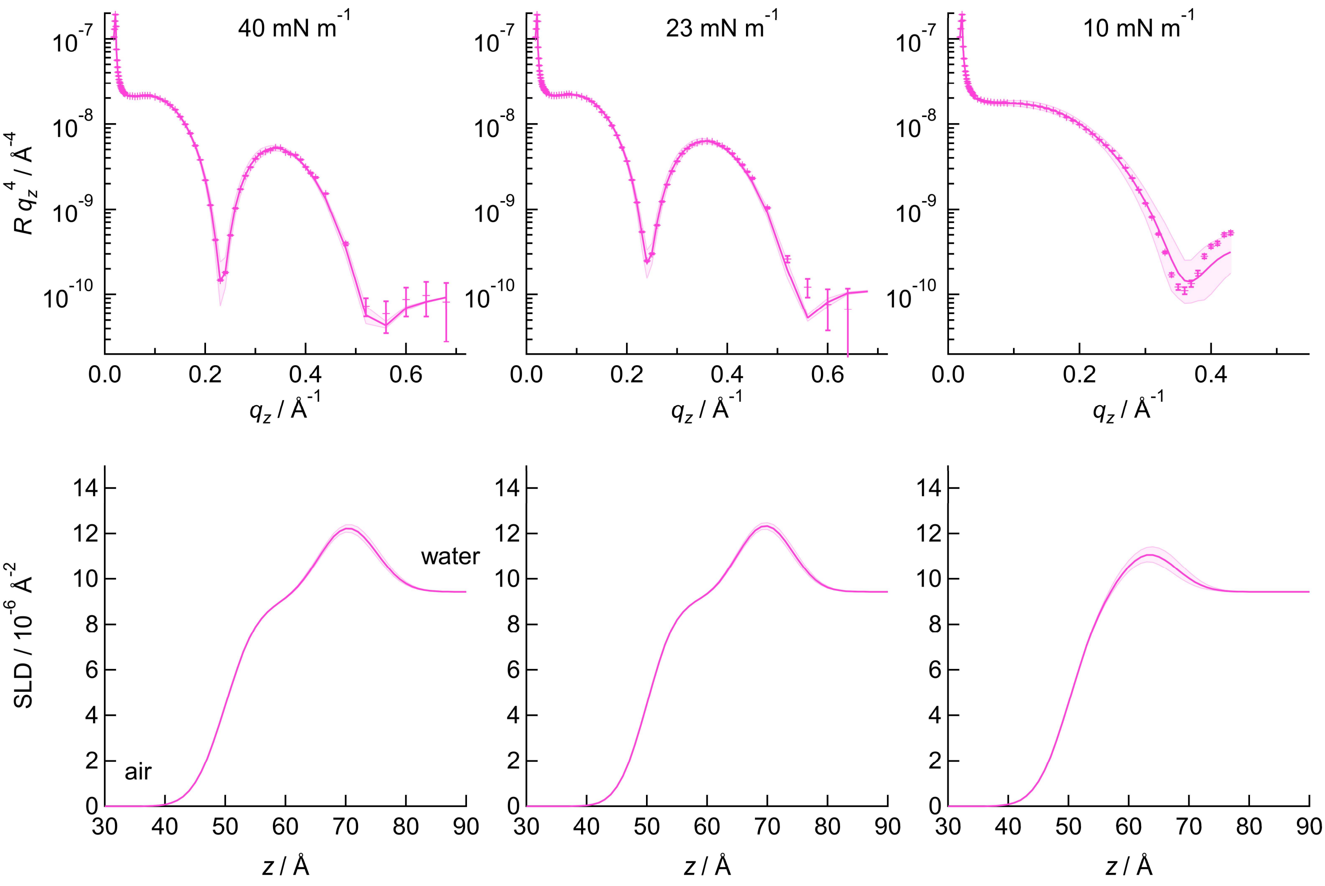

Figure S21. *XRR and corresponding SLD profiles of DMPG at the indicated pressures. The solid lines are fits to the data and the shaded regions represent the 95% confidence ranges.*

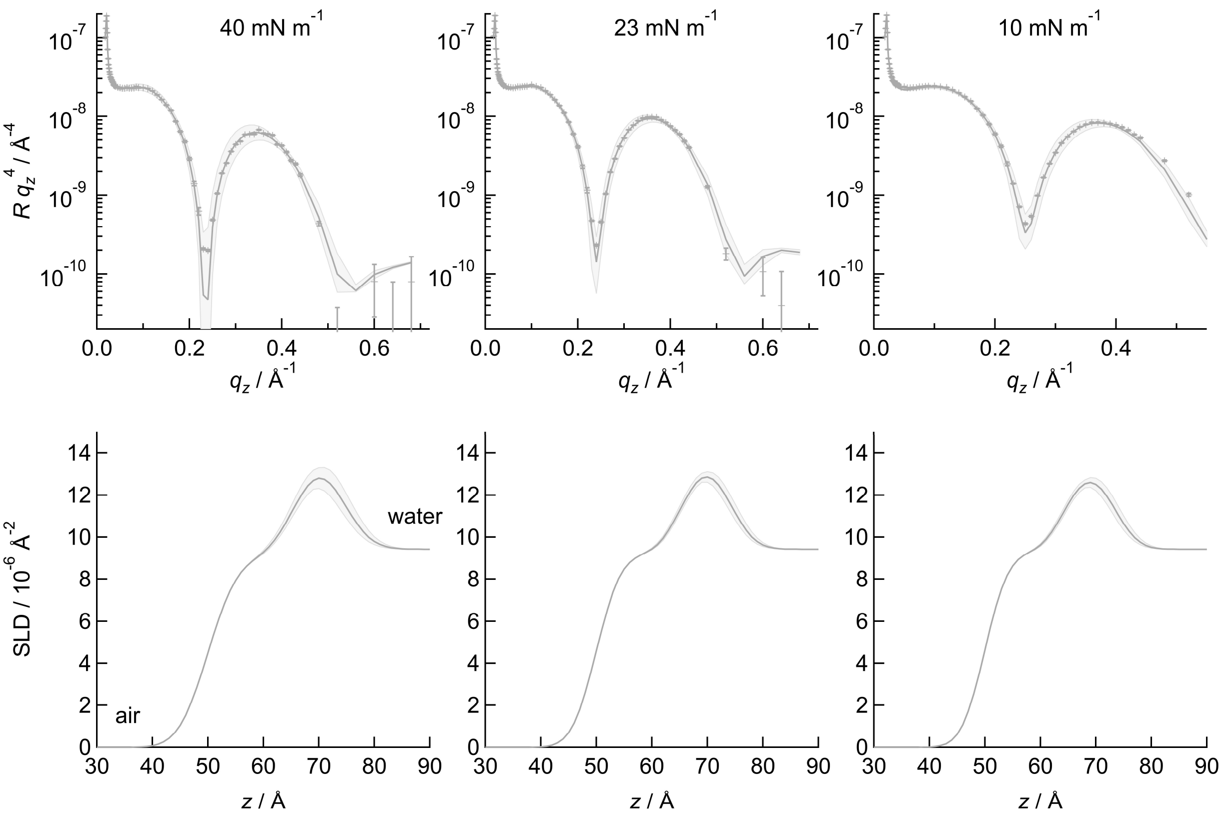

Figure S22. *XRR and corresponding SLD profiles of PE:CL 95:5 at the indicated pressures. The solid lines are fits to the data and the shaded regions represent the 95% confidence ranges.*

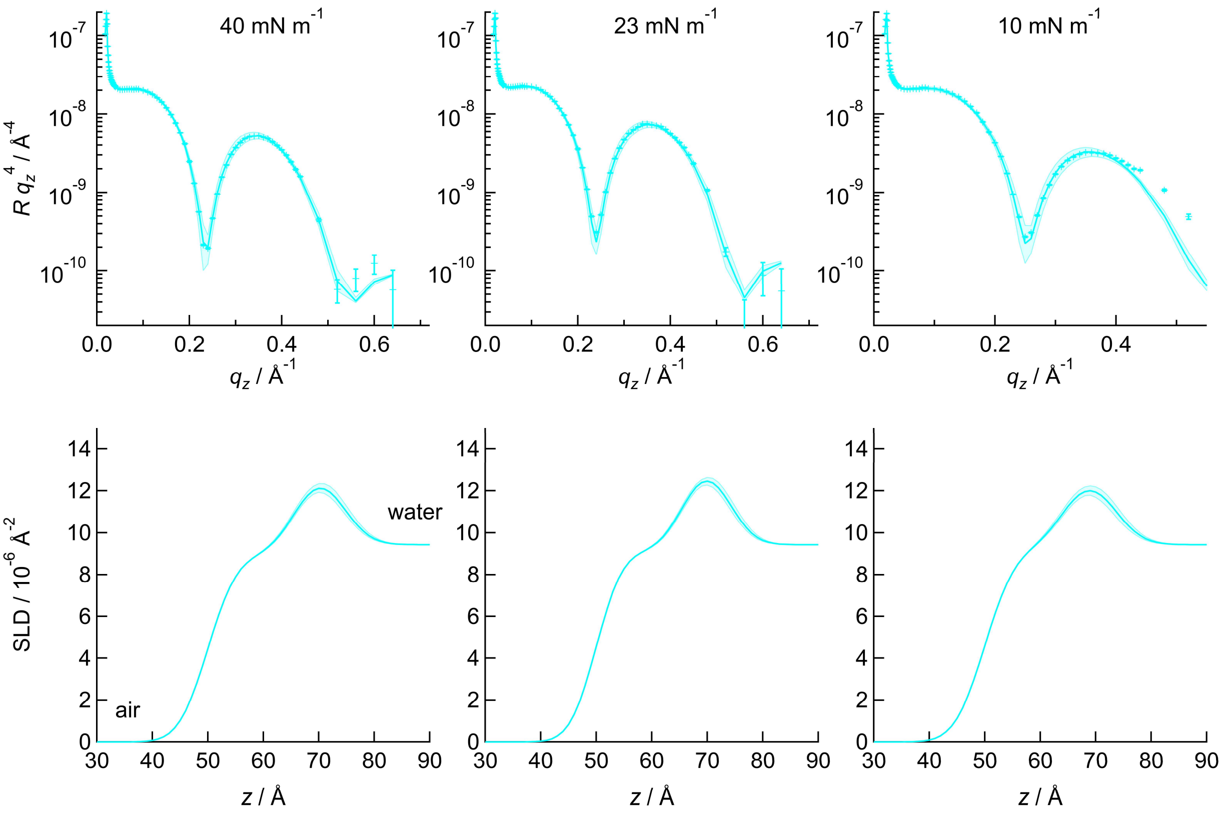

Figure S23. *XRR and corresponding SLD profiles of PE:CL 80:20 at the indicated pressures. The solid lines are fits to the data and the shaded regions represent the 95% confidence ranges.*

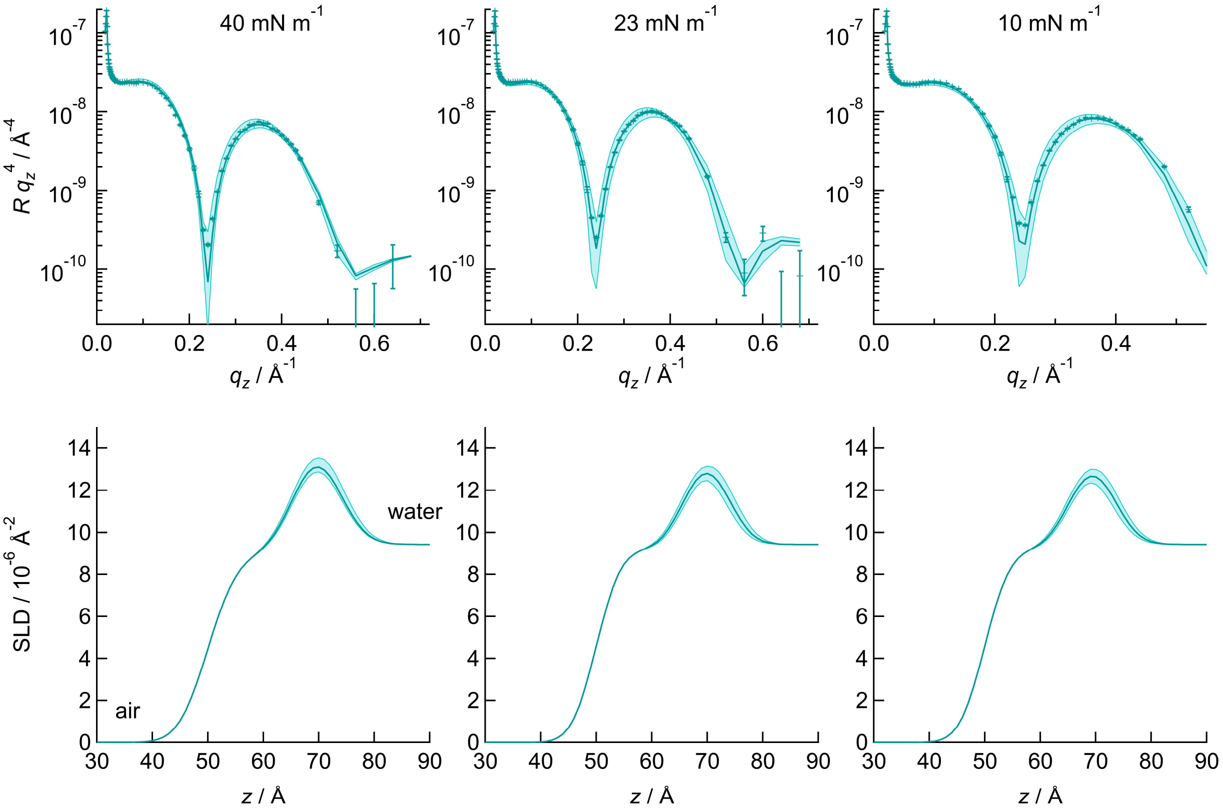

Figure S24. *XRR and corresponding SLD profiles of TMCL at the indicated pressures. The solid lines are fits to the data and the shaded regions represent the 95% confidence ranges.*

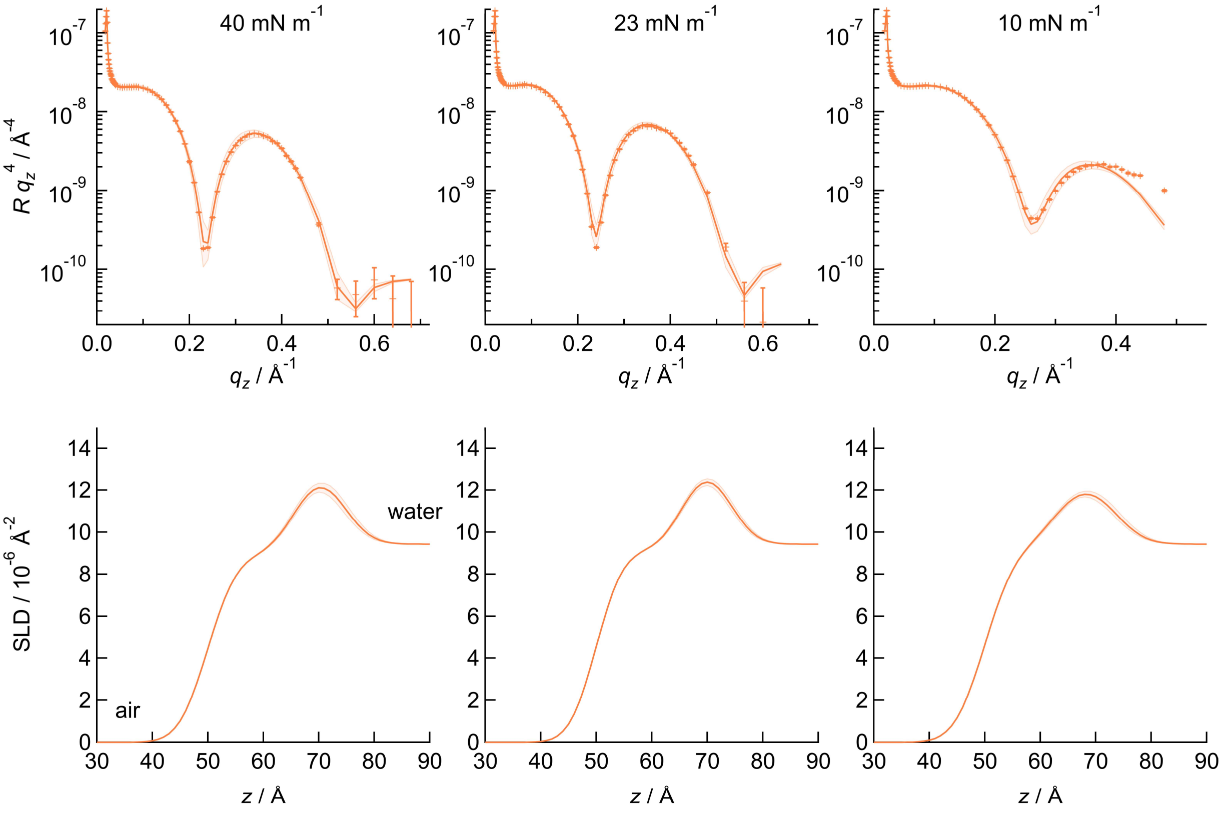

Figure S25. *XRR and corresponding SLD profiles of PE:PG:CL80:15:5 at the indicated pressures. The solid lines are fits to the data and the shaded regions represent the 95% confidence ranges.*

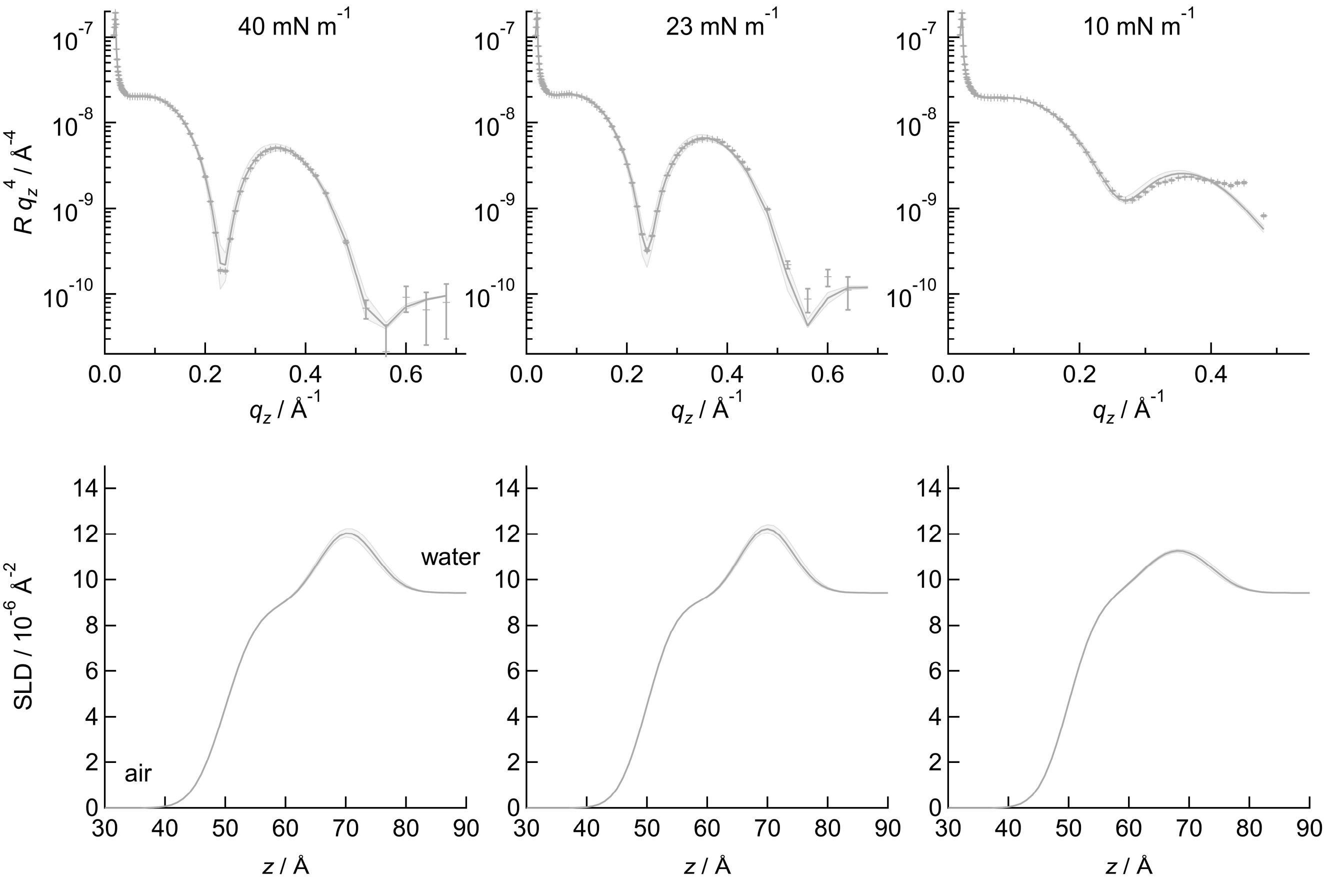

Figure S26. *XRR and corresponding SLD profiles of PG:CL 75:25 at the indicated pressures. The solid lines are fits to the data and the shaded regions represent the 95% confidence ranges.*

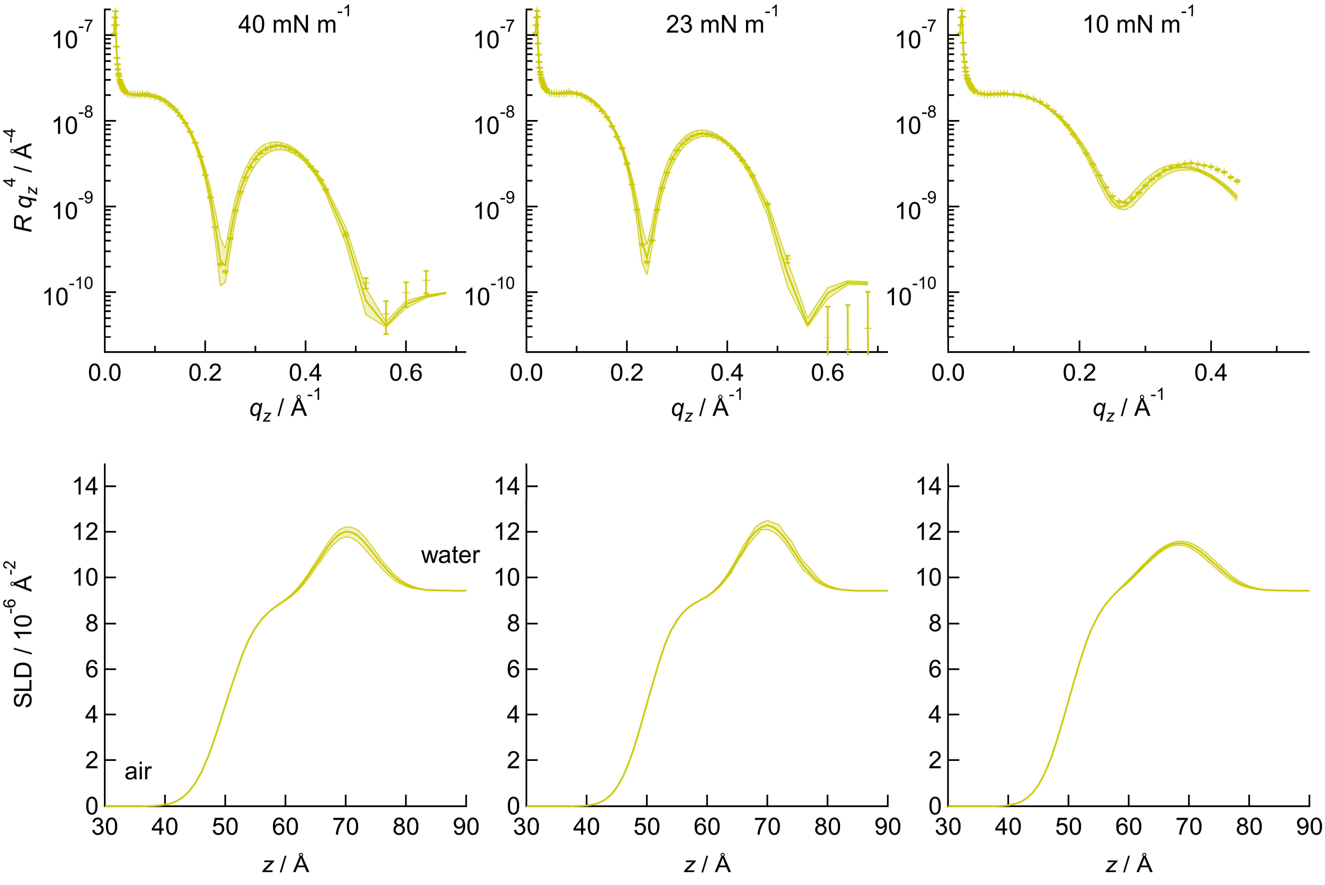

Figure S27. *XRR and corresponding SLD profiles of PG:CL 50:50 at the indicated pressures. The solid lines are fits to the data and the shaded regions represent the 95% confidence ranges.*

Table S10. *Fitted XRR parameters for different lipid compositions at 40 mN m^{-1}. σ is the roughness, V is the molecular volume and d is the thickness of the indicated slab, n_w is the number of water molecules per lipid and A_M is the molecular area. The CL-containing monolayer values are given per pair of chains to facilitate comparison. The numbers in brackets represent 95% confidence ranges from the fits.*

	σ / Å (fixed)	V_{tail} / Å^3 (fixed)	d_{tail} / Å	d_{hg} / Å	n_w	V_{hg} / Å^3	A_M / Å^2
DMPG (1)	4.2	670	16.2 (16.1–16.3)	7.9 (7.7–8.1)	0.49 (0.48–0.51)	312 (301–323)	41.3 (40.9–41.8)
DMPG (2)	4.2	665	16.2 (16.1–16.3)	8.0 (7.7–8.3)	0.59 (0.56–0.61)	211 (297–325)	41.1 (40.6–41.5)
**Ave.**	**4.2**	**668**	**16.2**	**8.0**	**0.5**	**312**	**41.2**
PE:PG 50:50 (1)	4.2	660	16.2 (16.1–16.4)	7.8 (7.5–8.1)	1.03 (0.99–1.08)	288 (273–301)	40.7 (40.2–41.1)
PE:PG 50:50 (2)	4.15	660	16.2 (16.1–16.4)	7.9 (7.6–8.2)	0.83 (0.79–0.86)	295 (282–309)	41.8 (40.2–41.1)
**Ave.**	**4.18**	**660**	**16.2**	**7.8**	**0.9**	**291**	**40.7**
PE:PG 80:20 (1)	4.15	665	16.4 (16.2–16.5)	7.4 (7.0–7.7)	0.62 (0.60–0.65)	280 (266–294)	40.6 (40.1–41.1)
PE:PG 80:20 (2)	4.2	670	16.3 (16.2–16.5)	7.4 (7.1–7.7)	0.68 (0.65–0.70)	284 (269–297)	41.0 (40.6–41.5)
**Ave.**	**4.18**	**668**	**16.4**	**7.4**	**0.7**	**282**	**40.8**
PE:CL 95:5 (1)	4.1	650	16.3 (15.9–16.6)	7.6 (6.9–8.4)	1.87 (1.68–2.05)	249 (215–285)	40.0 (39.0–40.9)
PE:CL 95:5 (2)	4.2	660	16.3 (16.0–16.6)	7.3 (6.6–8.0)	1.91 (1.73–2.10)	239 (204–273)	40.4 (39.6–41.3)
**Ave.**	**4.2**	**655**	**16.3**	**7.5**	**1.9**	**244**	**40.2**
PE:CL 80:20 (1)	4.15	670	16.5 (16.3–16.7)	6.9 (6.5–7.3)	0.38 (0.36–0.40)	269 (254–285)	40.7 (40.1–41.2)
PE:CL 80:20 (2)	4.15	665	16.4 (16.3–16.6)	7.2 (6.9–7.6)	0.80 (0.76–0.84)	268 (253–284)	40.5 (40.1–41.0)
PE:CL 80:20 (3)	4.05	660	16.4 (16.2–16.5)	7.3 (6.8–7.7)	1.16 (1.09–1.23)	259 (238–278)	40.4 (39.8–40.9)
PE:CL 80:20 (4)	4.15	660	16.4 (16.2–16.6)	7.4 (7.0–7.8)	1.24 (1.17–1.32)	260 (241–280)	40.3 (39.7–40.9)
**Ave.**	**4.13**	**664**	**16.4**	**7.2**	**0.9**	**264**	**40.5**
TMCL (1)	4.2	665	16.5 (16.3–16.7)	6.0 (6.0–6.6)	2.61 / 2 (2.36–2.87)	406 / 2 (394–461)	40.2 (39.9–40.8)
TMCL (2)	4.2	670	16.5 (16.3–16.7)	6.0 (6.0–6.5)	2.65 / 2 (2.41–2.89)	409 / 2 (397–460)	40.5 (40.2–41.1)
**Ave.**	**4.2**	**667.5**	**16.5**	**6.0**	**1.3**	**204**	**40.4**
PE:PG:CL 80:15:5 (1)	4.15	665	16.4 (16.2–16.5)	7.5 (7.1–7.8)	0.76 (0.72–0.80)	280 (263–297)	40.7 (40.1–41.2)
PE:PG:CL 80:15:5 (2)	4.15	670	16.3 (16.2–16.5)	7.4 (7.0–7.7)	0.81 (0.77–0.85)	279 (262–294)	40.9 (40.5–41.5)
**Ave.**	**4.15**	**668**	**16.3**	**7.4**	**0.8**	**280**	**40.8**
PG:CL 75:25 (1)	4.15	675	16.4 (16.2–16.5)	7.2 (6.8–7.6)	0.19 (0.18–0.20)	293 (276–308)	41.2 (40.7–41.7)

PG:CL 75:25 (2)	4.15	670	16.3 (16.2–16.5)	7.4 (7.1–7.8)	0.35 (0.33–0.37)	294 (279–309)	41.0 (40.5–41.5)
PG:CL 75:25 (3)	4.15	670	16.3 (16.1–16.6)	7.5 (7.0–7.9)	0.49 (0.46–0.52)	291 (269–309)	41.0 (40.4–41.6)
**Ave.**	**4.15**	**672**	**16.3**	**7.4**	**0.3**	**293**	**41.1**
PG:CL 50:50	4.15	670	16.4 (16.3–16.6)	7.2 (6.8–7.5)	0.48 (0.45–0.50)	280 (262–295)	40.8 (40.2–41.3)
PG:CL 50:50	4.15	672	16.5 (16.4–16.7)	6.9 (6.5–7.2)	0.01 (0.01–0.01)	278 (264–294)	40.6 (40.1–41.1)
**Ave.**	**4.15**	**671**	**16.5**	**7.0**	**0.2**	**279**	**40.7**

Table S11. *Fitted XRR parameters for different lipid compositions at 23 mN m^{-1}. σ is the roughness, V is the molecular volume and d is the thickness of the indicated slab, n_w is the number of water molecules per lipid and A_M is the molecular area. The CL-containing monolayer values are given per pair of chains to facilitate comparison. The numbers in brackets represent 95% confidence ranges from the fits.*

	σ / Å (fixed)	V_{tail} / Å^3 (fixed)	d_{tail} / Å	d_{hg} / Å	n_w	V_{hg} / Å^3	A_M / Å^2
DMPG (1)	3.8	655	15.4 (15.3–15.5)	8.3 (8.0–8.5)	1.44 (1.40–1.49)	310 (295–323)	42.6 (42.1–43.1)
DMPG (2)	3.8	660	15.4 (15.3–15.5)	7.9 (7.8–8.2)	1.57 (1.53–1.62)	292 (282–304)	42.8 (42.4–43.2)
**Ave.**	**3.8**	**658**	**15.4**	**8.1**	**1.5**	**301**	**42.7**
PE:PG 50:50 (1)	3.85	655	15.8 (15.6–15.9)	8.1 (7.9–8.5)	1.53 (1.46–1.60)	292 (278–308)	41.6 (41.1–42.1)
PE:PG 50:50 (2)	3.8	660	15.8 (15.6–15.9)	8.1 (7.9–8.4)	1.71 (1.65–1.78)	289 (275–304)	41.8 (41.4–42.3)
**Ave.**	**3.8**	**658**	**15.8**	**8.1**	**1.6**	**290**	**41.7**
PE:PG 80:20 (1)	3.8	655	15.7 (15.6–15.9)	7.8 (7.5–8.1)	1.79 (1.72–1.86)	272 (257–287)	41.6 (41.1–42.1)
PE:PG 80:20 (2)	3.75	660	15.8 (15.7–16.0)	7.7 (7.4–8.0)	1.45 (1.39–1.51)	279 (263–294)	41.7 (41.2–42.2)
**Ave.**	**3.78**	**658**	**15.8**	**7.8**	**1.6**	**276**	**41.7**
PE:CL 95:5 (1)	3.5	645	15.4 (15.2–15.7)	8.7 (8.2–9.2)	3.92 (3.67–4.16)	246 (218–275)	41.9 (41.1–42.5)
PE:CL 95:5 (2)	3.5	640	15.4 (15.2–15.6)	8.8 (8.4–9.4)	4.19 (3.92–4.46)	241 (213–272)	41.5 (40.8–42.2)
**Ave.**	**3.5**	**643**	**15.4**	**8.8**	**4.1**	**243**	**41.7**
PE:CL 80:20 (1)	3.75	650	16.0 (15.8–16.1)	7.9 (7.6–8.2)	1.91 (1.83–1.99)	264 (248–279)	40.7 (40.3–41.2)
PE:CL 80:20 (2)	3.75	650	15.9 (15.7–16.1)	7.8 (7.5–8.2)	1.97 (1.88–2.06)	261 (243–278)	40.9 (40.3–41.4)
**Ave.**	**3.75**	**650**	**15.9**	**7.8**	**1.9**	**262**	**40.8**
TMCL (1)	3.5	645	15.9 (15.6–16.2)	7.8 (7.2–8.4)	5.48 / 2 (2.51–2.97)	467 / 2 (405–531)	40.5 (39.7–41.3)
TMCL (2)	3.4	645	15.9 (15.7–16.1)	8.0 (7.5–8.4)	5.95 / 2 (2.80–3.16)	470 / 2 (416–520)	40.6 (40.0–41.2)
**Ave.**	**3.45**	**645**	**15.9**	**7.9**	**2.9**	**234**	**40.6**

PE:PG:CL 80:15:5 (1)	3.85	650	15.8 (15.7–15.9)	7.8 (7.6–8.1)	1.69 (1.62–1.77)	270 (259–287)	41.2 (40.7–41.6)
PE:PG:CL 80:15:5 (2)	3.8	650	15.9 (15.7–16.0)	7.9 (7.6–8.2)	1.66 (1.60–1.73)	275 (260–289)	40.9 (40.4–41.4)
**Ave.**	**3.83**	**650**	**15.8**	**7.9**	**1.7**	**273**	**41.0**
PG:CL 75:25 (1)	3.8	655	16.0 (15.8–16.1)	7.5 (7.2–7.9)	0.46 (0.44–0.49)	295 (282–312)	41.0 (40.5–41.5)
PG:CL 75:25 (2)	3.8	660	16.1 (16.0–16.2)	7.5 (7.4–7.7)	0.75 (0.73–0.77)	287 (280–295)	41.1 (40.7–41.5)
PG:CL 75:25 (3)	3.8	655	16.0 (15.9–16.2	7.3 (6.9–7.6)	0.0 (0.0–0.0)	299 (284–312)	40.9 (40.4–41.4)
**Ave.**	**3.8**	**657**	**16.0**	**7.5**	**0.4**	**294**	**41.0**
PG:CL 50:50	3.75	660	16.1 (15.9–16.2)	7.3 (7.0–7.7)	0.93 (0.88–0.97)	273 (259–289)	41.0 (40.6–41.5)
PG:CL 50:50	3.75	660	16.1 (15.9–16.2)	7.5 (7.2–7.8)	1.17 (1.17–1.18)	272 (244–286)	41.0 (40.6–41.6)
**Ave.**	**3.75**	**660**	**16.1**	**7.4**	**1.0**	**273**	**41.0**

Table S12. *Fitted XRR parameters for different lipid compositions at 10 mN m^{-1}. σ is the roughness, V is the molecular volume and d is the thickness of the indicated slab, n_w is the number of water molecules per lipid and A_M is the molecular area. The CL-containing monolayer values are given per pair of chains to facilitate comparison. The numbers in brackets represent 95% confidence ranges from the fits.*

	σ / Å (fixed)	V_{tail} / Å^3 (fixed)	d_{tail} / Å	d_{hg} / Å	n_w	V_{hg} / Å^3	A_M / Å^2
DMPG	4.25	655	9.7 (9.4–10.2)	6.8 (6.0–7.5)	4.6 (4.1–5.2)	318 (246–387)	67.3 (64.4–69.7)
PE:PG 50:50 (1)	4.35	640	13.0 (13.0–13.03)	9.5 (9.4–9.6)	6.02 (5.93–6.11)	288 (278–297)	49.2 (48.8–49.6)
PE:PG 50:50 (2)	4.35	640	13.0 (13.0–13.04)	9.6 (9.5–9.7)	6.29 (6.20–6.38)	283 (274–292)	49.2 (48.8–49.6)
PE:PG 50:50 (3)	4.3	640	13.0 (13.0–13.05)	9.1 (9.0–9.2)	5.13 (5.05–5.21)	296 (286–305)	49.2 (48.8–49.6)
**Ave.**	**4.3**	**640**	**13.0**	**9.4**	**5.8**	**289**	**49.2**
PE:PG 80:20 (1)	4.2	635	13.2 (13.0–13.3)	10.0 (9.7–10.3)	7.62 (7.35–7.88)	256 (229–280)	48.2 (47.5–48.9)
PE:PG 80:20 (2)	4.3	640	13.5 (13.3–13.7)	9.5 (9.1–9.9)	6.23 (5.91–6.55)	264 (231–296)	47.4 (46.5–48.2)
**Ave.**	**4.25**	**638**	**13.3**	**9.8**	**6.9**	**260**	**47.8**
PE:CL 95:5 (1)	3.30	640	14.4 (14.1–14.6)	8.9 (8.4–9.5)	5.15 (4.82–5.48)	243 (210–278)	44.5 (43.7–45.3)
PE:CL 95:5 (2)	3.35	640	14.4 (14.1–14.6)	9.0 (8.5–9.6)	5.09 (4.76–5.42)	250 (215–286)	44.6 (43.7–45.4)
**Ave.**	**3.33**	**640**	**14.4**	**9.0**	**5.1**	**247**	**44.6**
PE:CL 80:20 (1)	4.4	640	14.8 (14.6–15.0)	7.7 (7.2–8.2)	2.30 (2.14–2.45)	264 (239–290)	43.2 (42.5–43.9)

PE:CL 80:20 (2)	4.3	645	14.9 (14.7–15.1)	7.7 (7.3–8.1)	2.37 (2.22–2.51)	262 (239–286)	43.2 (42.6–43.9)
**Ave.**	**4.35**	**643**	**14.9**	**7.7**	**2.3**	**263**	**43.2**
TMCL (1)	3.6	645	15.6 (15.3–15.9)	7.4 (6.7–7.9)	5.23 / 2 (2.41–2.82)	455 / 2 (386–512)	41.5 (40.6–42.3)
TMCL (2)	3.6	645	15.5 (15.2–15.7)	7.7 (7.2–8.3)	6.07 / 2 (5.61–6.52)	464 / 2 (404–524)	41.7 (41.0–42.5)
**Ave.**	**3.6**	**645**	**15.5**	**7.6**	**2.8**	**230**	**41.6**
PE:PG:CL 80:15:5 (1)	4.3	640 (640–642)	13.0 (12.8–13.2)	10.4 (9.9–10.9)	8.71 (8.30–9.12)	254 (215–290)	49.4 (48.4–50.3)
PE:PG:CL 80:15:5 (2)	4.35	640 (640–643)	13.1 (12.9–13.3)	10.1 (9.8–10.6)	7.99 (7.57–8.40)	256 (223–293)	48.8 (48.0–49.8)
**Ave.**	**4.33**	**640**	**13.0**	**10.3**	**8.3**	**255**	**49.1**
PG:CL 75:25 (1)	3.9	640	12.4 (12.2–12.6)	11.4 (11.0–11.9)	9.87 (9.48–10.26)	298 (262–333)	51.8 (50.9–52.7)
PG:CL 75:25 (2)	3.8	640	11.2 (11.1–11.3)	13.0 (12.5–13.0)	15.94 (15.74–16.13)	268 (233–282)	57.3 (56.4–58.0)
**Ave.**	**3.85**	**640**	**11.8**	**12.2**	**12.9**	**283**	**54.6**
PG:CL 50:50 (1)	3.9	640	12.6 (12.4–12.8)	11.4 (11.0–11.8)	10.59 (10.20–10.98)	263 (227–295)	50.8 (49.8–51.5)
PG:CL 50:50 (2)	3.9	640	12.7 (12.6–12.9)	11.5 (11.2–11.9)	10.89 10.55–11.23)	255 (227–284)	50.4 (49.6–51.1)
PG:CL 50:50 (3)	3.9	640	12.4 (12.2–12.5)	12.0 (11.7–12.4)	12.5 (12.08–12.86)	251 (219–282)	51.8 (51.1–52.6)
**Ave.**	**3.9**	**640**	**12.6**	**11.7**	**11.3**	**256**	**51.0**

S7. Neutron Reflectivity

A two-slab model was used, as for XRR analysis. The data were analysed with the RasCAL "standard model", with fixed roughness and tailgroup slab SLD. The variation of the error in the fit is smaller than with our previous DMPE:DMPS mixtures[S1] and there was sometimes little certainty in the SLD of the headgroup slab in ACMW. The fit with a reasonable molecular area and with comparable solvated headgroup volumes in the two contrasts was selected and was usually the closest or next closest fit. Some of the data at 10 mN m^{-1} could not be fitted with this approach. A re-parameterised model was also tried, in which the roughness, molecular area and water content were allowed to vary, being defined in terms of the thicknesses and SLDs. The SLDs were calculated using the scattering lengths in Table S9 and fixed molecular volume elements. The unsolvated headgroup volume values were taken from the XRR fitted values and fits were made over a range of different fixed molecular tail volumes. This model was successful for DMPG but the tail volume was found to be smaller than normally reported for the L_e phase. This finding suggests the layer is thinner on expansion rather than less dense. For PE:PG 80:20, the error decreased steadily with increasing tail volume until the limit of the allowed roughness range (3.0 Å) was reached at 730 Å^3; the resulting tailgroup slab thickness was slightly higher than that fitted for the 23 mN m^{-1} sample. A sudden change in tail volume in the same phase and an increase in tail thickness at lower pressure is unlikely to be physically reasonable, so the best of the fits from the standard model has been chosen for the 10 mN m^{-1} sample as well. The molecular area obtained with this fit is within the narrow range obtained with the re-parameterised model.

The results for the ternary mixture were fitted using both methods; with the re-parameterised model there was a similar tendency to favour increasing tail molecular volume as observed for PE:PG 80:20. Three headgroup volumes (taken from the XRR fits) and four different scattering lengths corresponding to different degrees of hydrogen/deuterium exchange were tested in the re-parameterised model. The molecular areas were all in the

range 41.3–41.7 Å^2 at 40 mN m^{-1} and 43.0–43.3 Å^2 at 23 mN m^{-1}. The error decreases with increasing tail molecular volume until the lower boundary for roughness (3.0 Å) or water molecules (0) is reached and then begins to increase. Finally, the fits were repeated using fixed roughness values taken from XRR data at a series of tail volume values at 40 mN m^{-1}. The closest fit was found at 675 Å^3 for each headgroup volume and scattering length combination at 40 mN m^{-1}. This value is close to the value obtained with XRR and to the preferred fit using the standard model, which in turn suggests both PG and CL exchange hydroxyl hydrogen atoms with the subphase and PE exchanges up to one hydrogen atom.

For the binary PE:CL mixture, both fitting approaches give a lower overall thickness than was obtained for XRR. The tail slab has lower scattering length than in the other binary mixtures because CL was undeuterated and this might reduce the accuracy in the fitting. Recalculating fitted parameters using different scattering lengths (to allow for error in composition) does not improve the viability of the fits. The molecular areas are similar between the two fitting approaches and fitting the area results in greater slab thicknesses and slightly lower water content than fitting the SLDs. Although the thicknesses obtained from fitting area per molecule are closer to those obtained from XRR data, the trends with surface pressure are not logical: the apparently closest fits predicted greater thickness at 23 mN m^{-1} than at 40 mN m^{-1}, which opposes the trend observed with both the standard model and the XRR fitting and is counter-intuitive. However, if the fits at 680 Å^3 are both selected, the thicknesses are the same, which could be acceptable (both pressures are in the solid phase). The disadvantage of the area per molecule approach for this series of samples is the weak variation of χ^2 or DIC with changes in parameter values, while the disadvantage of the standard model for PE:CL samples in particular is the low degree of certainty in the ACMW headgroup slab SLD. We suggest that combining information from both approaches and from XRR is important for these lipid systems. By using the values obtained from fitting XRR, the standard model approach in NR can give an indication of the degree of isotope exchange with the solvent. This information can be combined with headgroup parameter values from XRR to fit NR with a re-parameterised model, without making assumptions about volume, ideal/non-ideal mixing and isotope exchange. In turn, the outcome of the fits using a re-parameterised model can guide the selection of fits from the standard approach because there is a high degree of confidence in the molecular area.

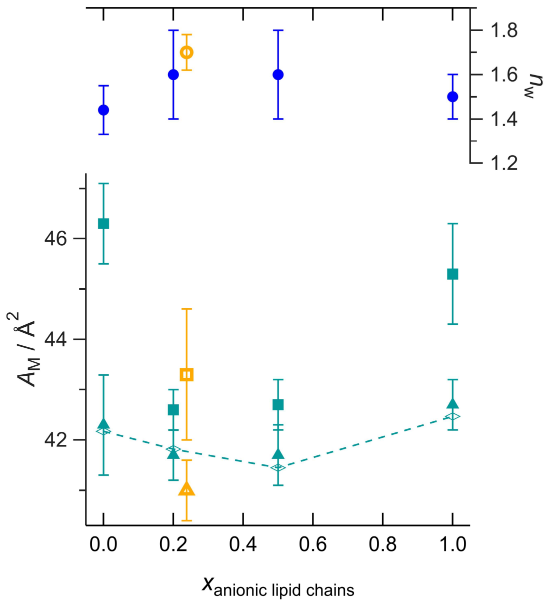

Figure S28. *Bottom: comparison of area per molecule fitted from NR data (squares) and XRR data (triangles) for DMPE:DMPG (filled teal shapes) and the ternary mixture (open orange shapes) samples at 23 mN m^{-1}. The dashed line shows the GIXD areas for DMPE:DMPG. Top: number of water molecules per headgroup (blue filled circles DMPE:DMPG, orange open circle ternary mixture).*

Table S13. *Structural parameters from the fits to the NR data at 40 mN m^{-1}. σ is roughness, V_t tail volume, d_t tail slab thickness, d_{hg} headgroup slab thickness, V_{hg} unsolvated headgroup volume, n_w water molecules per lipid, A_M area per molecule (per pair chains). The values in brackets are the 95% confidence range.*

**Lipid**<br><br>**fit**	**σ / Å**	***V*$_t$ / Å^3**	***d*$_t$ / Å**	***d*$_{hg}$ / Å**	***n*$_w$**<br>**D$_2$O SLD**<br>***ACMW SLD***	***V*$_{hg}$ (unsolv) / Å^3**	***A*$_M$ / Å^2**
PG	3.6	670	16.1<br>(15.6–<br>16.6)	7.6<br>(6.8–<br>8.4)	**0**/0.5/1.0<br>**2ex**/1ex/0ex<br>(0–1.0)<br>*0.08*<br>*(0–1.7)*	**315**/300/284<br>**2ex**/1ex/0ex<br>(284–353)	41.7<br>(40.3–43.0)
PE:PG 50:50	3.1	680	16.6<br>(16.1–<br>17.2)	7.8<br>(6.6–<br>8.8)	**1.3**/1.6/2.2<br>/2.7/1.9<br>(1.1–1.7)<br>(1.5–2.3)<br><br><br>*1.7 (0–4.7)*<br>*2.0 (0.1–5.0)*	**278**/270/253<br>/237/261<br>**all**/2ex/1ex<br>/0ex/**1PE2PG**<br>(222–333)<br>(**202–320**)	41.0<br>(39.4–42.4)
PE:PG 50:50<br>APM	3.17<br>(3.0–3.5)	685<br>(fixed)	16.7	7.0	0.1<br>(0–0.7)	292 (fixed)<br>(1PE2PG)	41.1<br>(40.7–41.5)
PE:PG 80:20	3.2	675	16.4<br>(16.0–<br>16.9)	6.2<br>(6.0–<br>7.9)	**0**/0.5/0.4<br>(0–0.5)<br><br><br><br><br>*(0–0.05)*	**255**/238<br>/**241**<br>**2ex**/1ex<br>/**1PE2PG**<br>(**245–328**)<br>(**228–319**)	41.1<br>(39.8–42.3)
PE:PG 80:20<br>APM	3.0<br>(3.0–3.3)	685<br>(fixed)	16.7	7.0	0.1<br>(0–0.6)	282 (fixed)<br>(1PE2PG)	40.9<br>(40.5–41.3)
PE:CL 80:20	3.0	670	15.3<br>(15.0–<br>15.9)	6.5<br>(6.0–<br>7.6)	**1.7**/2.1/2.4<br>**all**/2PE1CL<br>/1PE1CL<br>(1.4–2.0)<br><br>*(1.7)*<br>*(0.7–2.0)*	**231**/220/209<br>**all/**2PE1CL<br>/1PE1CL<br>0 unphysical<br>(199–292)	43.7<br>(42.1–44.7)
PE:PG:CL<br>80:15:5	4.0	680	16.3<br>(16.0–<br>16.9)	6.0<br>(6.0–<br>6.8)	0<br><br>0.3<br>(0.2–0.3)<br><br>*0*	256 (1 PE,<br>PG,CL)<br>243 (PG&CL)<br>(248–290)<br>(221–287)	41.7<br>(40.3–42.5)

Table S14. *Structural parameters from the fits to the NR data at 23 mN m^{-1}. σ is roughness, V_t tail volume, d_t tail slab thickness, d_{hg} headgroup slab thickness, V_{hg} unsolvated headgroup volume, n_w water molecules per lipid, A_M area per molecule (per pair chains). The values in brackets are the 95% confidence range.*

**Lipid**<br><br>**fit**	**σ / Å**	***V*$_t$ / Å^3**	***d*$_t$ / Å**	***d*$_{hg}$ / Å**	***n*$_w$**<br>**D_2O SLD**<br>***ACMW SLD***	***V*$_{hg}$ (unsolv) / Å^3**	***A*$_M$ / Å^2**
PG	3.1	645	14.2<br>(14.0–<br>14.5)	7.9<br>(7.1–8.9)	0.7/1.3/1.8<br>**2ex**/1ex/0ex<br>(0.6–0.9)<br>*0.1*<br>*(0–1.0)*	334/317/301<br>**2ex**/1ex/0ex<br>(295–388)	45.3<br>(44.3–46.1)
PG<br>APM<br>(closest fit)	3.1	650<br>(fixed)	14.5	8.0	1.6<br>(1.0–2.1)	312<br>(fixed)	44.7<br>(44.1–45.3)
PG<br>APM<br>(XRR tail vol)	3.2<br>(3.0–3.6)	645<br>(fixed)	14.4	8.1	1.6<br>(1.2–2.2)	312<br>(fixed)	44.7<br>(44.1–45.3)
PE:PG 50:50	3.1	640	14.9<br>(14.5–<br>15.2)	7.6<br>(6.6–8.7)	0.6/0.9/1.4<br>/2.0/**1.2**<br>all/2ex/1ex<br>/0ex/**1PE2PG**<br>(0.9–1.5)<br>(0.8–1.4)<br>*0.5 (0.3–1.6)*	306/298/282<br>/265/**290**<br>all/2ex/1ex<br>/0ex/**1PE2PG**<br>(238–350)	43.0<br>(42.0–44.1)
PE:PG 50:50<br>APM<br>(1PE2PG)	3.2<br>(3.0–3.5)	640<br>(fixed)	15.0	7.6	1.2<br>(0.6–1.7)	291<br>(fixed)	42.7<br>(42.4–43.2)
PE:PG 80:20	3.1	650	15.1<br>(14.6–<br>15.5)	7.0<br>(6.1–8.5)	0.3/**0.8**/1.3<br>/1.8/1.2<br>(0.6–1.1)<br>(0.9–1.6)<br><br><br>*0.1*<br>*(0.6–1.0)*	290/**276**/260<br>/243/**263**<br>all/**2ex**/1ex<br>/0ex/**1PE2PG**<br>(257–377)<br>(218–341)	43.0<br>(41.9–44.5)
PE:PG 80:20<br>APM<br>closest fit<br>(1PE2PG)	3.2<br>(3.0–3.5)	660<br>(fixed)	15.3	7.2	0.6<br>(0.2–1.1)	282<br>(fixed)	42.6<br>(42.2–43.0)
PE:PG 80:20<br>APM<br>XRR tail vol.<br>(1PE2PG)	3.2<br>(3.0–3.5)	650<br>(fixed)	15.5	7.1	0.9<br>(0.4–1.3)	282<br>(fixed)	42.6<br>(42.2–43.0)
PE:CL 80:20	3	660	15.3<br>(14.7–<br>15.7)	6.1<br>(6.0–7.9)	0.2/**0.6** /1.1<br>2PEex/1PEex<br>/0ex. (CL ex.)<br>(0.4–0.8)<br><br>*0.5 (0.5–1.3)*	254/ **243**/<br>229<br>2PEex/**1PEex**<br>/0ex. (CL ex.)<br>(229–330)	43.2<br>(42.0–44.8)
PE:CL 80:20<br>APM	3.0<br>(3.0–3.6)	680<br>(fixed)	15.8	6.4	0.4 (0.3–0.8)	263 fixed	43.1<br>(42.5–43.3)
PE:PG:CL<br>80:15:5	3.6	675	15.6<br>(15.3–<br>16.0)	6.0<br>(6.0–7.0)	0 (1PE, 0.3<br>PGCL only)<br><br>*0.2 (0–1.3)*	264<br>251<br>(241–294)	43.3<br>(42.0-44.3)

Table S15. *Structural parameters from the fits to the NR data at 10 mN m*$^{-1}$*. σ is roughness,* V_t *tail volume,* d_t *tail slab thickness,* d_{hg} *headgroup slab thickness,* V_{hg} *unsolvated headgroup volume,* n_w *water molecules per lipid,* A_M *area per molecule (per pair chains). The values in brackets are the 95% confidence range.*

**Lipid**<br><br>**fit**	**σ / Å**	V_t **/ Å**^{3}	d_t **/ Å**	d_{hg} **/ Å**	n_w<br>**D_2O SLD**<br>***ACMW SLD***	V_{hg} **(unsolv) / Å**^{3}	A_M **/ Å**^{2}
PG<br>APM (closest fit)	3.0<br>(3.0–3.7)	645<br>(fixed)	9.3	5.5	2.5 (1.4–3.3)	312<br>fixed	69.6<br>(68.3–70.9)
PG<br>APM (xrr tail vol.)	3.0<br>(3.0–3.6)	655<br>(fixed)	9.4	5.5	2.3 (1.3–3.3)	312<br>fixed	69.6<br>(68.3–70.9)
PE:PG 80:20	4.0	640	13.3<br>(13.0–13.5)	6.0<br>(6.0–6.6)	0/0.4/1.1<br>/0.3<br>(0.2–0.3)<br><br><br>*0.5 (0.4–1.8)*	295/279/263<br>/**282**<br>2ex/1ex<br>/0ex/**1PE2PG**<br>(275-313)	48.3<br>(47.4–49.2)
PE:PG 80:20<br>APM<br>1PE2PG	4.1<br>(3.7–4.5)	640<br>(fixed)	13.2	6.3	0.8 (0.2–1.3)	282<br>fixed	48.4<br>(47.8–48.9)
PE:CL 80:20	3.1	685	14.5<br>(13.9–14.9)	6.1<br>(6.0–7.6)	0.3 /**0.6**/1.1<br>2ex/**1ex**<br>/0ex<br>(0.4–0.8)<br>*0.6*<br>*(0.5–1.4)*	277 /**266**<br>/253<br>2ex/**1ex**/0ex<br>(252–348)	47.2<br>(45.9–49.2)
PE:CL 80:20<br>APM (closest fit)	3.0<br>(3.0–3.6)	705<br>(fixed)	15.1	6.2	0.9<br>(0.4–1.4)	263<br>fixed	46.8<br>(46.1–47.4)
PE:CL 80:20<br>APM (xrr tail vol.)	3.0<br>(3.0–3.8)	685<br>(fixed)	14.6	6.4	1.3<br>(0.7–1.8)	263<br>fixed	46.8<br>(46.1–47.4)
PE:PG:CL<br>80:15:5<br>APM<br>(1PE2PG1CL)	3.01<br>(3.0–3.5)	695<br>(fixed)	14.0	6.1	1.5<br>(1.0–2.1)	255<br>fixed	49.6<br>(49.1–50.0)

Table S16. *Structural parameters from the fits to the NR data at 6.6 mN m^{-1}. σ is roughness, V_t tail volume, d_t tail slab thickness, d_{hg} headgroup slab thickness, V_{hg} unsolvated headgroup volume, n_w water molecules per lipid, A_M area per molecule (per pair chains). The values in brackets are the 95% confidence range.*

**Lipid fit**	**σ / Å**	**V_t / Å^3**	**d_t / Å**	**d_{hg} / Å**	**n_w**	**V_{hg} (unsolv) / Å^3**	**A_M / Å^2**
PG APM	3.0 (3.0–3.8)	640 (fixed)	8.5	5.1	2.7 (1.6–3.7)	312 (fixed)	76.3 (74.7–78.0)
PE:PG 50:50 APM 1PE2PG ex	3.0 (3.0–3.7)	640 (fixed)	9.5	6.4	5.0 (4.1–5.9)	282 (fixed)	67.7 (66.7–68.5)
PE:PG 80:20 APM 1PE2PG ex	3.0 (3.0–3.4)	640 (fixed)	9.8	5.0	1.4 (0.5–2.2)	282 (fixed)	65.6 (64.7–66.5)
PE:CL 80:20 APM 1PE1CL ex	3.0 (3.0–3.8)	690 (fixed)	13.7	6.5	2.2 (1.5–2.8)	263 (fixed)	50.4 (49.6–51.2)
PE:PG:CL 80:15:5 APM (1PE2PG1CL)	3.0 (3.0–3.7)	675 (fixed)	10.9	5.6	3.0 (2.3–3.8)	255 (fixed)	61.9 (61.0–62.8)

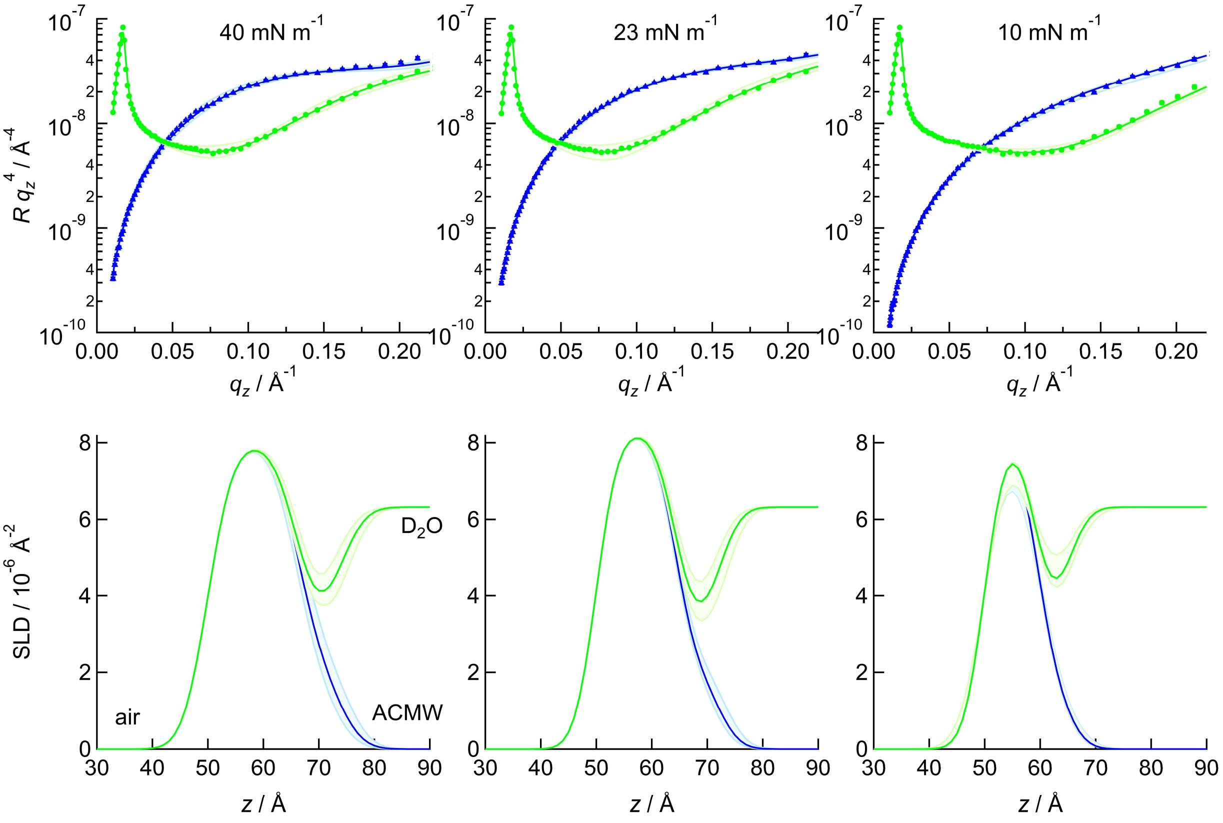

Figure S29. *NR and corresponding SLD profiles of d-DMPG at the indicated pressures. The D_2O contrast is plotted with green circles and lines and the ACMW contrast is plotted with blue triangles and lines. The solid lines are fits to the data and the shaded regions represent the 95% confidence ranges.*

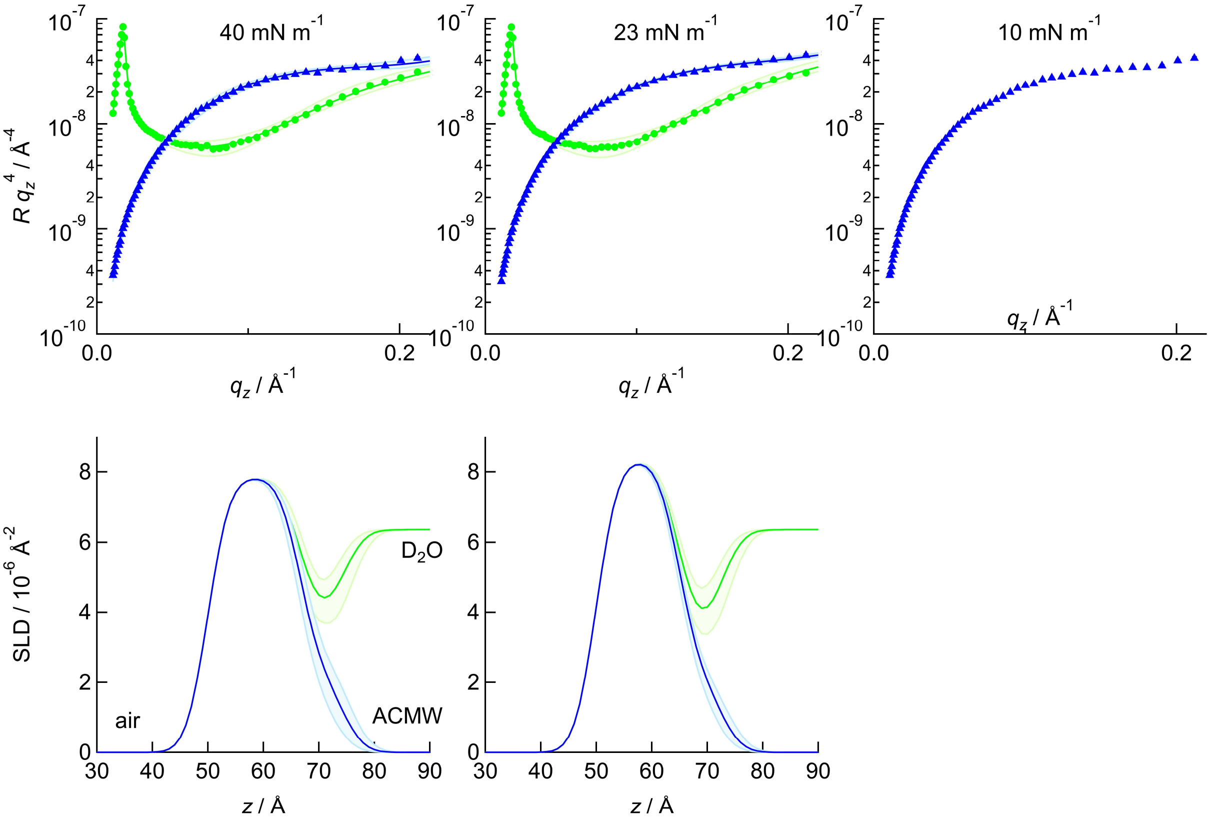

Figure S30. *NR and corresponding SLD profiles of d-PE:d-PG 50:50 at the indicated pressures. The D_2O contrast is plotted with green circles and lines and the ACMW contrast is plotted with blue triangles and lines. The solid lines are fits to the data and the shaded regions represent the 95% confidence ranges.*

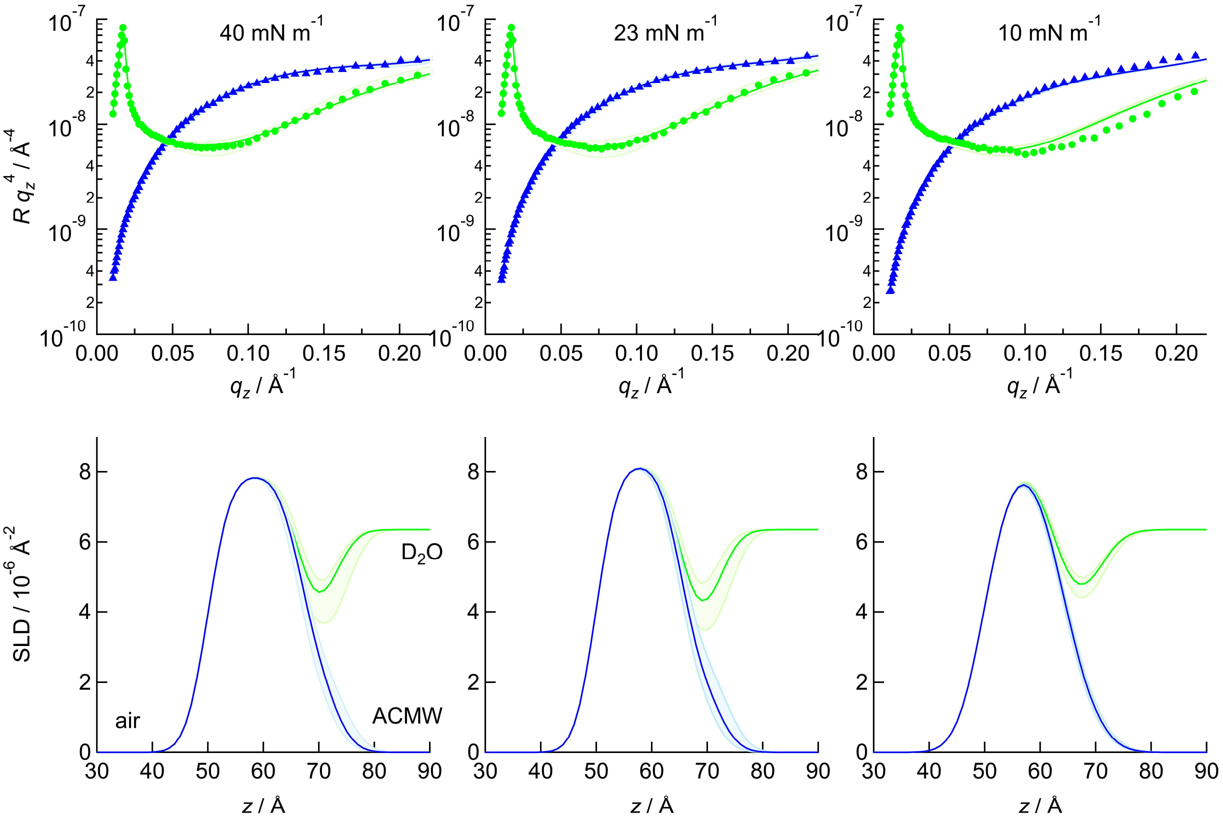

Figure S31. *NR and corresponding SLD profiles of d-PE:d-PG 80:20 at the indicated pressures. The D_2O contrast is plotted with green circles and lines and the ACMW contrast is plotted with blue triangles and lines. The solid lines are fits to the data and the shaded regions represent the 95% confidence ranges.*

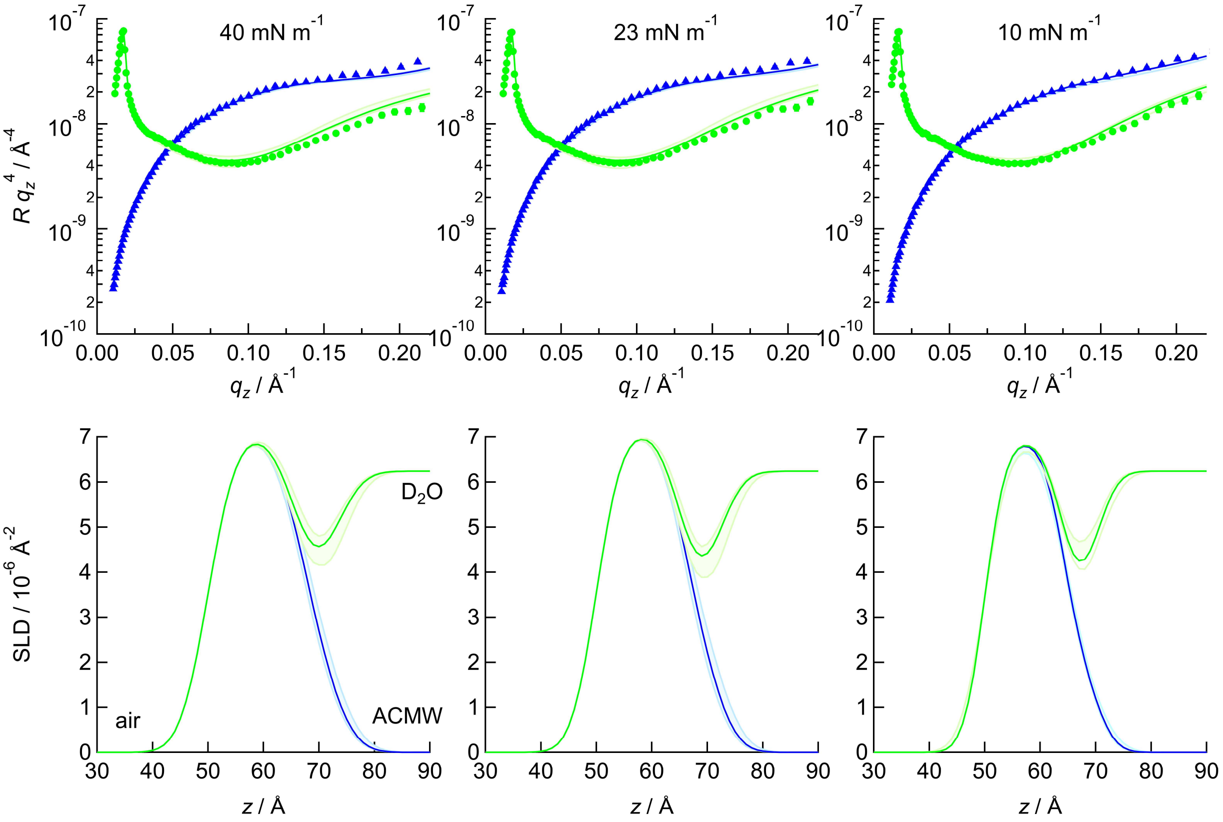

Figure S32. *NR and corresponding SLD profiles of d-PE:d-PG:h-CL 80:15:5 at the indicated pressures. The D_2O contrast is plotted with green circles and lines and the ACMW contrast is plotted with blue triangles and lines. The solid lines are fits to the data and the shaded regions represent the 95% confidence ranges.*

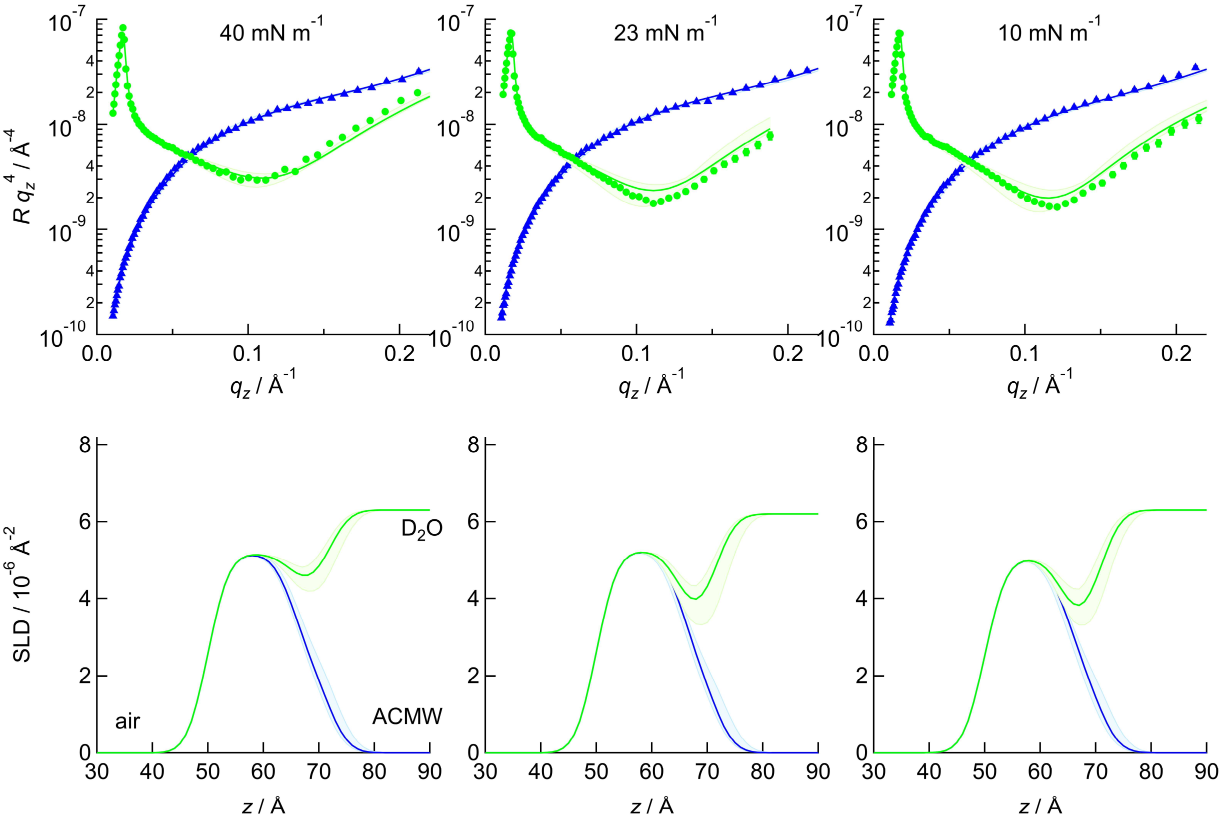

Figure S33. *NR and corresponding SLD profiles of d-PE:h-CL 80:20 at the indicated pressures. The D_2O contrast is plotted with green circles and lines and the ACMW contrast is plotted with blue triangles and lines. The solid lines are fits to the data and the shaded regions represent the 95% confidence ranges.*

References

S1. A. L. Martin, P. N. Jemmett, T. Howitt, M. H. Wood, A. W. Burley, L. R. Cox, T. R. Dafforn, R. J. L. Welbourn, M. Campana, M. W. A. Skoda, J. J. Thompson, H. Hussain, J. L. Rawle, F. Carlà, C. L. Nicklin, T. Arnold and S. L. Horswell, Effect of anionic lipids on mammalian plasma cell membrane properties. *Langmuir* 2023, **39(7)**, 2676–2691.

S2. I. Zawisza, A. Lachenwitzer, V. Zamlynny, S. L. Horswell, J. D. Goddard and J. Lipkowski, Electrochemical and photon polarization modulation infrared reflection absorption spectroscopy study of the electric field driven transformations of a phospholipid bilayer supported at a gold electrode surface. *Biophys. J.* 2003, **85(6)**, 4055‑4075.

S3. V. Zamlynny and J. Lipkowski, in *Diffraction and Spectroscopic Methods in Electrochemistry*, ed. R. C. Alkire, D. M. Kolb, J. Lipkowski and P. N. Ross, Wiley-VCH: New York, 2006, Chapter 9.

S4. I. Zawisza, X. Bin and J. Lipkowski, Potential-driven structural changes in Langmuir-Blodgett DMPC bilayers determined by in situ spectroelectrochemical PM IRRAS. *Langmuir* 2007, **23(9)**, 5180–5194.

S5. J. Umemura, T. Kamata, T. Kawai and T. Takenaka, Quantitative evaluation of molecular orientation in thin Langmuir−Blodgett films by FT-IR transmission and reflection−absorption spectroscopy. *J. Phys. Chem*. 1990, **94(1)**, 62–67.

S6. X. Bin and J. Lipkowski, Electrochemical and PM-IRRAS studies of the effect of cholesterol on the properties of the headgroup region of a DMPC bilayer supported at a Au(111) electrode. *J. Phys. Chem. B* 2006, **110(51)**, 26430–26441.

S7. J. Als-Nielsen, D. Jacquemain, K. Kjaer, F. Leveiller, M. Lahav and L. Leiserowitz, Principles and applications of grazing incidence X-ray and neutron scattering from ordered molecular monolayers at the air-water interface. *Physics Reports* 1994, **246(5)**, 251–313.

S8. A. V. Hughes, 2013, RasCAL. Sourceforge. See https://github.com/arwelHughes/RasCAL_2019. (installed Jan. 2022, last accessed Aug. 2024).

S9. D. Marsh, Molecular volumes of phospholipids and glycolipids in membranes. *Chem. Phys. Lipids*, 2010, **163(7)**, 667-677.

S10. R. A. Campbell, Y. Saaka, Y. Shao, Y. Gerelli, R. Cubitt, E. Nazaruk, D. Matyszewska and M. J. Lawrence, Structure of surfactant and phospholipid monolayers at the air/water interface modeled from neutron reflectivity data. *J. Coll. Interf. Sci*. 2018, **531**, 98–108.

S11. N. Kučerka, B. W. Holland, C. G. Gray, B. Tomberli and J. Katseras, Scattering density profile model of POPG bilayers as determined by molecular dynamics simulations and small-angle neutron and X-ray scattering experiments. *J. Phys. Chem. B* 2012, **116(1)**, 232–239.

S12. J. Pan, F. A. Heberle, S. Tristram-Nagle, M. Szymanski, M. Koepfinger, J. Katsaras and N. Kučerka,Molecular structures of fluid phase phosphatidylglycerol bilayers as determined by small angle neutron and X-ray scattering. *Biochim. Biophys. Acta* 2012, **1818(9)**, 2135–2148.

S13. A. L. Boscia, B. W. Treece, D. Mahammadyani, J. Klein-Seetharaman, A. R. Braun, T. A. Wassenaar, B. Klösgen and S. Tristram-Nagle, X-ray structure, thermodynamic,, elastic properties and MD simulations of cardiolipin/dimyristoylphosphatidylcholine mixed membranes. *Chem. Phys. Lipids*, 2014, **178**, 1–10.

S14. J. Pan, X. Cheng, M. Sharp, C-S. Ho, N. Khadka and J. Katsaras. Structural and mechanical properties of cardiolipin lipid bilayers determined using neutron spin echo, small angle neutron and X-ray scattering, and molecular dynamics simulations. *Soft Matter* 2015, **11(1)**, 130–138.
